# Isolation and characterization of a new basal-like luminal progenitor in human breast tissue

**DOI:** 10.1186/s13287-019-1361-3

**Published:** 2019-08-23

**Authors:** Vasudeva Bhat, Victoria Lee-Wing, Pingzhao Hu, Afshin Raouf

**Affiliations:** 1 Department of Immunology, Max Rady Faculty of Health Sciences, University of Manitoba, 471 Apotex Centre, 750 McDermot Avenue, Winnipeg, Manitoba R3E 0T5 Canada; 2 Research Institute for Oncology and Hematology, CancerCare Manitoba, Winnipeg, Manitoba Canada; 3 Department of Biochemistry and Medical Genetics, Max Rady Faculty of Health Sciences, University of Manitoba, Winnipeg, Manitoba Canada

**Keywords:** *NOTCH3*, *FZD7*, Bipotent progenitors, Luminal-restricted progenitors, Basal-like luminal progenitors, Luminal cell fate, Wnt7A, ERα, Notch signaling, Wnt signaling

## Abstract

**Background:**

Adult stem cells and progenitors are responsible for breast tissue regeneration. Human breast epithelial progenitors are organized in a lineage hierarchy consisting of bipotent progenitors (BPs), myoepithelial- and luminal-restricted progenitors (LRPs) where the LRP differentiation into mature luminal cells requires estrogen receptor (ER) signaling. However, the experimental evidence exploring the relationship between the BPs and LRPs has remained elusive. In this study, we report the presence of a basal-like luminal progenitor (BLP) in human breast epithelial cells.

**Methods:**

Breast reduction samples were used to obtain different subsets of human breast epithelial cell based on cell surface marker expression using flow cytometry. Loss of function and gain of function studies were employed to demonstrate the role of NOTCH3 (NR3)-FRIZZLED7 (FZD7) signaling in luminal cell fate commitment.

**Results:**

Our results suggest that, NR3-FZD7 signaling axis was necessary for luminal cell fate commitment. Similar to LRPs, BLPs (NR3^high^FZD7^high^CD90^+^MUC1^−^ER^−^) differentiate to generate NR3^med^FZD7^med^CD90^−^MUC1^+^ER^+^ luminal cells. Unlike LRPs however, BLP’s proliferation and differentiation potentials depend on NR3 and regulated in part by FZD7 signaling. Lastly, we show that BLPs have a higher colony-forming potential than LRPs and that they are continuously generated from the NOTCH3^−^FZD7^low^ subset of the bipotent progenitors.

**Conclusion:**

Our data indicate that BPs differentiate to generate basal-like luminal progenitors that in turn differentiate into LRPs. These results provide new insights into the hierarchical organization of human breast epithelial cell and how cooperation between the Notch and Wnt signaling pathways define a new progenitor cell type.

**Electronic supplementary material:**

The online version of this article (10.1186/s13287-019-1361-3) contains supplementary material, which is available to authorized users.

## Background

Human breast tissue undergoes dynamic changes of expansion and regression throughout the reproduction stages of a female. The adult breast tissue is maintained through continuous turnover of the luminal and myoepithelial cells. These mature breast epithelial cells are postulated to be produced from bipotent progenitors [1–4] that are themselves derived from primitive breast epithelial stem cells [4, 5]. However, the molecular mechanisms that regulate lineage cell commitments in human breast tissue are poorly understood. Recently, single-cell RNA sequencing was used to reconstruct the differentiation trajectories of the human breast epithelial progenitors. This analysis confirmed the previously suggested notion that bipotent progenitors (BPs) have the potential to generate luminal-restricted progenitors (LRPs) [6] and that the LRPs could give rise to both estrogen hormone-responsive and secretory luminal epithelial cells [6]. However, experimental evidence showing that BPs give rise to LRPs and the myoepithelial-restricted progenitors remain elusive. Unlike the mouse mammary gland, the distinction between human breast epithelial stem cells and the BPs is not clearly understood mainly due to lack of unique markers to distinguish these two primitive cell populations. Interestingly, single-cell RNA sequencing of mouse mammary epithelial cells identified a novel subset of mix-lineage cells in the basal cell compartment which are committed to the luminal cell fate [7], suggesting the existence of heterogeneity within in this subset of mammary epithelial cells. A mass cytometry study of human breast epithelial cells showed that the LRP subset was also heterogeneous [8]. Although these robust technologies have helped us to identify novel subset of cells in different organs [9–12], the isolation and characterization of these putative progenitor subsets in the human breast has not been possible. Such information is important since understanding the role of different progenitor subtypes in breast epithelial turnover, and the molecular mechanisms that regulate their proliferation and differentiation potentials provide new insights into how alteration to such mechanisms could result in breast cancer initiation and tumor progression [13, 14].

It was previously reported that highly conserved Notch signaling and in particular Notch3 Receptor (NR3) signaling was involved in the luminal cell-fate determination of the bipotent progenitors [1, 15] in the human breast. However, the precise role or NR3 in this process is not well understood. In this study, we report that high expression of NR3 and a Wnt ligand, FZD7, identifies a new basal-like luminal progenitor subtype whose function is reliant on Notch and Wnt signaling which is in sharp contrast to the previously identified luminal-restricted progenitors.

## Methods

### Breast reduction samples

The breast reduction samples were obtained through informed written consent (University of Manitoba, Research Ethics Board #H2010:292) and were dissociated into organoid-enriched fractions and subsequently turned into single-cell suspension as described before [16]. The vascular endothelial and immune cells (lineage positive cells, CD31^+^CD45^+^) were immunomagnetically removed using the EasySep™ Biotin Positive Selection Kit (StemCell Technologies Inc.). The lineage negative (~ 3 × 10^6^ cells) cells were placed in short cocultures with mouse fibroblasts, NIH3T3 (3566 cells/cm^2^) for 4 days in collagen-coated tissue culture dishes (collagen diluted 1:30 in PBS). For some experiments, non-cultured single-cell suspensions from freshly dissociated breast reduction samples were used.

### Isolation of different progenitor subsets

Single-cell suspensions were prepared from 4-day cultures of breast reduction samples, and Epithelial Cell Adhesion Molecule positive (EpCAM^+^) cells were immunomagnetically isolated (> 90% purity, Additional file 1: Figure S4C) as described before [1] and cells were stained with anti-α6 integrin (CD49f), conjugated to alexafluor 647 (clone# G0H3, Biolegend.), anti-CD90, conjugated to phycoerythrin (PE) (clone# 5E10, Biolegend), anti-NOTCH3 (clone# 603532, R&D systems), and anti-FZD7 (clone# 151143, R&D systems) antibodies. A goat anti-rat fluorescein isothiocyanate (FITC) was used to detect FZD7 protein, a goat anti-mouse Alexa Fluor 405 (Invitrogen) was used to detect the NR3 protein, and propidium iodide (PI) exclusion was used to identify live cells. The bulk bipotent progenitors (BP, EpCAM^+^CD49f^+^CD90^+^), BP-subset “a” (EpCAM^+^CD49f^+^CD90^+^ NR3^high^FZD7^+^), BP-subset “b” (EpCAM^+^CD49f^+^CD90^+^NR3^−/low^FZD7^+^), BP-subset “ab” (EpCAM^+^CD49f^+^CD90^+^NR3^medium^FZD7^+^), bulk luminal-restricted progenitors (LRPs, EpCAM^+^CD49f^+^CD90^−^), LRP-subset “a” (EpCAM^+^CD49f^+^CD90^−^NR3^+^FZD7^+^), and the LRP-subset “b” (EpCAM^+^CD49f^l+^CD90^−^NR3^−^FZD7^+^) were isolated via Fluorescent Activated Cell Sorting (FACS).

### Non-malignant human breast epithelial cell lines

The 184-hTert cells were a kind gift from Dr. Samuel Aparicio (B.C. Cancer Agency, Vancouver B.C. Canada [17]). Cells were maintained in the growth medium DMEM:F12 1:1 media supplemented with 10 ng EGF, 25 μg Insulin, 500 μg hydrocortisone, 2.5 ng sodium selenate, 400 μg G418, 0.15 U prolactin, 1.6 μg transferrin, and 10 nM isoproterenol and were subcultured every 2–3 days (before reaching confluency). Both the cell types were maintained in a humidified chamber at 37°c and 5% CO2.

### Protein expression analysis

Flow cytometry was used to examine protein expression using specific antibodies raised against NR3, FZD7, and GABRE (Alomone Labs). For this purpose, single-cell suspensions were prepared from the breast reduction samples or 184-hTert cells as described [15]. NR3 protein (intracelluar domain) expression was detected using a goat anti-mouse antibody conjugated to PE (Biolegend), FZD7 protein expression was detected using a goat anti-rat antibody conjugated to 647 (Jackson ImmunoResearch lab Inc.) and the GABRE protein was detected using a goat anti-rabbit antibody conjugated to PE (Invitrogen) and quantified using the FlowJo Software (TreeStar Inc.).

### Molecular cloning of full-length FZD7 gene

The cDNA representing full-length *FZD7* transcript was PCR cloned using primers flanking AscI and PacI restriction endonuclease cut sites (Additional file 2: Table S3). The amplified PCR fragments were size verified on agarose gels and digested with ASCI and PACI restriction endonucleases and ligated into the AscI-PacI site of the lentiviral vector, KA391 [1]. The overexpressed *FZD7* gene was verified at transcript (Additional file 3: Figure S7A) and the protein levels (Additional file 3: Figure S7B).

### Lentiviral transduction

The 184-hTERT cells or the primary breast reduction samples were made into single-cell suspensions and transduced with lentivirus to constitutively express the active (intracellular domain, ICD) form of each Notch receptor or the empty Green Fluorescent Protein (GFP) expressing virus or scrambled shRNA (scr), shNOTCH3, or shFZD7 as described before [15, 18]. The transduced cells were isolated via FACS based on their expression of GFP and cultured as described and examined for the expression of NR3 and FZD7 protein via flow cytometry.

### Colony-forming cell (CFC) assay

Different progenitor subsets were obtained from either freshly dissociated or 4-day cultures of breast reduction samples and placed in cocultures with mouse fibroblasts, NIH3T3 (3566 cells/cm^2^) in SF7 [1] media supplemented with 2% FBS on a collage coated plate for 7–10 days. From the non-cultured breast epithelial cells, a minimum of 5000 flow-sorted cells were plated in a 60-mm dish and from the pre-cultured breast epithelial cells, 50 flow-sorted cells (maximum 200 cells) were plated in per 60 mm dish. After 7–10 days (7 days for precultured and 8–10 days for non-cultured), the colonies were fixed with methanol: acetone (1:1 vol/vol) and stained with crystal violet and the colony numbers were recorded as described before [1, 3]. For some experiments, flow-sorted cells were placed in the CFC assays for 16–18 h first and then treated with either vehicle control (PBS) or 50 ng/mL of recombinant human Wnt7A or Wnt3A ligand (rhWnt7A, rhWnt3A). The Wnt ligand concentrations were optimized using the CFC assays (Additional file 4: Figure S3B).

### In vitro expansion of human breast epithelial progenitors

The subset “a”, subset “b” of the bipotent progenitors, and the LRPs were isolated from pre-cultured breast epithelial cells obtained from 3 different breast reduction samples and cultured as described before [19]. At every passage, expression of CD90 and NR3 was determined via flow cytometry. The clonogenic potential of the expanded progenitors were analyzed using the CFC assay.

### Re-plating of breast epithelial progenitors

The subset a, subset b of the bipotent progenitors, and the LRPs were isolated from the 4-day cultures of breast reduction samples. Fifty cells from each progenitor subset were placed in the CFC assays in triplicates to provide input progenitor frequencies. Immature and mature colonies observed on day 4 and day 7 respectively, were trypsinized and made into single cells as described and re-plated in serial CFC assays. After 7 days, the colony-forming ability of the re-plated cells was determined based on colony numbers and colony types. CD90 and NOTCH3 expression was also quantified in cells present in the immature and mature colonies via flow cytometry.

### Immunocytochemistry

The colonies from the CFC assays were fixed and permeabilized with chilled methanol: acetone (1:1 vol/vol), preblocked, and stained with specific primary antibodies (CK 8/18 (clone# 5D3 Abcam), CK 14 (clone# EPR17350, Abcam), CD90 (clone# 5E10, Biolegend), ERα (clone# HC-20, Santa Cruz biotechnology), β-catenin (clone 14, BD biosciences)). Protein expression was detected via fluorescent conjugated secondary antibody. The antibody-stained colonies were visualized and imaged using an inverted fluorescent microscope. To quantify ERα protein expression, progenitor subsets were flow-sorted from the 4-day cultures of breast reduction samples and air-dried onto glass slides and fixed with 100% methanol at −20 °C, preblocked and stained with ERα antibody (1:50 dilution). ERα expression was determined using PE-conjugated secondary antibody and DAPI was used to visualized cell nucleus using a fluorescent microscope.

### Transcriptome profiling

184-hTert cells were transduced with either empty vector (EV) control or N1ICD or N3ICD expressing lentivirus and after 48 h the transduced GFP^+^ cells were sorted via FACS and total RNA was extracted using Trizol® from three independent infections. The RNA samples were reverse transcribed, labeled, and hybridized onto Affymetrix Human 2.0 ST array GeneChip™ by the Robarts Research London Regional Genomics Centre. The Partek® Genomic Suite® software (version 6.6) was used to generate normalized data, and the log2 expression value for each transcript was obtained using the Robust Multi-array Average (RMA) algorithm [20]. The top 200 most variable probeset across the 9 samples were identified for further analysis using coefficient of variation (standard deviation divided by mean) for each probeset. The hierarchical clustering of the data was obtained using the Partek® Genomic Suite® based on the selected probesets. The differential gene expression analysis was performed for three comparisons (N1ICD vs Control, N3ICD vs Control, N3ICD vs N1ICD) using the LIMMA package (version 3.36.3) [21]. The adjusted *p* values or false discovery rate (FDR) was performed after multiple testing correction. A gene expression with adjusted *p* value ≤ 0.05 was considered significant. Compared to the controls and using 1.5-fold change in transcript expression with FDR < 0.05 as cut-off, the differentially up and downregulated gene lists were generated. The differentially regulated N1ICD and N3ICD gene list were compared to identify genes uniquely regulated by NR1 and NR3 signaling. We employed two criteria to obtain NR1 and NR3-unique target gene lists; genes that were differentially regulated in one data set (e.g., NR3) but unaltered in another (e.g., NR1) and genes upregulated in one data set (e.g., NR3) but downregulated in other data set (NR1).

Using the Ingenuity Pathway Analysis (IPA) algorithm, the canonical pathways enriched in N3ICD and N1ICD datasets was determined. Gene set enrichment analysis (GSEA) [22] was performed using a pre-ranked gene list (1.5-fold up and down, FDR < 0.05).

### Statistical analysis

The statistical analysis and graphs were generated using GraphPad Prism software (GraphPad Software Inc., version 6). Depending on the nature of the experiment and data obtained, an unpaired two-tailed *t* test or two-way anova was performed. An asterisk symbol was used to represent the level of significance, *p ≤ 0.05, **p ≤ 0.01, ***p ≤ 0.005, ****p ≤ 0.001.

## Results

### NOTCH3 expressing breast cells exhibit a basal-luminal phenotype

**Fig. 1:**
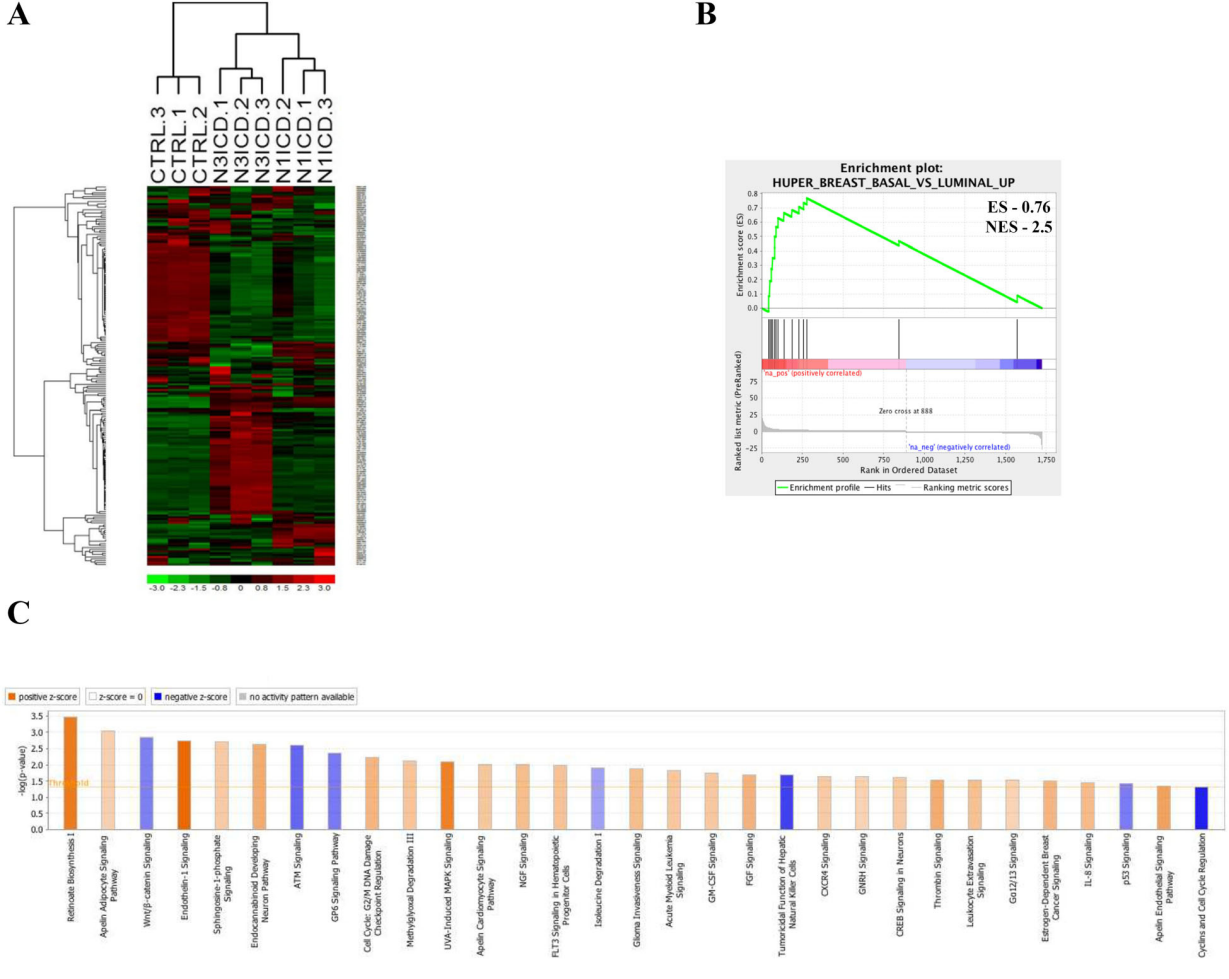
NOTCH3-overexpressing breast epithelial cells exhibit basal-luminal phenotype. **a** Hierarchical cluster analysis of microarray data is shown. **b** The gene set enrichment analysis of the NOTCH3 (NR3)-unique targets shows a basal-luminal gene signature. **c** Ingenuity pathway analysis of the microarray data shows enrichment of different canonical signaling pathways in NR3 unique data set

Given the involvement of NR3 in luminal cell fate, we hypothesized that genes uniquely regulated by NR3 are involved in the process of luminal cell lineage commitment. To this end, differential gene expression analysis was employed to identify the unique gene targets and the corresponding signaling pathways that are specifically regulated by NR3 in 184-hTert cells transduced to express a constitutively active form of NR3 (NR3 intercellular domain, N3ICD) and NR1 (N1ICD). The increased transcript levels of *NR1*, *NR3*, and *HES1*, a common target of all 4 Notch receptors, in the transduced cells were confirmed by real-time qPCR (Additional file 5: Figure S1A&B). Also, the increased *FZD7* expression, a previously identified NR3-unique target gene [15], in N3ICD-184-hTert cells and not in the N1ICD-184-hTert cells was confirmed by qPCR (Additional file 5: Figure S1C). Transcript expression of different genes in 3 separately transduced 184-hTert cells were analyzed using the Affymetrix Human 2.0 ST array GeneChip™. Hierarchical clustering analysis showed that the biological replicates clustered together, confirming the reproducibility of the gene expression data (Fig. 1a). By cross-comparing the NR1 and NR3 up and downregulated gene lists, we generated a sub-list of NR3 and NR1 unique gene targets (1731 and 873 genes accordingly, Additional file 6: Table S1 and Additional file 7: Table S2). The gene set enrichment analysis (GSEA) of the NR3-unique gene list revealed the presence of both basal (myoepithelial) and luminal cell markers (Fig. 1b). The Ingenuity Pathway Analysis (IPA) software revealed that the NR3-unique gene targets were significantly enriched for stem and progenitor cell proliferation and differentiation-related signaling such as the Ephrin B and Endothelin signaling and the Wnt signaling pathways (Fig. 1c). On the other hand, the NR1-unique gene targets showed significant enrichment for only two signaling pathways: the acute phase response and the Neuregulin signaling pathways (Additional file 5: Figure S1D). The differential expression of some of the NR3-unique targets was validated in N1ICD-, N2ICD-, N3ICD-, N4ICD-184-hTERT cells by qPCR where *Ephrin type-B receptor 3* (*EPHB3*), *GABA receptor ε (GABRE) and Regulator of G-protein signaling 2 (RGS2)* genes were confirmed to be specific and unique targets of NR3 (Additional file 5: Figure S1E). The preferential expression regulation of *GABRE* gene by NR3 was further validated at the protein level using flow cytometry (Additional file 5: Figure S1F&G).

### NOTCH3-FZD7 signaling restricts bipotent progenitors to the luminal cell fate

Since NR3 expression activates Wnt signaling in breast epithelial cells and that FZD7 is a unique gene target of NR3 [15], we therefore investigated the role of NR3-FZD7 signaling in luminal cell lineage determination in the human breast tissue. For this purpose, we employed gain and loss of gene function studies using primary human breast epithelial cells, obtained from breast reduction samples. For this purpose, lineage depleted (CD45^+^CD31^+^ removed, Lin^−^) breast epithelial cells were transduced with lentivirus expressing either short hairpin RNA (shRNA) to target *NR3* and *FZD7* transcripts or lentivirus expressing the NR3 intracellular domain (N3ICD) or full-length FZD7.

**Fig. 2:**
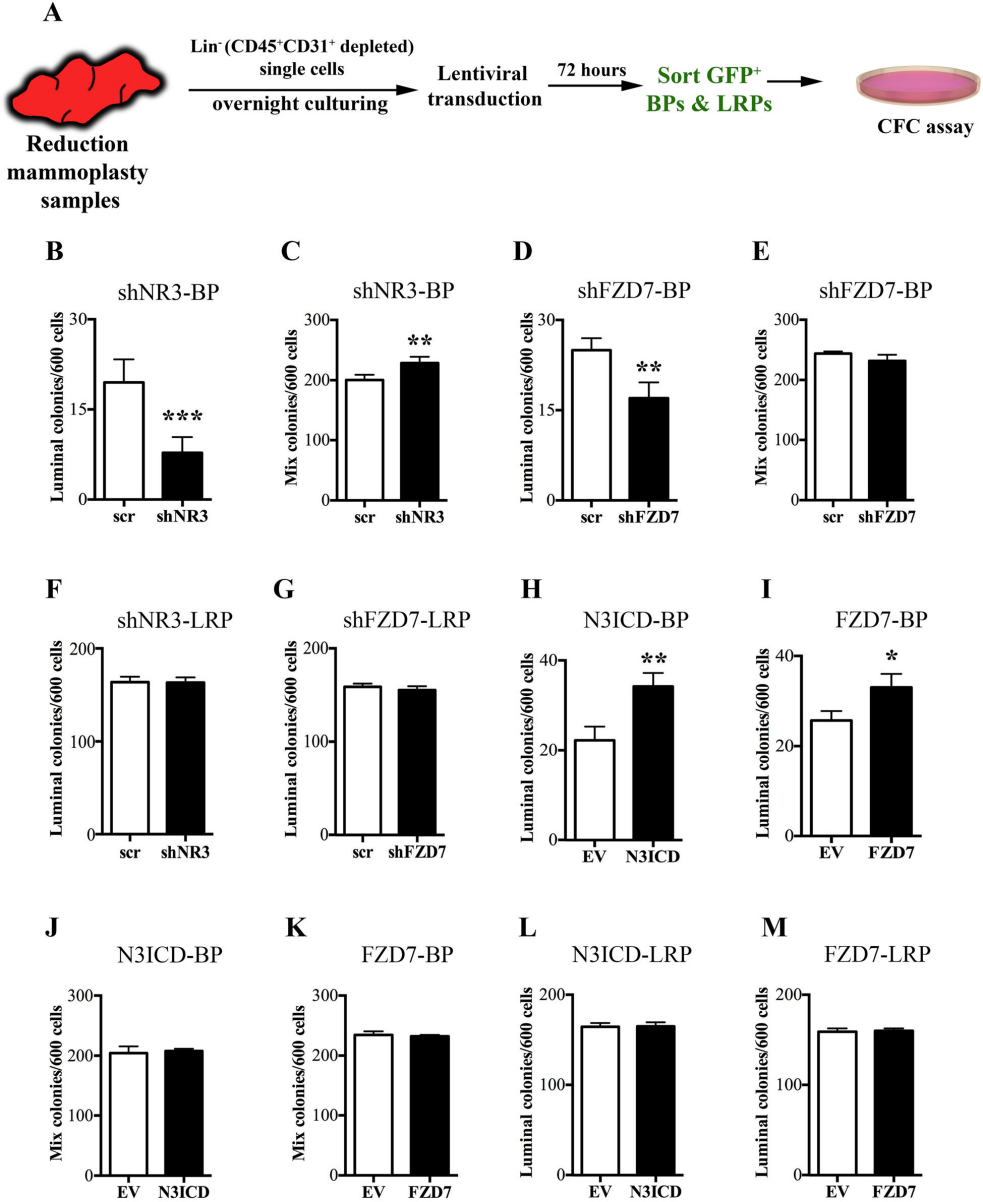
NOTCH3-FZD7 signaling is required for luminal cell fate commitment of bipotent progenitors. **a** Experimental strategy to apply gain and loss of NOTCH3 Receptor (NR3) and FZD7 function in the bipotent (BP, EpCAM^+^CD49f^+^CD90^+^) and luminal-restricted (LRP, EpCAM^+^CD49f^+^CD90^−^) progenitors isolated from transduced green fluorescent protein-positive (GFP^+^) precultured breast reduction samples. Average number of mix and pure luminal colonies generated from the BPs lacking either NR3 (shNR3-BP, **b**, **c**) or lacking FZD7 expression (shFZD7-BP, **d**, **e**), and from LRPs either lacking NR3 (shNR3-LRP, **f**) or lacking FZD7 (shFZD7-LRP, **g**) expression, or shScrambled control (scr), is shown as bar graphs. **h**, **i** Bar graphs showing total number of pure luminal colonies generated from BPs overexpressing either N3ICD (N3ICD-BP) or full-length FZD7 (FZD7-BP) or empty vector (EV), total number of mix colonies generated from N3ICD-BP or FZD7-BP (**j**, **k**), or from LRPs overexpressing N3ICD (N3ICD-LRP) or FZD7 (FZD7-LRP) (**l**, **m**). (*n* = 3, *p ≤ 0.05, **p ≤ 0.01, ***p ≤ 0.005)

The transduced, GFP^+^ BPs (EpCAM^+^CD49f^+^CD90^+^) and luminal-restricted progenitors (LRPs, EpCAM^+^CD49f^+^CD90^−^) were obtained by fluorescent activated cell sorting (FACS) and colony-forming cell (CFC) assay was performed to assess their differentiation potential (Fig. 2a). The knockdown and overexpression efficiencies for NR3 and FZD7 in the primary cells was validated by flow cytometry where the shRNA-transduced cells showed 82.3 ± 1.7% decrease in NR3 and 75.0 ± 2.8% decrease in FZD7 protein levels. The N3ICD- and FZD7-transduced primary cells showed 6.9 ± 0.9-fold increase in NR3 and 4.4 ± 0.7% fold increase in FZD7 levels (Additional file 8: Figure S2A-E). In addition, NR3-regulated FZD7 expression was confirmed in the shNR3 and N3ICD-transduced primary cells (Additional file 8: Figure S2F&G).

We observed that knocking down NR3 in the bipotent progenitors (BPs) significantly diminished (2.5 ± 1.3-fold decrease) the number of pure luminal colonies (Fig. 2b) while at the same time enhanced their mix colony-forming potential, although by a small but statically significant margin (Fig. 2c). Similar to NR3, decreased FZD7 expression in the bipotent progenitors also resulted in decreased pure luminal colonies (Fig. 2d, 1.5 ± 1.1-fold decrease). However, this decrease in the pure luminal colony numbers was not as striking as the loss of NR3 expression in the bipotent progenitors. The mix colony-forming potential of the shFZD7-Bipotent progenitors was unaffected (Fig. 2e). In contrast, the colony-forming potential of the luminal-restricted progenitors was independent of NR3 or FZD7 signaling (Fig. 2f, g).

The bipotent progenitors overexpressing N3ICD or FZD7 (Fig. 2h, i) generated more pure luminal colonies as compared to empty vector control-infected progenitors while the mix colony numbers remained unchanged in both the conditions (Fig. 2j, k). As observed with the loss of function studies, overexpression of N3ICD or FZD7 did not have any influence on luminal-restricted progenitors (Fig. 2l, m). Thus, NR3 and its unique gene target FZD7 play a critical role in restricting bipotent progenitors to luminal cell fate.

### Wnt7A-FZD7 signaling regulate pure luminal colony numbers in the bipotent progenitors

**Fig. 3:**
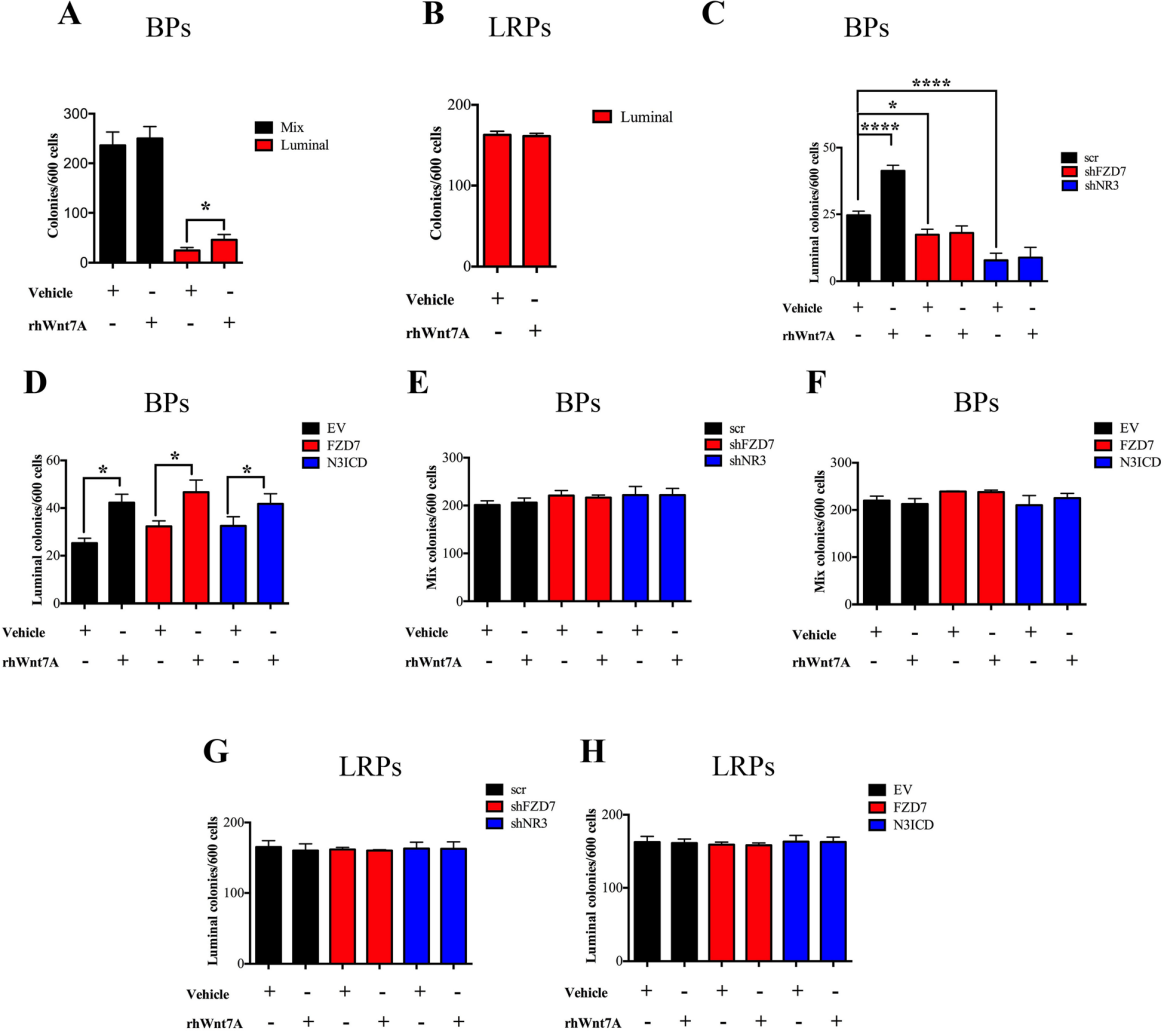
Wnt7A-induced differentiation of bipotent progenitors into luminal progenitors requires NOTCH3-FZD7 signaling. Bipotent progenitors (BPs) and the luminal-restricted progenitors (LRPs) were obtained from precultured breast reduction samples and placed in colony-forming assays treated with either vehicle control (Vehicle) or recombinant human Wnt7A (rhWnt7A). Average colony-forming cell (CFC) frequency ± SD in the BPs (**a**) and LRPs (**b**) are shown as bar graphs. **c**–**h** Transduced BPs and LRPs (shNR3, shFZD7, N3ICD, FZD7, scr, EV) as in Fig. 2 were obtained and placed in CFC assays with or without rhWnt7A. The average luminal and mix CFC frequency ± SD from the transduced BPs is shown as bar graphs (**c**–**f**). The average CFC frequency ± SD of luminal colonies from the transduced LRPs are shown as bar graphs (**g**, **h**). (*n* = 3, *p ≤ 0.05, ****p ≤ 0.001)

To investigate the effect of Wnt signaling on luminal cell fate, the bipotent and luminal-restricted progenitors were treated with a known canonical ligand Wnt3A or a known FZD7 ligand, Wnt7A which can activate both canonical and noncanonical Wnt signaling [23–30]. To this end, bipotent and luminal-restricted progenitors were obtained by FACS from breast reduction samples and placed in CFC assays with either vehicle control or recombinant human Wnt3A (rhWnt3A) or Wnt7A (rhWnt7A) (Additional file 4: Figure S3A). We used dose-response experiments first to optimize rhWnt7A and rhWnt3A concentrations (Additional file 4: Figure S3B-D). To avoid any potential effects of rhWnt3A and rhWnt7A on progenitor cell attachment, the recombinant Wnt ligands were added 16 h after the start of the CFC assays. We found that addition of rhWnt3A did not have any effect on colony-forming ability of the bipotent or the luminal-restricted progenitors. However, addition of rhWnt7a to the BPs increased the pure luminal colony numbers by 2.4 ± 0.2 folds, as compared to vehicle control (Fig. 3a). The mix colony formation potential of the BPs was not affected by rhWnt7A (Fig. 3a). In addition, rhWnt7A had no measurable effects on the colony-forming potential of the LRPs (Fig. 3b and Additional file 4: Figure S3D). Previously it was shown that secreted Wnt7A by breast tumor cells causes fibroblasts to assume an activated phenotype based on their expression of alpha smooth muscle actin (αSMA) [31]. To investigate if the addition of rhWnt7A to the CFC cultures causes the fibroblasts to assume an activated phenotype, fibroblasts-only cultures were treated with either vehicle control or rhWnt7A and expression of αSMA was examined in the fibroblasts. We found that rhWnt7A, at the least at a concentration that we used, did not result in αSMA expression (Additional file 1: Figure S4A-B).

Next, we assessed whether rhWnt7A-induced increase in pure luminal colony-forming potential of the bipotent progenitors requires FZD7. For this purpose, lentiviral transduction was used to knockdown *FZD7* expression or overexpress full-length FZD7 receptor in separately obtained BPs and LRPs that were then placed in CFC assays treated with either vehicle control or rhWnt7A. As expected, in the presence of rhWnt7A, the BPs’ potential to generate pure luminal colonies was significantly increased (Fig. 3c, scrambled control-bipotent progenitors). However, rhWnt7A failed to increase the luminal colony numbers in the BPs that show significantly reduced FZD7 expression (shFZD7 or shNR3-bipotent progenitors, Fig. 3c). Addition of rhWnt7A to BPs overexpressing FZD7 or N3ICD further increased their potential to generate pure luminal colonies (Fig. 3d) but it had no effects on the mix colony numbers (Fig. 3e, f). Gain and loss of NR3 or FZD7 function in the LRPs did not affect their colony-forming potential irrespective of rhWnt7A treatment (Fig. 3g, h).

### NOTCH3^+^FZD7^+^ cells represent a basal-luminal progenitor subtype

**Fig. 4:**
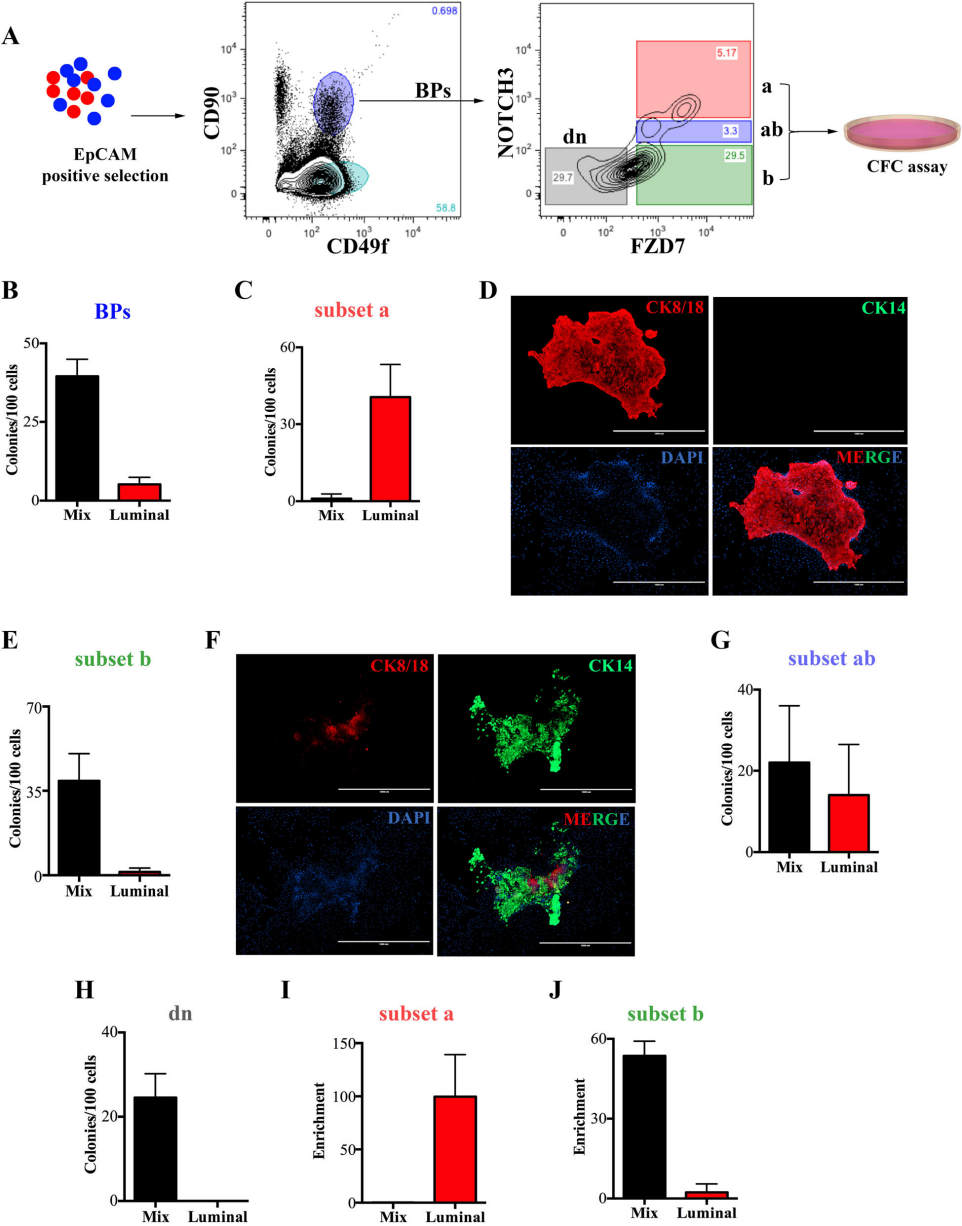
NOTCH3^+^FZD7^+^ cells represent a basal-like luminal progenitor. **a** Experimental strategy for isolating bipotent progenitors (BPs) and their subsets based on NOTCH3 and FZD7 receptors expression is shown; NR3^high^FZD7^+^ (subset a), NR3^−^FZD7^+^ (subset b), NR3^med^FZD7^+^ (subset ab), and the double negative NR3^−^FZD7^−^ (subset dn). **b** The average ± SD initial progenitor subtype frequency in the unseparated BPs are shown as bar graphs. Average ± SD frequency of colonies generated from subset “a” cells and representative immunofluorescent staining of the colonies for CD8/18 and CD14 (**c**, **d**), for subset “b” (**e**, **f**) and subset “ab” and “dn” (**g**, **h**) are shown. The lines represent 1000 μm, and DAPI staining is used to visualize the nucleus. **i**, **j** Input progenitor numbers present in the unseparated bipotent progenitors (**b**) is used to calculate the enrichment of each progenitor in the subsets “a” and “b”

Our data so far indicate that the luminal progenitors present in the BPs-enriched human breast epithelial cells are different than the LRPs in that they are NR3 and FZD7-responsive. We, therefore, hypothesized that the NOTCH3^+^FZD7^+^ cells represent a transitional cell state between the BPs and LRPs. To test this hypothesis, the colony-forming potential of the EpCAM^+^CD49f^+^ CD90^+^ NR3^high^ FZD7^+^ (subset a), the EpCAM^+^CD49f^+^ CD90^+^ NR3^low/−^ FZD7^+^ (subset b), the EpCAM^+^ CD49f^+^ CD90^+^ NR3^medium^ FZD7^+^ (subset ab) and the EpCAM^+^ CD49f^+^ CD90^+^ NR3^−^ FZD7^−^ (double negative, dn) subset of BPs isolated from primary human breast epithelial cells was determined via the CFC assay (Fig. 4a). The unseparated bulk BP-enriched fraction (EpCAM^+^CD49f^+^CD90^+^) gave rise to 39.5 ± 5.4% mix and 5.2 ± 2.2% pure luminal colonies (Fig. 4b). As expected, we found that the NR3^high^ BPs (subset a) cells were FZD7 positive [15]. Very interestingly, progenitors enriched in “subset a” generated only pure luminal colonies at a frequency of 33.3 ± 13.6% (Fig. 4c). All colonies developed from “subset a” were CK8/18 positive with no detectable CK14 expression (Fig. 4d) suggesting that “subset a” represents basal-like luminal progenitors (BLPs). On the other hand, the NR3^low/−^ subset of BPs (subset b) only generated mix colonies with cytokeratin14 and cytokeratin 8/18 positive colonies (Fig. 4e, f). The NR3^medium^ BPs (subset ab) give rise to both pure luminal and mix colony types (Fig. 4g), while the NR3^−^FZD7^−^ (dn) subset of BPs produced only mix colonies (Fig. 4h). Notably, the majority of the pure luminal colony-forming cells present in the bulk bipotent progenitors were selectively and specifically enriched in the BLP subpopulation (99.6% ± 39.6, Fig. 4i) while the mix colony-forming cells were enriched in the NR3^−^, non-BLP cells (53.6% ± 5.4) (Fig. 4j).

To determine if BLPs are present in the non-cultured breast reduction samples, the bipotent progenitor-enriched (EpCAM^low/−^CD49f^bright^) fraction [32, 33] of human breast epithelial cells were obtained from the freshly dissociated reduction mammoplasties and were further subfractionated based on the expression of BLP markers; NR3, CD90, and FZD7. Similar to pre-cultured cells, majority of the NR3^+^ cells also expressed FZD7 in the non-cultures BPs (ncBPs) and therefore, FZD7 was not pursued further to identify the BLP subset in the non-cultured cells (Additional file 9: Figure S5B&C). CD90 was strongly expressed in majority of ncBPs (78.9% ± 4.6, Additional file 9: Figure S5D). Although, only 1.2% ± 0.2 of ncBPs express NR3, it was strongly expressed in the EpCAM^low^ ncBPs as compared to the EpCAM^−^ ncBPs (Additional file 9: Figure S5E). Next, bulk ncBPs and their CD90^+^NR3^+^ (subset a), and CD90^+^NR3^−^ (subset b) subsets were flow-sorted and placed in CFC assays to assess their colony-forming potential (Additional file 10: Figure S6A). As expected [1, 3], the bulk ncBPs generated mix colonies at a higher frequency (1.2 ± 0.1) than the pure luminal colonies (0.03 ± 0.01) (Additional file 10: Figure S6B). While the mix colony-forming cells (pure bipotent progenitors) were more enriched in the CD90^+^NR3^−^ subset of ncBPs, the pure luminal colony-forming cells (or the BLPs) were enriched in the CD90^+^NR3^+^ subset of ncBPs (Additional file 10: Figure S6C-D). These observations indicate that BLPs can be separately obtained from the non-cultured bipotent progenitors, although, they are present at very low frequencies.

### BLPs represent a more primitive population of luminal progenitors compared to LRPs

**Fig. 5:**
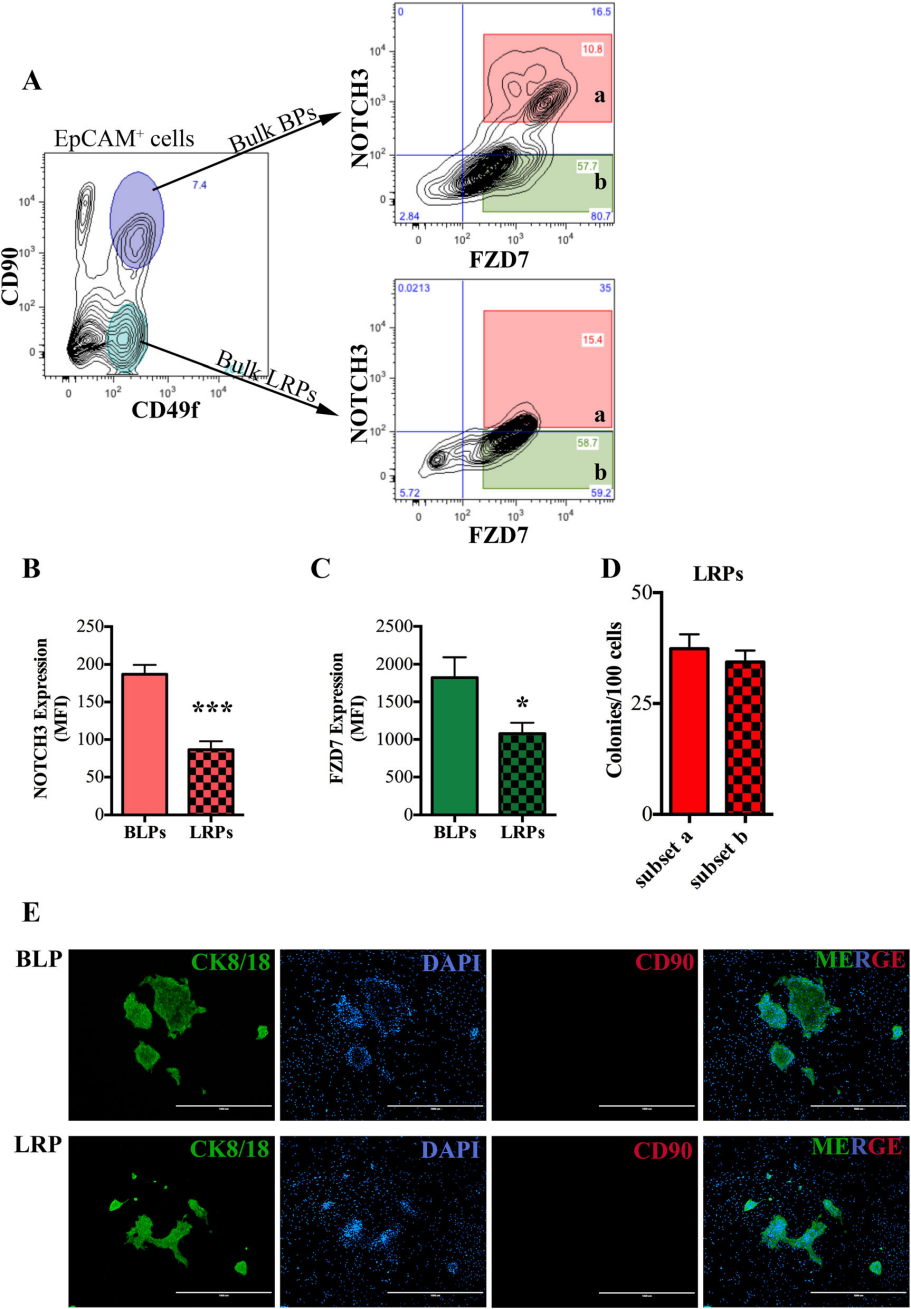
BLPs lose CD90 expression during differentiation. **a** Cell sorting strategy to isolate the basal-like luminal progenitors (BLPs, NOTCH3^+^FZD7^+^) and the NOTCH3^+^FZD7^+^ subset of the luminal-restricted progenitors (LRPs) from precultured breast reduction samples that are placed in colony assays. Bar graphs show the average ± SD mean fluorescence intensity of NOTCH3 (**b**) and FZD7 (**c**) expression in BLPs and LRPs. **d** Bar graphs show the average ± SD progenitor frequency in subsets “a” and “b” of LRPs. **e** Representative immunofluorescent staining of colonies generated from BLPs and LRP-subset a for cytokeratin 8/18 (green), DAPI (blue), and CD90 (red). The lines represent 1000 μm. All data obtained from 3 independent breast reduction samples. Additional file 9: Figure S5A shows mix colonies stain positive for CD90 expression (***p ≤ 0.005)

To further characterize the differentiated progeny of the different progenitor subsets, we flow-sorted pure BPs (EpCAM^+^CD49f^+^CD90^+^NR3^−^FZD7^+^, subset b), BLPs (EpCAM^+^CD49f^+^CD90^+^NR3^high^FZD7^+^), bulk LRPs (EpCAM^+^CD49f^+^CD90^−^), and two subsets of LRPs (NR3^high^FZD7^+^ and NR3^low^FZD7^+^) from precultured primary human breast epithelial cells and placed them in CFC assays (Fig. 5a). Compared to LRPs, the NR3 and FZD7 expression was higher in the BLPs (Fig. 5b, c). Also, the colony-forming cell frequency of the NR3^+^FZD7^+^ and the NR3^−^FZD7^+^ subset of LRPs was similar, suggesting that NR3 and FZD7 expression does not offer further enrichment for LRPs (Fig. 5d). The colonies generated from each progenitor subtype were then assessed for CD90 expression. As expected, the mix colonies generated from BP-subset b progenitors stained positive for CD90 (Additional file 9: Figure S5A). In contrast to the LRPs, the CD90^+^ BLPs generated colonies consisting of CD90^−^ cells (Fig. 5e).

**Fig. 6:**
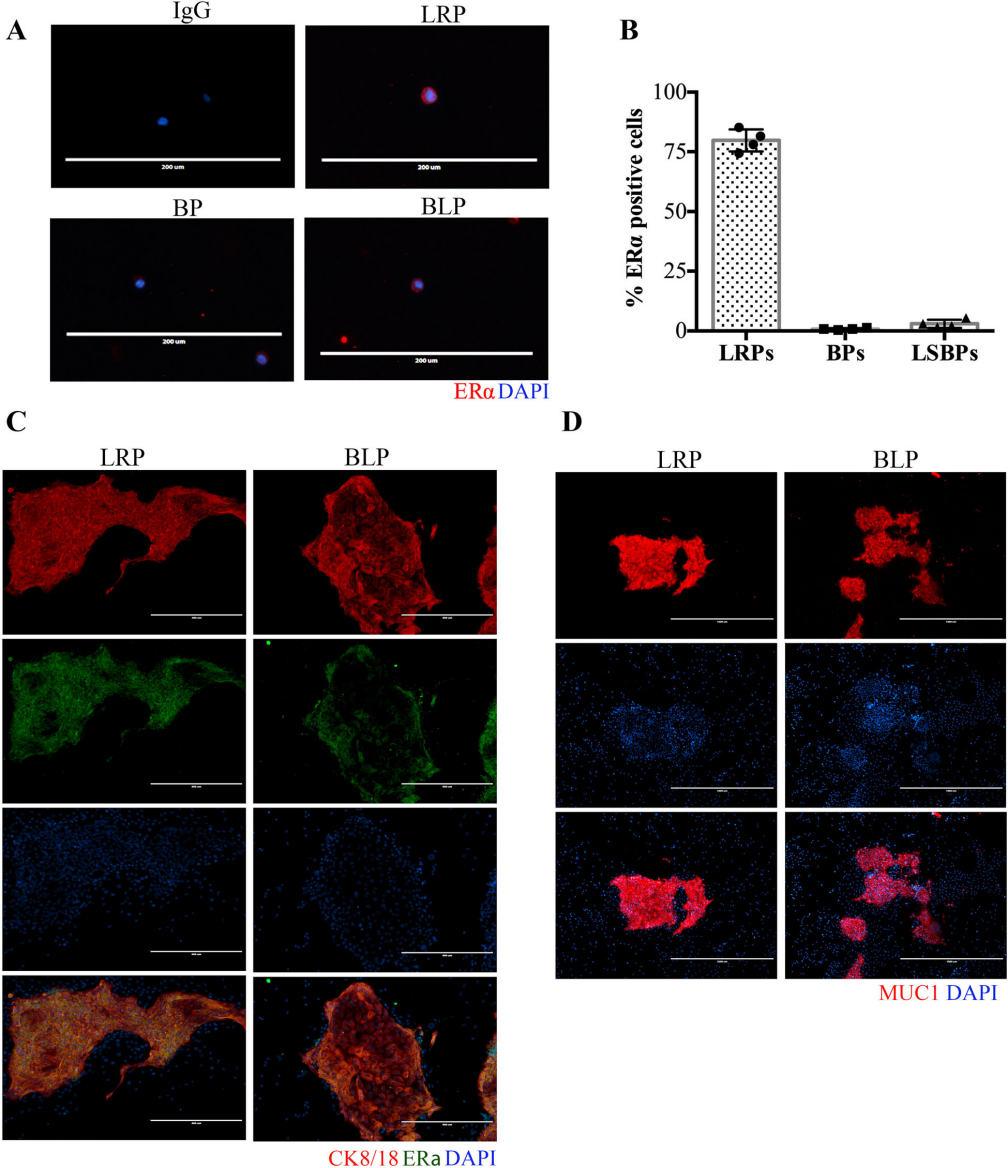
BLPs differ from LRPs in their ERα and MUC1 expression. **a** The bipotent progenitors (BPs), basal-like luminal progenitors (BLPs), and the luminal-restricted progenitors (LRPs) were obtained from precultured breast reduction samples, fixed on glass slides, and stained with fluorescent antibodies to detect estrogen receptor alpha (ERα) expression. Representative images of ERα expression in the different progenitor subsets are shown (the line represent 200 μm) and the average frequency ± SD of ERα^+^ cells in each progenitor subset from 4 independent breast reduction samples are shown in bar graphs (**b**). LRPs and BLPs were placed in colony assay and the colonies were stained with (**c**) CK8/18 (red), ERα (green), and DAPI (blue) (**c**) or (**d**) MUC1. The line represents 400 μm

The different progenitor subsets were further characterized with respect to the estrogen receptor alpha (ERα) expression. As expected, majority (79.7% ± 4.6) of LRPs stained positive for ERα whereas its expression was nearly undetectable in BPs (Fig. 6a, b). Although, only 2.9% ± 1.6 of BLPs stained positive for ERα (Fig. 6b), the pure luminal colonies generated from these BLPs consisted mostly of ERα positive cells (Fig. 6c). Furthermore, the luminal colonies generated from the MUC1^−^ BLPs contained MUC1^+^ cells (Fig. 6d). The bipotent progenitors do not express MUC1, while the LRPs and the luminal colonies generated from them are MUC1^+^ [1] (Additional file 4: Figure S3A). Taken together, these observations suggest that BLPs could represent an earlier less differentiated population of luminal progenitors compared to the LRPs.

### BLPs and LRPs are two distinct population of progenitors

**Fig. 7:**
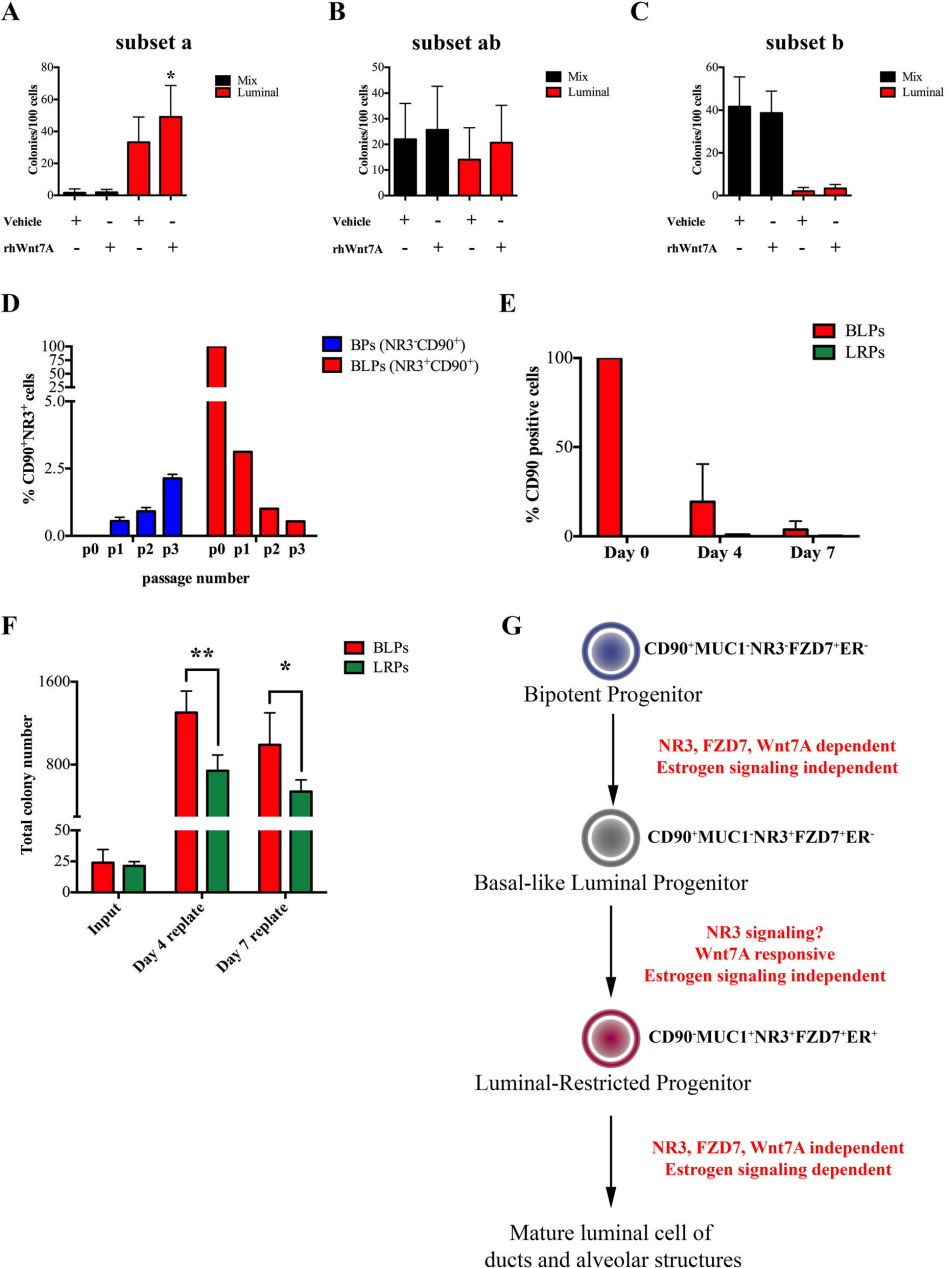
Wnt7A-responsive BLPs differentiate to generate LRPs. The basal-like luminal progenitors (BLPs or subset “a”), subset “ab” and subset “b” were isolates as in Fig. 4 and placed in colony assays treated with rhWnt7A. **a**–**c** The average ± SD frequency of luminal and mix colonies from each progenitor subtype is shown in bar graphs. **d** The NR3^−^CD90^+^ BPs and BLPs were obtained from 3 reduction samples, cultured in vitro, and percentage of NR3^+^CD90^+^ cells was determined by flow cytometry at each passage (P). In the case of BLPs, only 1 sample out of 3 successfully expanded. **e** BLPs and luminal-restricted progenitors (LRPs) were cultured in colony assays and frequency of CD90^+^ cells after 4 and 7 days (immature and mature colonies respectively) was determined by flow cytometry. **f** The immature (d4) and mature (d7) colonies were made into single-cells and placed in serial colony assays and the total colony numbers are depicted in the bar graphs. **g** Schematic showing the developmental process of bipotent progenitors (BPs) differentiation into luminal-restricted progenitors (LRPs). The bar graphs show the average values ±SD for 3 reduction samples. (*p ≤ 0.05; **p ≤ 0.01)

We observed that Wnt7A-FZD7 signaling significantly increased the number of pure luminal colonies when bipotent progenitors were placed in CFC assays. To examine if this increase in the luminal colonies is due to BLPs’ response to Wnt7A, subset “a” (BLPs, NR3^+^FZD7^+^), subset “ab” and the subset “b” of the bipotent progenitors (as described in Fig. 4a) were obtained from precultured breast reduction samples and placed in rhWnt7A-treated CFC assays. rhWnt7A significantly increase the luminal colony numbers generated from the BLPs (Fig. 7a) and had no significant effect on the subsets “ab” and “b” (Fig. 7b, c). This data in combination with our previous finding that LRPs are not responsive to Wnt7A signaling (Fig. 3b), provides firm evidence that BLPs and LRPs are two distinct, non-overlapping population of progenitors.

### NOTCH3^−^ bipotent progenitors generate BLPs that differentiates into LRPs

So far, our data indicate that BPs can be further separated into 3 subsets based on NR3 and FZD7 expression, where the NR3-medium expressing “ab” subset of BPs contained both bipotent and the BLPs (Fig. 5g) and the NR3^high^, subset a, contained pure BLPs. In order to investigate if cells in the NR3^−^, subset “b” of bipotent progenitors, differentiate to generate BLPs, we separately obtained subsets “a” (BLPs) and “b” from BPs and placed them in long-term in vitro cultures conditions that support the maintenance and expansion of human breast epithelial progenitors [20]. These cultures were carried for 3 passages, and expression of NR3 and CD90, the BLP signature, was examined at every passage. Although initially absent, a small number of NR3^+^CD90^+^ cells were detectable at the first passage, and their number continued to increase at the subsequent passages (Fig. 7d). On the contrary, using same culture conditions, the BLPs (subset “a”) failed to maintain their undifferentiated phenotype and lost expression of NR3 and CD90 (Fig. 7d) suggesting that the in the culture conditions used in this study, the NR3^+^CD90^+^ progenitor would have to continuously generated as they are not maintainable in such growth condition on their own.

In order to investigate the differentiation potential of BLPs to LRPs, we sorted BLPs and LRPs and placed them in CFC assay conditions and luminal cells present in the immature colonies (on day 4) and in the mature colonies (on day 7) were examined for CD90 expression and their colony-forming potential was evaluated by replating them into serial CFC assays. We observed a significant decrease in CD90 expression upon BLPs’ differentiation to generate immature and subsequently mature colonies (Fig. 7e). The immature and mature colonies generated by the BLPs contained pure luminal colony-forming cells and their frequency was not affected by the decrease in CD90 expression (Fig. 7e). Although the immature and mature colonies generated by the LRPs also contained luminal colony-forming cells, their frequency was significantly lower than the BLPs. These data then suggest that the BLPs differentiate to produce cells with LRP phenotype and differentiation potential but with higher progenitor activity.

## Discussion

It was previously demonstrated that NR3 signaling was important for luminal cell lineage [1]. However, the molecular mechanisms underpinning NR3 signaling as well as the different progenitor subsets that are regulated by NR3 signaling remain unknown. In this study, we explored the signaling mechanisms that regulate the differentiation of the bipotent progenitors to the luminal-restricted progenitors. We report the existence of a new population of NR3^+^FZD7^+^CD90^+^ basal-like luminal progenitors that originate from the NR3^−^CD90^+^ bipotent progenitors. We further show that these basal-like luminal progenitors (BLPs) generate pure luminal colonies based on NR3 and FZD7-Wnt7A signaling which makes them distinct from the previously identified luminal-restricted progenitors (LRPs). Our data then indicate that the bipotent progenitor-enriched fraction of human breast epithelial cells represents a heterogeneous population of progenitors that can be further subdivided based on NR3 expression. In this case, the NR3^−^ subset represents pure bipotent progenitors, while the NR3^high^ subset represents the BLPs, and the NR3^medium^ cells represented a transitional state between the bipotent and luminal-restricted differentiation potentials. Our gene expression profiling data indicates that activation of NR3 signaling results in the differential expression of both basal and luminal cell markers. Taken together these data suggest the NR3 expression in bipotent progenitors could result in a basal-like luminal phenotype and eventually restriction to the luminal cell fate. Knocking down *NR3* in the bipotent progenitors enhances their potential to generate mix colonies while significantly decreasing the number of pure luminal colonies, suggesting that the bipotent progenitors by default generate mix colonies and the decision to differentiate into BLPs requires NR3 signaling. We found that increased expression of the NR3-specific gene target, FZD7, on its own is sufficient to increase the BLP numbers although to a lesser extent than NR3, suggesting that differentiation of NR3^−^FZD7^+^ subset of BPs into BLP could be initiated by activation of *NR3* expression resulting in increased *FZD7* expression.

A previous study suggested that *Nr3* expression could be controlled by the Pygo2-β-catenin complex in the mouse mammary stem cells (MaSCs) in that loss of Pygo2 in MaSCs resulted in increased Nr3 signaling and luminal fate specification [34]. It is interesting that the transcriptome profiling of different human breast epithelial progenitor subsets suggested that *PYGO2* expression is higher in the bipotent progenitors compared to the LRPs [1] indicating that, decreased *PYGO2* expression could also activate transcription of *NR3* in the bipotent progenitors.

Although we were able to maintain and expand BPs and LRPs in vitro, maintaining BLP phenotype in the growth conditions we used, proved to be difficult as these cells assumed the LRPs’ phenotype after 3 passages, but they showed significantly higher luminal colony-forming potential in these cultures than the LRPs. It is conceivable that the rapid turnover of these BLPs is due to the culture conditions used. Previously, LRPs were found to be CD90^−^MUC1^+^ER^+^ [1, 3] (Additional file 4: Figure S3A). Recent studies of mouse mammary gland have suggested that the luminal cell compartment in mouse mammary gland is heterogenous in nature consisting of highly proliferative ER^−^ luminal progenitors, low proliferative ER^+^ luminal progenitors and the mature luminal cells [35]. Such studies suggested that the ER^−^ luminal progenitors generate the ER- alveolar cells while the ER+ luminal progenitors generate the ER^+/−^ luminal cells of the ducts. In this study, we demonstrated that the CD90^+^MUC1^−^ER^−^ BLPs differentiate to generate CD90^−^MUC1^+^ER^+^ cells that retain their colony-forming ability. In such in vitro assays, epithelial to mesenchymal transition (EMT) is concern in that it might lead to the generation of artificial cell phenotypes. However, we report that the ER^−^ BLPs differentiate to generate ER expressing LRPs that are maintainable for 3 passages in our colony assay system, suggesting that EMT may not be an important consideration. Furthermore, we report the presence of BLPs in the non-cultures primary human breast epithelial cells. Together, these observations suggest that BLPs are a more primitive progenitor subtype than the LRPs. Our data then indicate that BLPs represent the pre-LRP progenitor population.

Using lineage-tracking, a recent report identified high Nr3 expressing cells to be quiescent luminal progenitors in the mouse mammary gland [36]. It is interesting that BLPs show significantly higher expression of NR3 compared to the more differentiated LRPs, suggesting that BLPs might be the human equivalent of the mouse Nr3^high^ luminal progenitors. In this regard, the transcriptome profiling of the two primitive luminal progenitors would help their further characterization.

The loss of *FZD7* in the bipotent progenitors resulted in a decrease in BLP numbers. However, this decrease was not as dramatic as *NR3* loss in the BPs, suggesting that in addition to *FZD7* other *NR3*-specific gene targets might play important roles in the commitment of bipotent progenitors to BLPs. Never the less, role of Wnt7A, a FZD7 ligand, in regulating the proliferation and differentiation of the primitive muscle stem cells has been shown before [27, 30]. In this study we show that Wnt7A enhanced the commitment of the NR3^−^FZD7^+^ bipotent progenitors to BLPs in a FZD7-dependent manner.

Although decreased *NR3* expression significantly reduced the BLP numbers, yet the shNR3-BPs generated mix colonies consisting of both luminal and the myoepithelial cells, suggesting that the production of luminal cells in the mix colonies is independent of NR3 signaling. This observation indicates that the restriction of bipotent progenitors to the luminal cell only fate is regulated by NR3 signaling. It remains to be seen if the NR3^+^ BLPs generate alveolar luminal cells compared to the ductal luminal cells. Interestingly, recent single-cell transcriptome analysis of human breast epithelial cells confirmed the previous notion that LRPs could be a progeny of the bipotent progenitors [6]. In this study, we demonstrate that the cooperation between two evolutionarily conserved signaling pathways, the Notch and Wnt, promote the differentiation of the bipotent progenitors to luminal-restricted progenitors via a basal-luminal progenitor subtype, the BLPs, and in this way our study has further expanded the hierarchical organization of the human breast epithelial cells. Although the origin of primitive undifferentiated ER^−^ and ER^+^ luminal progenitors in the mouse mammary gland remains elusive, here we show that the NR3^+^FZD^+^CD90^+^ER^−^ progeny of bipotential progenitors generate the ER^+^ luminal progenitors in human breast tissue.

## Conclusion

This study demonstrates that NR3-FZD7 signaling is required for differentiation of the bipotent progenitors into a new population of basal-like luminal progenitors. These high NR3-FZD7 expressing progenitor subtype lack ERα expression; however, they are capable of differentiating into ERα expressing luminal cells. These results redefine the lineage hierarchy of breast epithelial cells within the human breast epithelial cells. Based on these findings, it is inviting to hypothesize that the ERα^+^ breast cancer tumors might contain a similar ERα^−^ basal-like luminal progenitors that maintain the bulk of differentiated ERα^+^ tumor cells but themselves are refractory to anti-estrogen therapies and, therefore, ultimately contribute to the development of therapy resistance and tumor relapse.

## Additional files

Additional file 1: **Figure S4.** Wnt7A does not create an activated phenotype in fibroblasts. (JPG 1556 kb)

Additional file 2: **Table S3.** qPCR primers. (PDF 52 kb)

Additional file 3: **Figure S7.** Validation of FZD7 overexpressing construct in 184-hTert cells. (JPG 210 kb)

Additional file 4: **Figure S3.** Wnt7A and not Wnt3A commits bipotent progenitors to luminal cell fate. (JPG 1541 kb)

Additional file 5: **Figure S1.** Identification of NOTCH3-specific targets. (JPG 2084 kb)

Additional file 6: **Table S1.** List of genes uniquely regulated by NR3. (PDF 239 kb)

Additional file 7: **Table S2.** List of genes uniquely regulated by NR1. (PDF 139 kb)

Additional file 8: **Figure S2.** Successful knockdown and overexpression of NOTCH3 and FZD7 in primary human breast epithelial cells. (JPG 1056 kb)

Additional file 9: **Figure S5.** Non-cultured bipotent progenitors can be further subdivided based on NOTCH3 expression. (JPG 1653 kb)

Additional file 10 : **Figure S6.** BLPs are detectable in non-cultured primary breast epithelial cells (JPG 1113 kb)

## Data Availability

The data will be made available from the corresponding author based on reasonable request.

## Abbreviations

BLP: Basal-like luminal progenitor
CFC: Colony-forming cell
DAPI: 4′,6-Diamidino-2-phenylindole
DMEM: Dulbecco’s modified essential medium
EpCAM: Epithelial cell adhesion molecule
ERα: Estrogen receptor alpha
FACS: Fluorescent activated cell sorting
FBS: Fetal bovine serum
FITC: Fluorescein isothiocyanate
FZD7: FRIZZLED7
GAPDH: Glyceraldehyde-3-phosphate dehydrogenase
GFP: Green fluorescent protein
HBSS: Hanks balanced salt solution
IPA: Ingenuity pathway analysis
LIMMA: Linear model for microarray data HES1 Hairy and enhancer of split-1
LRP: Luminal-restricted progenitor
MFI: Median fluorescent intensity
MUC1: Mucin 1
NICD: Human notch intracellular domain
NR: Human notch receptor
PCR: Polymerase chain reaction
PE: Phycoerythrin
qPCR: Quantitative real-time PCR
RMA: Robust multi-array average
SF7: Serum free 7
shRNA: Short hair-pin RNA
THY1: Thymus cell antigen 1
WNT: Wingless-type MMTV integration site family
