# Supplementary figures and images for: Isolation and characterization of a new basal-like luminal progenitor in human breast tissue
Source: Stem Cell Res Ther. 2019 Aug 23;10:269. doi: 10.1186/s13287-019-1361-3 (PMC6708178; doi:10.1186/s13287-019-1361-3)

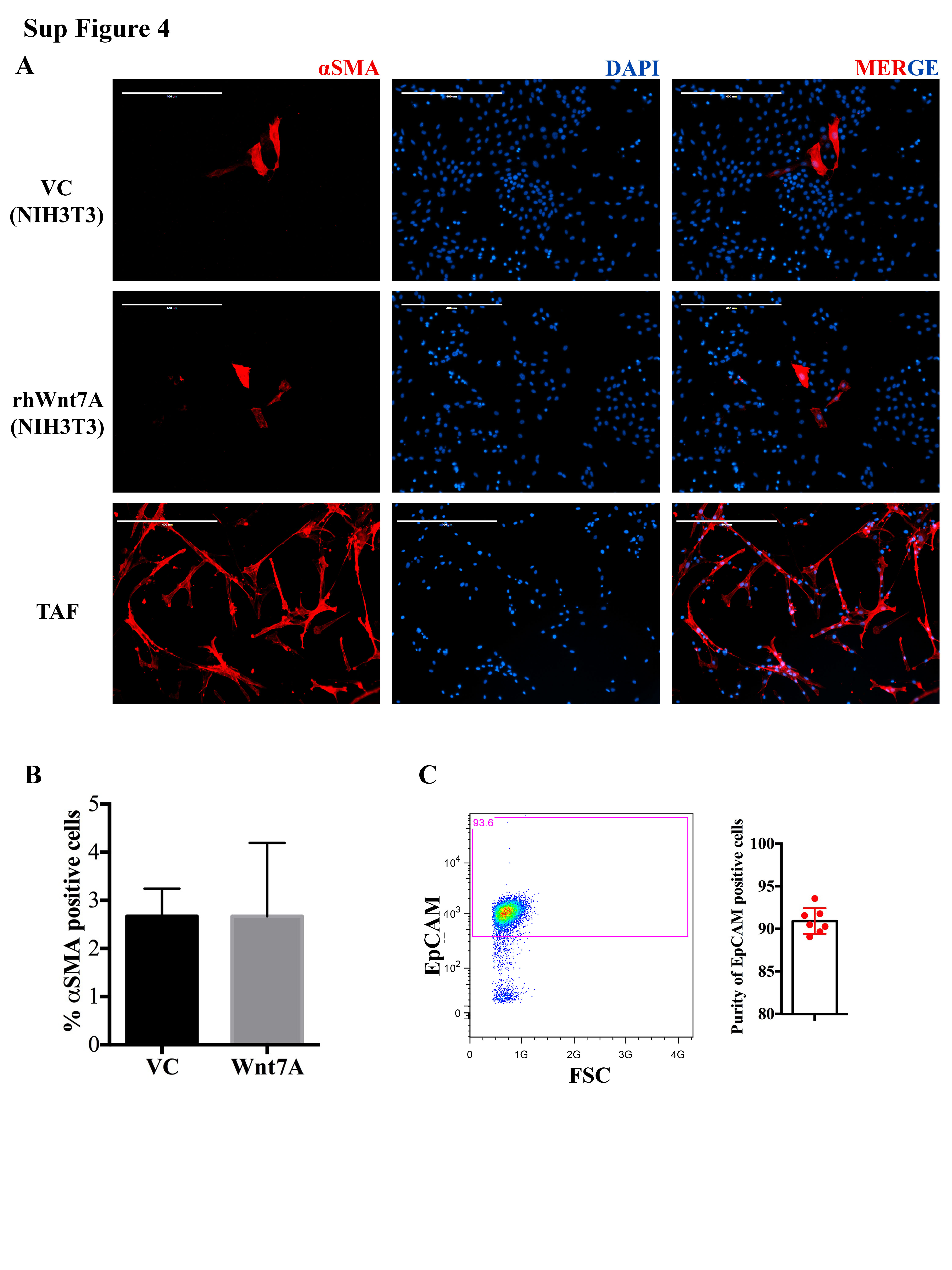

Supplement: Supplementary file 1 — Figure S4. Wnt7A does not create an activated phenotype in fibroblasts. (JPG 1556 kb) [file 13287_2019_1361_MOESM1_ESM.jpg]

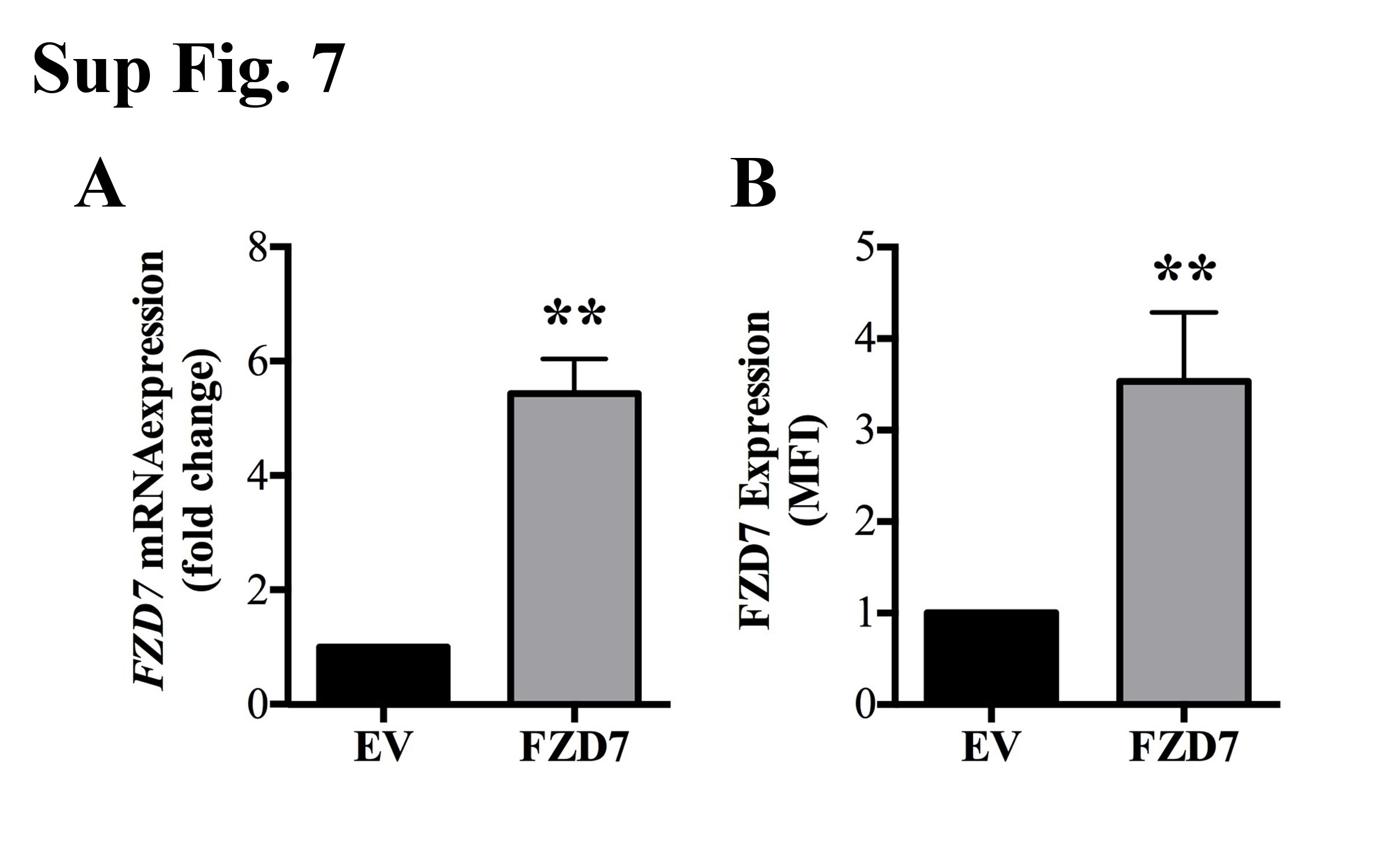

Supplement: Supplementary file 3 — Figure S7. Validation of FZD7 overexpressing construct in 184-hTert cells. (JPG 210 kb) [file 13287_2019_1361_MOESM3_ESM.jpg]

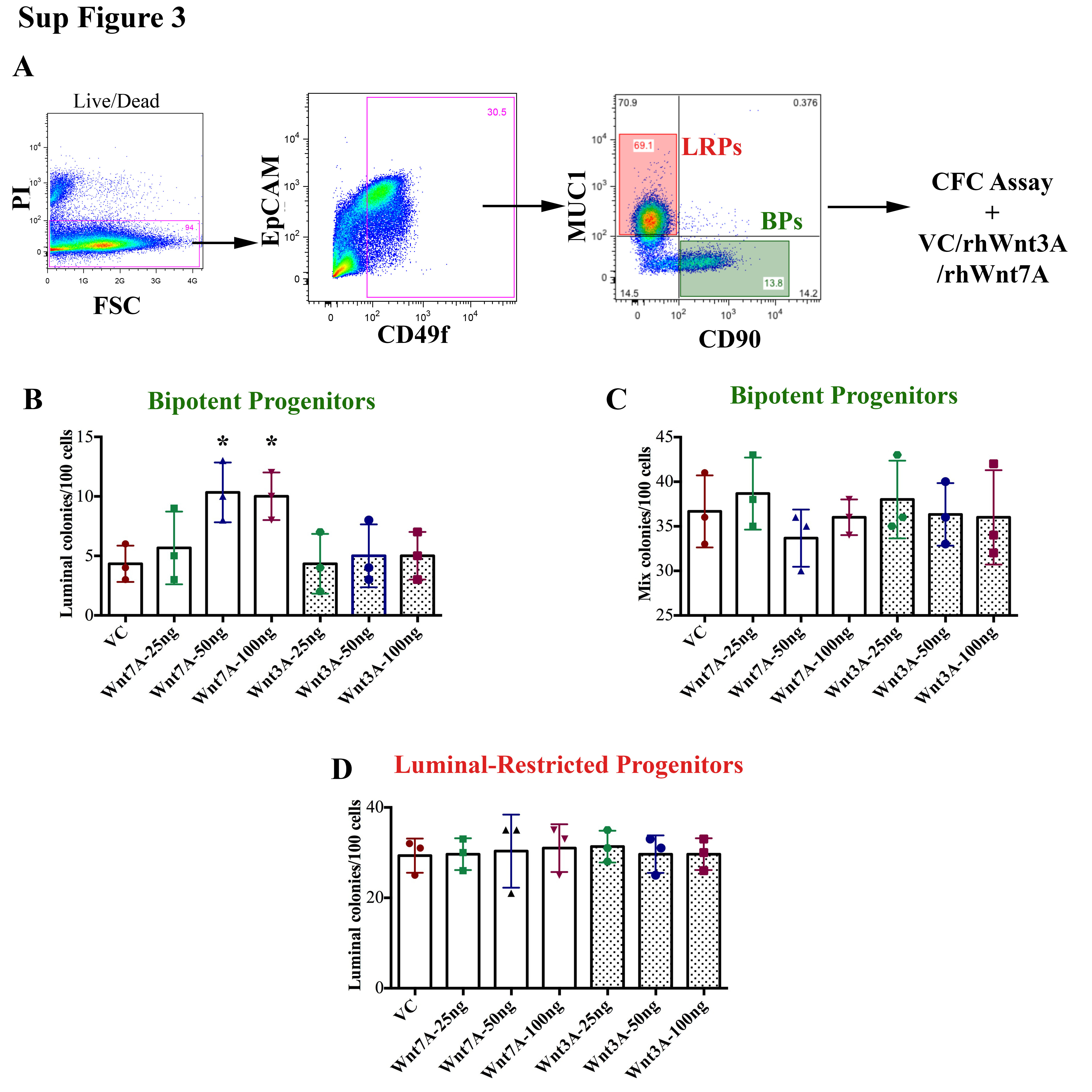

Supplement: Supplementary file 4 — Figure S3. Wnt7A and not Wnt3A commits bipotent progenitors to luminal cell fate. (JPG 1541 kb) [file 13287_2019_1361_MOESM4_ESM.jpg]

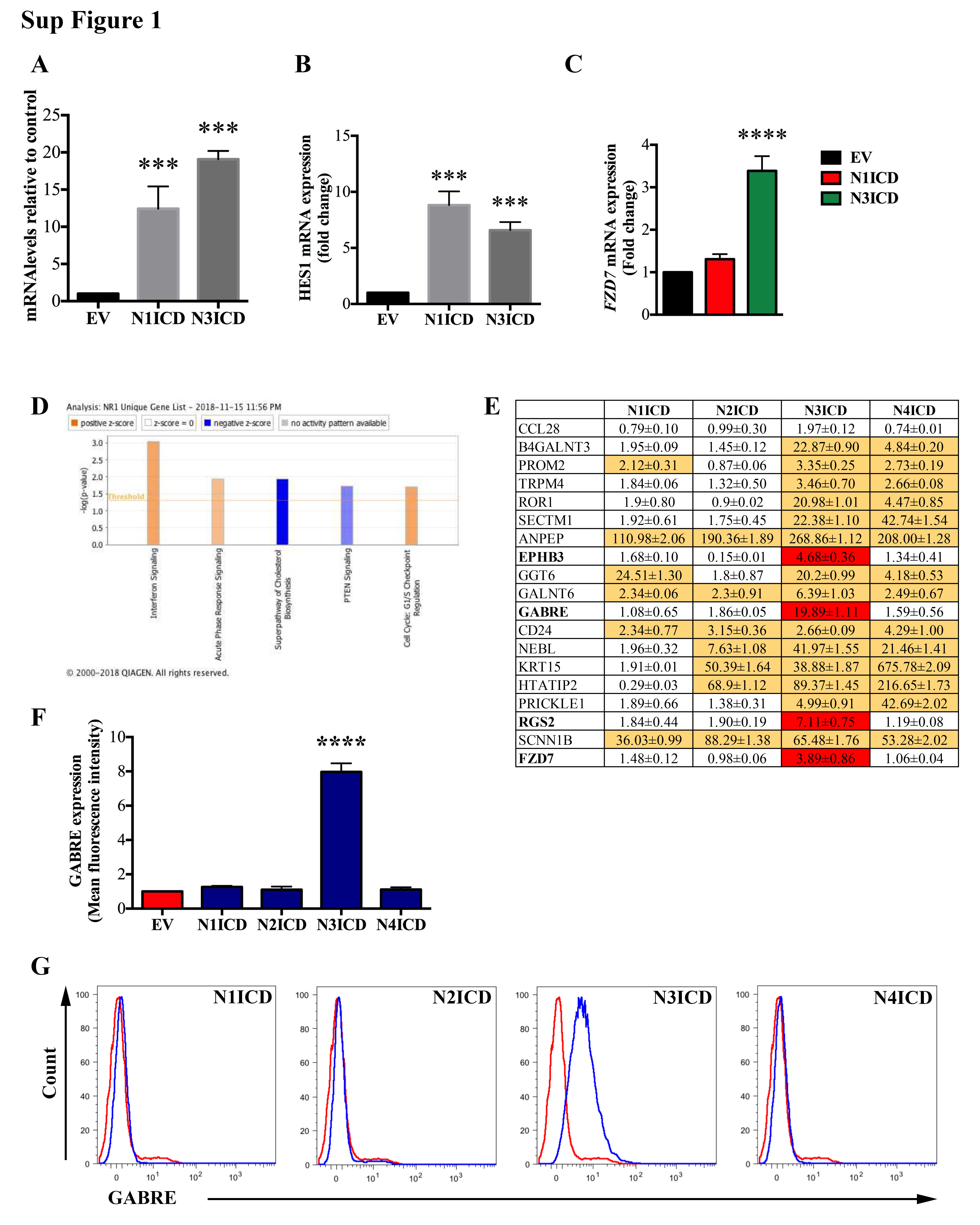

Supplement: Supplementary file 5 — Figure S1. Identification of NOTCH3-specific targets. (JPG 2084 kb) [file 13287_2019_1361_MOESM5_ESM.jpg]

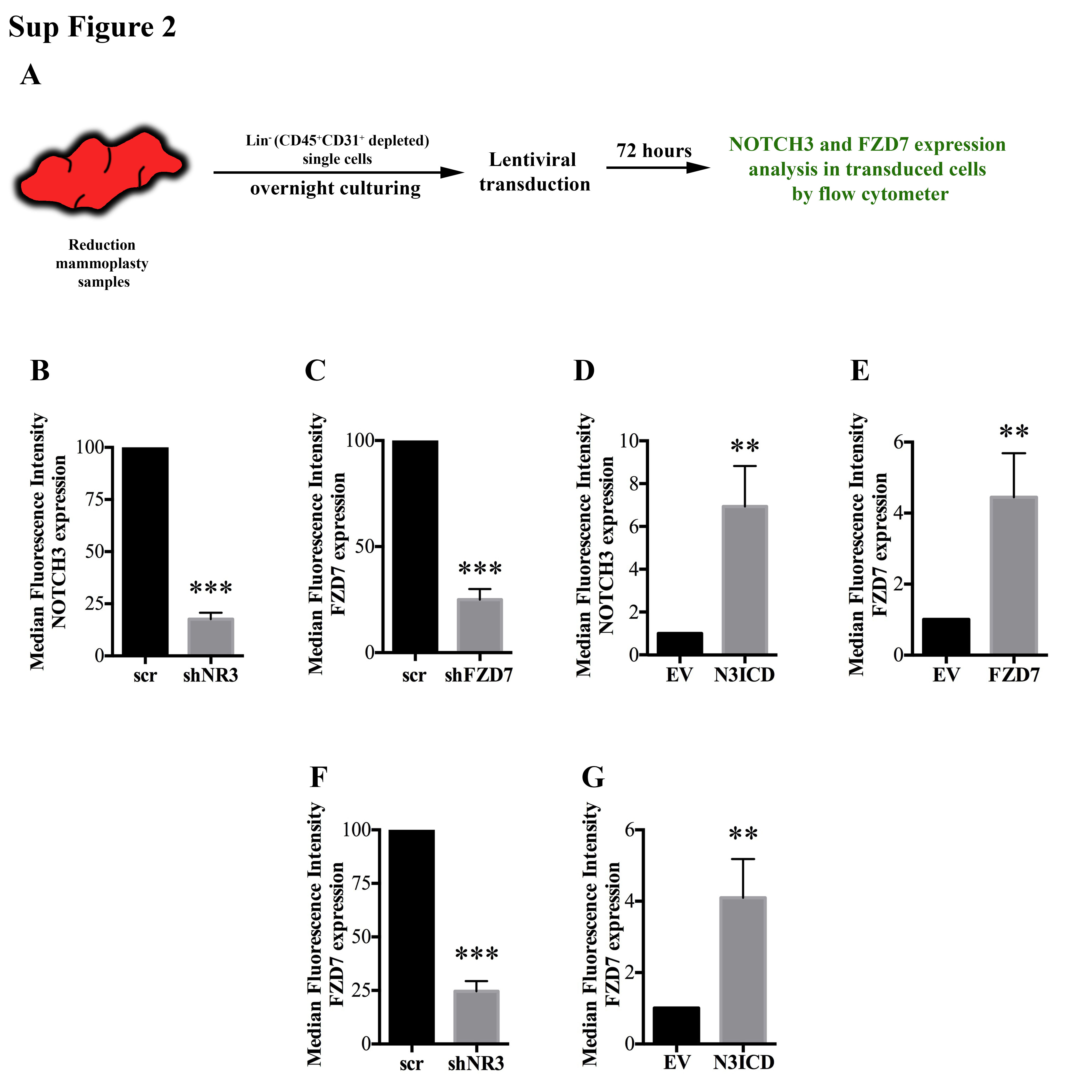

Supplement: Supplementary file 8 — Figure S2. Successful knockdown and overexpression of NOTCH3 and FZD7 in primary human breast epithelial cells. (JPG 1056 kb) [file 13287_2019_1361_MOESM8_ESM.jpg]

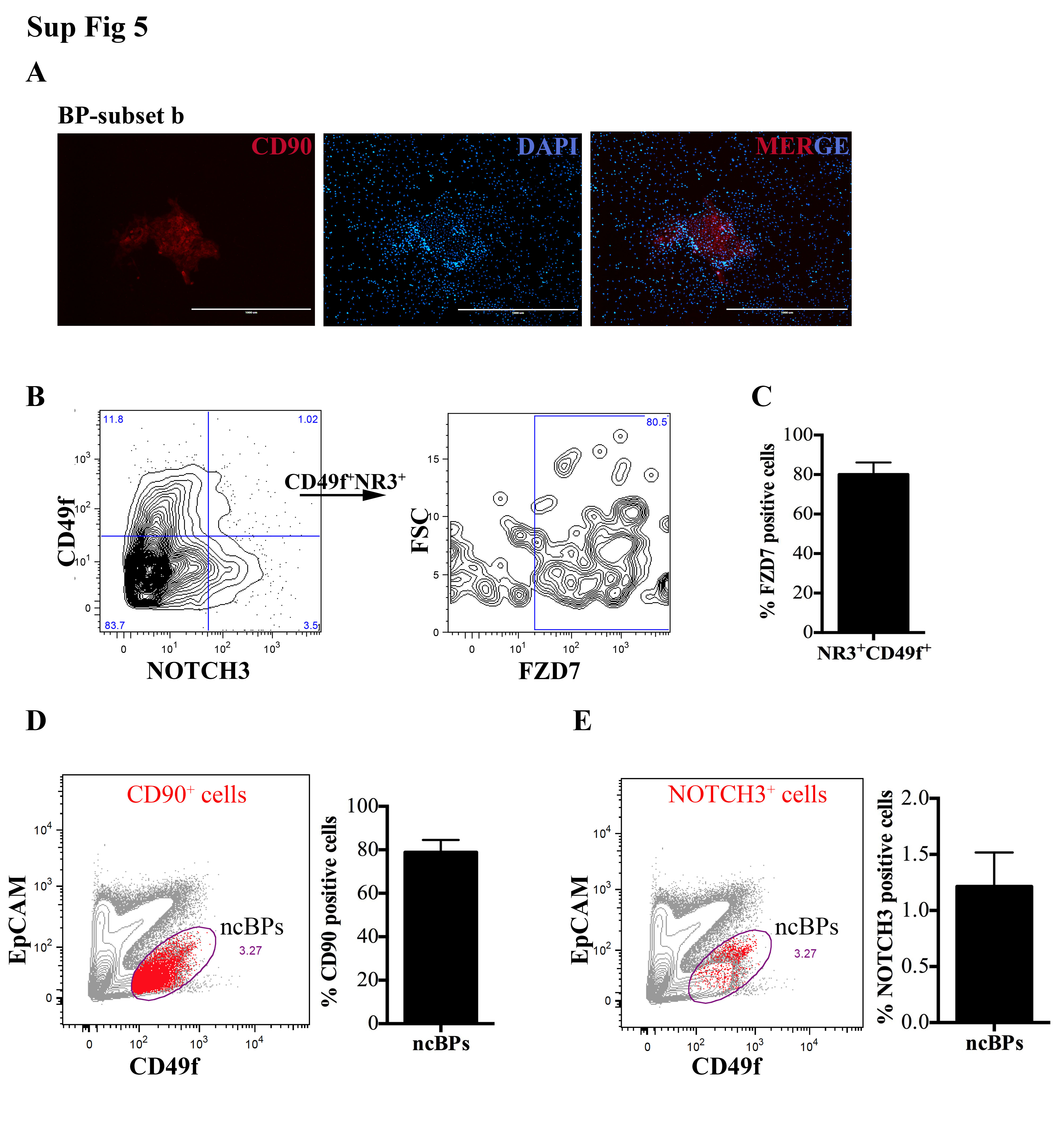

Supplement: Supplementary file 9 — Figure S5. Non-cultured bipotent progenitors can be further subdivided based on NOTCH3 expression. (JPG 1653 kb) [file 13287_2019_1361_MOESM9_ESM.jpg]

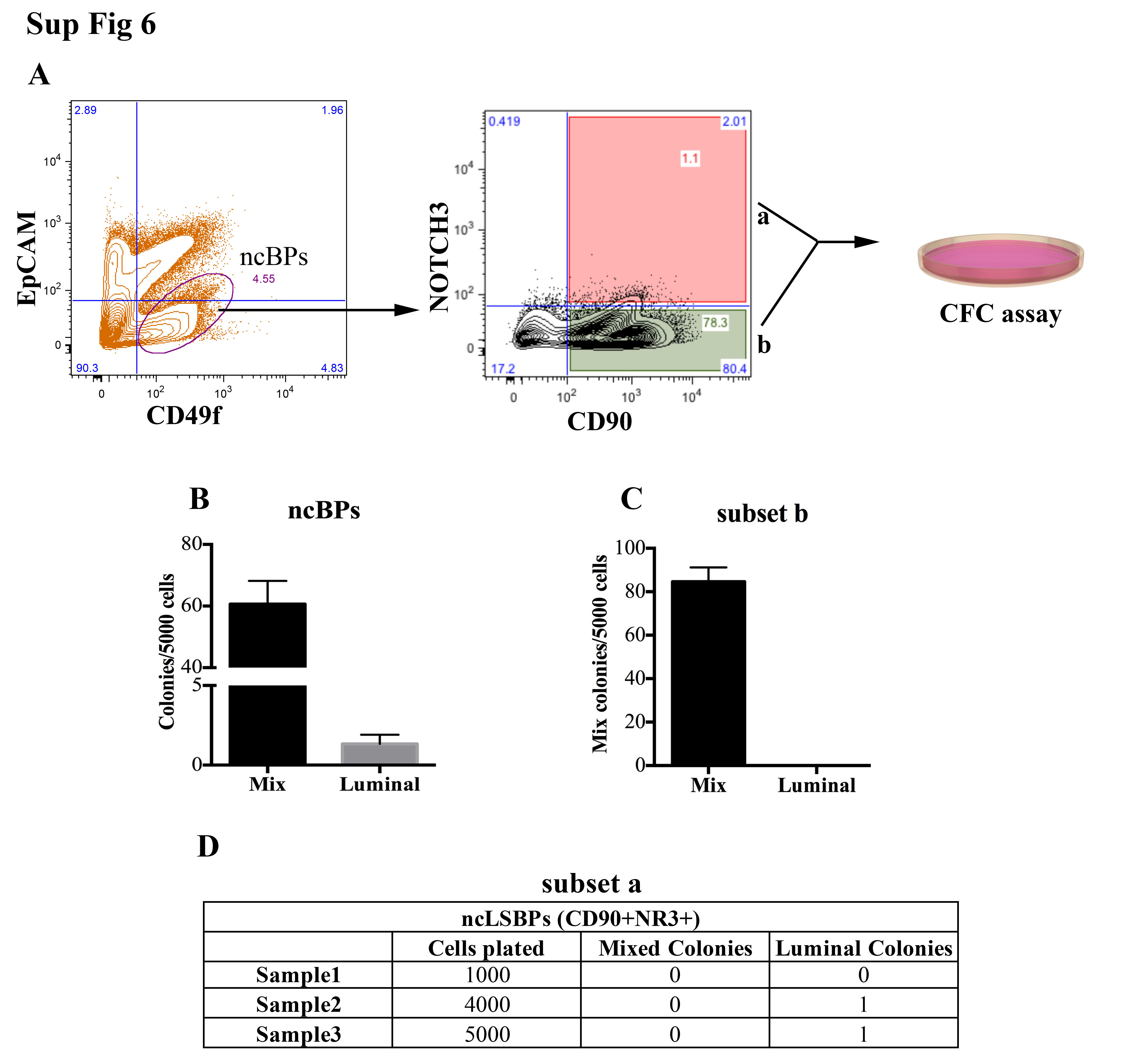

Supplement: Supplementary file 10 — : Figure S6. BLPs are detectable in non-cultured primary breast epithelial cells (JPG 1113 kb) [file 13287_2019_1361_MOESM10_ESM.jpg]
