# Supplementary material for: Isolation and characterization of a new basal-like luminal progenitor in human breast tissue
Source: Stem Cell Res Ther. 2019 Aug 23;10:269. doi: 10.1186/s13287-019-1361-3 (PMC6708178; doi:10.1186/s13287-019-1361-3)
Supplement: Supplementary file 2 — Table S3. qPCR primers. (PDF 52 kb) [file 13287_2019_1361_MOESM2_ESM.pdf]

qPCR primers

Gene		Sequence
GAPDH	Forward	GCCTCCGCTTCGCTCTC
	Reverse	CCGTTGATCCGACCTTCACC
NOTCH1	Forward	GCGGGGCTAACAAAGATATGC
	Reverse	GCACCTTGGCGGTCTCGTA
NOTCH2	Forward	GATGCCCAGGACAACATGG
	Reverse	GACTCGGTTGCGAATCAGAA
NOTCH3	Forward	CGTGGTGTCTGCCAGAGTT
	Reverse	CTGGCAGGGAGCAGTCAG
NOTCH4	Forward	TCCCCAGGAATCTGAGATGGA
	Reverse	GGACTGTACTTCCCCACAGCAAAC
FZD7	Forward	TCTCCCATTTGGATCCTTTG
	Reverse	GGACAAAATGGCTCTTTGCT
HES1	Forward	GGAAGCACCTCCGGAACCT
	Reverse	GGTCACCTCGTTCATGCACTC
ROR1	Forward	GACCGTCAGTGTGACCAAATCAG
	Reverse	GAAACGAAGGGCGGTGAAAGT
SECTM1	Forward	TCTTGGTCGCTCTGGTCATGT
	Reverse	TCATCTGGGGTTCTAGGAGGAAG
GABRE	Forward	GACACTGGCATTATCCCTTTAGG
	Reverse	GGAGAGGGAGATGTCACAGCAG
RGS2	Forward	AGAAAAGGAAGCTCCAAAAGAGA
	Reverse	GCAGTTGTAAAGCAGCCACTTGT
SCNN1B	Forward	TGAAGAATCAGCAGCCAATAAC
	Reverse	ATGATCTCCCCAAACTCGATG
ANPEP	Forward	GATTCTCCACCGAGTATGAGCTG
	Reverse	TTGATGTTGGCTTTCGTCTTCTC
CD24	Forward	TCCAGTGAAACAACAACTGGAAC
	Reverse	GTGGTGGCATTAGTTGGATTTGG
B4GALNT3	Forward	CGCCAGATGAAGACGCTGTAG
	Reverse	CACTCCCCATCCCTGAACAGTAG
CCL28	Forward	GTGTTGCTGTCAGTGCCAGTAGG
	Reverse	AGGCAATGGGAAGTATGGCTTCTG
PROM2	Forward	GACTCCTGGACTCCCTCTATGGCA
	Reverse	GTAGGGCCTTTACCAACTCTGAAGG
TRPM4	Forward	CGATGCACACACCACGGAGAAG
	Reverse	GGTCAGAGAGCCGGAGGAAATTG
EPHB3	Forward	CAGAAAGTGGGTGGGAAGAGGTG
	Reverse	AGCCAGTTGTTCTGGCTTGACTC

GGT6	Forward	GAGGACAGTGGAAAGAGTGCAGA
	Reverse	AAGGACAGAGATGCCAACAGTACC
GALNT6	Forward	CCTCTGGAACTTGGAGGGTTGTTC
	Reverse	ACGTCTGGGTCTGCGATGATTG
NEBL	Forward	CAACTGCCTGGATAAGTATTGGCA
	Reverse	GGGTAGTGTGCATTACAATAGGGCT
KRT15	Forward	GCATCAGGGAAGCCTCTTCAGG
	Reverse	TTGTGGGAAGAAACCACCTGTCC
HTATIP2	Forward	GGATGACTACGCCTCTGCCTTTC
	Reverse	CAACACGAACAAATCCCTCCGCC
PRICKLE1	Forward	GCGCGAGCAGCCATTGTTTGA
	Reverse	CTCTGACAGCCAAAGGCCAGTTT

Supplementary Table 3: List of primers

Cloning primers

Gene		Sequence
FZD7	Forward	TTggcgcgccATGCGGGACCCCGGCGCGGCC
	Reverse	CCttaattaaTCATACCGCAGTCTCCCCCTTGC
