# Supplementary material for: Isolation and characterization of a new basal-like luminal progenitor in human breast tissue
Source: Stem Cell Res Ther. 2019 Aug 23;10:269. doi: 10.1186/s13287-019-1361-3 (PMC6708178; doi:10.1186/s13287-019-1361-3)
Supplement: Supplementary file 6 — Table S1. List of genes uniquely regulated by NR3. (PDF 239 kb) [file 13287_2019_1361_MOESM6_ESM.pdf]

List of genes uniquely regulated by NR3			
ID	RefSeq	Gene.Symbol	FoldChange
16765029	NM_006121	KRT1	87.47062315
16859090	NM_012114	CASP14	40.62436432
16764907	NM_173086	KRT6C	21.88676258
16891555	NM_152386	SGPP2	21.14932919
16688992	NM_012152	LPAR3	20.06310975
17051827	NM_020299	AKR1B10	20.01292312
16748015	NM_001282424	A2ML1	18.64392014
17044253	NM_001005340	GPNMB	16.26768062
16736764	NM_001135091	MUC15	15.39830877
17112498	NM_001307940	POF1B	15.10984197
16914925	NM_001193421	TSHZ2	14.06447049
16748835	NM_001288772	PIK3C2G	14.03110443
16777185	NM_004004	GJB2	13.68019289
16854509	NM_004948	DSC1	13.42347848
16976615	NM_005420	SULT1E1	13.36100539
16840799	NM_001139	ALOX12B	11.83225655
16672489	NR_038849	LINC01133	11.16573771
16873528	XR_244001	LOC645553	10.2470969
16842563	BC037342	ERVE-1	10.07466526
16943336	NM_018004	TMEM45A	9.746133606
16774053	NM_001111045	CCNA1	9.465092904
16775546	NM_001160706	SCEL	8.705376332
16871349	NM_001244847	KRTDAP	8.063241877
16706734	NM_001171971	CDHR1	7.611302364
16707085	NM_001080518	LIPK	7.536527781
16746959	NM_001256536	PRMT8	7.469612142
16807820	NM_213600	PLA2G4F	7.272039319
16844804	NM_000526	KRT14	7.243113587
16907572	NM_001098199	GPR1	7.188147435
16723062	NM_003986	BBOX1	7.048648516
17004903	NM_001168319	EDN1	6.9046263
16819099	NM_032330	CAPNS2	6.830099836
16737344	NM_001001991	PAMR1	6.718814348
16665588	NM_001083592	ROR1	6.6776015
16764894	NM_005555	KRT6B	6.52258271
16666545	NM_001297704	ADGRL2	6.231027137
16846532	NM_005220	DLX3	6.162632099
16873518	NM_207393	IGFL3	6.15237454
16722787	NM_001098520	HTATIP2	6.037843376
16743432	NM_001271594	SESN3	5.899966107
16966621	NM_001286791	CWH43	5.872860353
16976468	NM_207407	TMPRSS11F	5.640292573
17077341	NM_138969	SDR16C5	5.604335535

16962632	NM_001134418	P3H2	5.472740057
16666755	NM_006536	CLCA2	5.456137492
16690427	NM_001102592	HENMT1	5.448327148
16697095	NM_000433	NCF2	5.406493978
16844752	NM_002275	KRT15	5.402535573
16763295	NM_001144881	PRICKLE1	5.371295154
16761269	NM_022570	CLEC7A	5.334809173
17093245	NM_004925	AQP3	5.29321162
16832499	NM_003593	FOXN1	5.224151251
16863820	NM_004605	SULT2B1	4.939517076
16990718	NM_001127698	SPINK5	4.786396788
16902279	NM_014553	TFCP2L1	4.757850696
16852871	NM_001143818	SERPINB2	4.689551654
17011279	NM_001198	PRDM1	4.64518697
16961056	NM_001040100	SPTSSB	4.63103074
16658655	NM_025106	SPSB1	4.579605524
16671104	NM_001199828	SPRR1A	4.372030099
16842417	NR_029393	KRT16P3	4.316474104
16671013	XR_917710	LOC105371443	4.30146073
16871403	NM_001166034	SBSN	4.293785137
16930371	NM_138435	FAM83F	4.243339176
16682487	NM_001136265	IFFO2	4.21954274
16911261	NM_001200	BMP2	4.067399408
17070307	NM_001444	FABP5	4.039331827
16686060	NM_006516	SLC2A1	3.980087092
16876978	NM_198182	GRHL1	3.969406554
16707096	NM_001102469	LIPN	3.940075659
17000724	NM_001945	HBEGF	3.936326799
16706630	NM_001243778	FAM213A	3.918541202
16861945	NM_001001414	NCCRP1	3.891509826
16840362	NM_001165966	PITPNM3	3.872396465
16934248	NM_174932	BPIFC	3.85742115
16671144	NM_176823	S100A7A	3.830395423
16718232	NM_001272012	ITPRIP	3.814233944
17002429	NM_025153	ATP10B	3.813643535
16671094	NM_005547	IVL	3.784711506
17004194	NM_033260	FOXQ1	3.760514344
17012148	NM_000165	GJA1	3.735392905
16807772	NM_001206670	PLA2G4E	3.718420687
16808609	NM_001276264	DUOXA1	3.699157343
16902945	NM_207363	NCKAP5	3.598003691
17095194	NM_152573	RASEF	3.59390764
16809872	XR_916373	LOC101928635	3.586193499
16711343	NM_001135241	AKR1C2	3.58485144
16796590	NM_001282237	BCL11B	3.472857786

16917004	NM_019593	GPCPD1	3.471205234
16786872	NM_020431	TMEM63C	3.418018013
16816962	NM_001039	SCNN1G	3.404886403
16715793	NM_001014797	KCNMA1	3.388202261
17071604	AK001351	LINC01181	3.385127093
16695216	NM_001135050	IGSF9	3.353645045
16908897	NM_001304536	EPHA4	3.343540672
17061545	NM_005746	NAMPT	3.323139063
16677057	NM_001146261	SYT14	3.308078187
16700878	NR_031718	MIR1537	3.242904788
16810341	NM_001218	CA12	3.226440305
16693249	NM_182578	THEM5	3.209940747
17002820	NM_001017995	SH3PXD2B	3.201763323
17103303	NM_001282167	PORCN	3.201718938
16877332	NM_145175	FAM84A	3.191924808
16893704	NM_001077710	FAM110C	3.159836696
16813038	NM_001114614	MFGE8	3.153782861
16839710	NM_001258205	TRPV3	3.123355241
16873534	NM_032040	CCDC8	3.110902832
16871235	NM_001285829	CEBPA	3.066259928
16911257	NR_109953	CASC20	3.061403766
17051553	NM_001163446	CPA4	3.051777962
17095936	NM_001003800	BICD2	3.037680212
17014442	NM_021977	SLC22A3	3.037238077
17052690	NM_001280794	EPHB6	3.035379006
16944588	NM_001145998	SLC15A2	3.019068171
16839524	NR_028502	MIR22HG	3.002886612
16867471	NR_027064	TINCR	2.986012076
16716327	NM_001031709	RNLS	2.98020175
16978054	NM_003728	UNC5C	2.95802313
16693393	NM_020393	PGLYRP4	2.955850562
16924592	NM_052954	CYYR1	2.955543252
17002464	NR_027111	LOC285629	2.949546867
16693383	NM_052891	PGLYRP3	2.945610484
16937626	NM_014229	SLC6A11	2.917332853
16877555	NM_004040	RHOB	2.912712553
16728617	NM_000804	FOLR3	2.896036931
16767208	XM_005268754	GRIP1	2.883018311
17022996	NM_002944	ROS1	2.882385568
16990848	NM_000024	ADRB2	2.880288518
17074909	NR_029875	MIR383	2.854418096
16777360	XR_914780	LOC105370107	2.843228406
16675323	NM_002923	RGS2	2.821704022
17098698	NM_001035254	FAM102A	2.787966707
16795368	NM_001256430	STON2	2.778629218

16723680	NM_001304263	LDLRAD3	2.773754302
16718126	NM_000494	COL17A1	2.772312712
16764923	NM_005554	KRT6A	2.771153576
17001901	NM_001447	FAT2	2.765921094
17047459	NR_002955	SNORA14A	2.763417099
16708177	ENST00000493385	CUTC	2.754804617
16939637	NM_001251882	VIPR1	2.753258368
16924327	NM_001100420	C21orf91	2.727129734
16769972	NM_016433	GLTP	2.726947011
16700888	NM_002508	NID1	2.715798923
16768270	NM_000899	KITLG	2.714606967
16855111	NM_001039360	ZBTB7C	2.714494072
16670710	NM_001159642	BNIPL	2.712939108
16686201	NM_001024845	SLC6A9	2.70272239
16748970	XR_242921	LOC101928387	2.698441978
16865872	XM_011508868	LOC100506374	2.694722413
17025046	NM_173515	CNKSR3	2.692183349
16818820	NM_001293557	NOD2	2.68734833
16723393	NR_015451	LINC00294	2.685207046
16700456	NM_198552	FAM89A	2.681295083
16766578	NM_001195053	DDIT3	2.661986645
17019190	NM_001164446	C6orf132	2.648587798
16980693	NM_001013415	FBXW7	2.625368617
17024685	NM_001001788	RAET1G	2.621138039
16702351	NM_001002295	GATA3	2.610519014
16866123	NM_001270938	ZNF551	2.596323224
16785709	NM_152443	RDH12	2.593936807
16994272	NM_003966	SEMA5A	2.593433421
16731773	NM_001083947	TMPRSS4	2.588381014
16860782	NM_001012320	ZNF302	2.587262915
16885135	NM_002193	INHBB	2.570590849
16839177	NM_001004431	METRNL	2.570287961
16676619	NM_182663	RASSF5	2.566389243
16700673	NM_001077397	IRF2BP2	2.561673605
16718622	NM_001003407	ABLIM1	2.558000714
16687602	NM_001114108	TTC22	2.550552859
16825970	NM_002773	PRSS8	2.546813529
16780909	NR_047700	MYO16-AS1	2.541241234
16878454	NM_001170585	PLB1	2.518852542
16995914	NM_001301873	CCL28	2.517240977
16748961	XR_242921	LOC101928387	2.500494329
16935955	NR_038956	NUP50-AS1	2.499304475
17023298	NR_026876	RNF217-AS1	2.491768431
16956316	NM_020872	CNTN3	2.48819002
16998293	NM_001118890	GLRX	2.486305082

16719025	NM_000141	FGFR2	2.483256578
16782805	NM_001143919	LTB4R	2.479552892
17088760	NM_000962	PTGS1	2.465733136
16698756	NM_001083924	C1orf116	2.46252778
16870776	NR_036455	ZNF826P	2.445359654
17001005	NM_001278613	PCDH1	2.434738225
17061129	NM_001277335	RASA4B	2.433905802
16860644	NM_001252296	CEBPG	2.427363215
16732397	NM_001244682	POU2F3	2.413611262
17009502	NM_001145652	C6orf141	2.407356829
16744918	NM_001077263	TMPRSS13	2.39987068
16819539	NM_001145770	ADGRG1	2.399360606
16710020	NM_001291876	TACC2	2.397000151
16930534	NM_001429	EP300	2.395842937
16942167	NM_020676	ABHD6	2.39324816
16854397	NM_001136205	KCTD1	2.392722908
16727074	NM_004561	OVOL1	2.387515336
17104049	NR_002953	SNORA11	2.382632865
16816978	NM_000336	SCNN1B	2.373599728
16697927	NM_012396	PHLDA3	2.367197372
16775811	NM_005708	GPC6	2.365611781
16845937	NM_014798	PLEKHM1	2.361816151
16870746	NM_001099269	ZNF506	2.354727314
16856842	NM_001300843	ZNF556	2.354569542
16729168	NM_001253891	DGAT2	2.345789134
17019019	NM_001271807	TREML1	2.343151115
16947345	XR_241604	LOC101928236	2.34255567
16723369	NM_001145541	TCP11L1	2.327562649
16999321	NM_020747	ZNF608	2.315943408
16877297	NM_021643	TRIB2	2.315943408
16791300	NM_014430	CIDEB	2.307850867
16945993	NM_001190447	PPP2R3A	2.306981871
16944574	NM_018456	EAF2	2.306310356
16902979	NM_030923	TMEM163	2.299892878
17079910	NM_001032282	KLF10	2.297003943
16820157	NM_000196	HSD11B2	2.296361861
16898326	NM_020651	PELI1	2.295820741
16779024	NM_001268	RCBTB2	2.285780177
16683290	NM_001077195	ZNF436	2.279619965
17117636	ENST00000614987	RPS6KA5	2.279456693
16952218	NM_001130964	PLCD1	2.275184172
16928372	NM_001145206	KIAA1671	2.271444413
16874978	NM_021632	ZNF350	2.266317516
16867648	NM_133492	ACER1	2.263376622
16813206	NM_001150	ANPEP	2.260914862

16784787	NM_001270520	DAAM1	2.255040483
16683377	NM_002167	ID3	2.253602913
16795794	NM_004755	RPS6KA5	2.24679734
16880678	XR_926045	LOC105374774	2.24628347
16710126	NM_002775	HTRA1	2.243627752
16938271	NM_003884	KAT2B	2.241938443
16720737	NM_001290332	SYT8	2.240136535
16875599	NM_001126132	TNNT1	2.238093021
16889879	NM_003872	NRP2	2.235720748
16861126	NM_001281443	UPK1A	2.235648431
16794555	NM_001043318	ELMSAN1	2.233227149
16821660	NM_005251	FOXC2	2.232437831
16951567	NM_000461	THRB	2.229901524
16946464	NM_001679	ATP1B3	2.229752116
16745002	NM_005797	MPZL2	2.228124735
16723695	NR_039769	MIR3973	2.223563081
16710095	NM_001001974	PLEKHA1	2.213951063
16900022	NM_001304526	KRCC1	2.209796217
17079744	NM_001042510	ZNF706	2.208969246
16825647	NM_024307	GDPD3	2.20772937
16849635	NM_003655	CBX4	2.207489639
16697893	NM_005558	LAD1	2.205526864
17012281	NM_001003395	TPD52L1	2.205389281
16738409	NM_033396	TNKS1BP1	2.205195659
16839547	NM_178568	RTN4RL1	2.203733855
16851022	NM_020412	CHMP1B	2.202822628
16882861	NM_001165977	PROM2	2.194278532
17009760	NM_001010872	FAM83B	2.192180613
16746546	NM_173593	B4GALNT3	2.188228406
17061119	NM_001277335	RASA4B	2.179942376
16887192	NR_039933	MIR4774	2.179635157
16792601	XR_943839	LOC105378179	2.178894987
16984772	NM_001102575	SNX18	2.178764099
16658289	NM_001042478	AJAP1	2.178638252
16854727	NM_001099406	SLC39A6	2.177038116
16684461	NM_001856	COL16A1	2.175931791
16971443	NM_001109977	FAM160A1	2.174429097
16864034	NM_001195227	TRPM4	2.172741689
16805870	NR_027992	NBEAP1	2.169365787
16809809	OTTHUMT0000041944	ZNF280D	2.167351778
17061117	NM_001277335	RASA4B	2.166000136
16969495	NM_001033047	NPNT	2.165709893
17008092	NM_001243186	PIM1	2.162434848
16993335	NM_014594	ZNF354C	2.159608799
16845826	NM_133373	PLCD3	2.157688597

16735738	NM_016422	RNF141	2.151903426
16813058	ENST00000560553	MFGE8	2.14974668
16718077	NM_014631	SH3PXD2A	2.149329495
16827227	NM_001128850	RRAD	2.148664153
16911517	NM_080826	ISM1	2.146223022
16674973	NM_030806	C1orf21	2.145960221
17071086	NM_024613	PLEKHF2	2.144735889
17010175	NM_024576	OGFRL1	2.140770291
16819161	NM_020988	GNAO1	2.132999065
16701975	ENST00000380859	AKR1C1	2.123989548
16663390	NR_033967	SLC2A1-AS1	2.12019457
17013664	NM_030949	PPP1R14C	2.120086801
17015021	XR_954138	LOC105374879	2.117800463
16681210	NM_014851	KLHL21	2.11505718
16749939	NM_198578	LRRK2	2.110049241
16730967	NM_207645	C11orf87	2.109069543
16922501	NM_005128	DOPEY2	2.108738206
16850344	NR_036199	MIR4315-1	2.1069752
16788036	NM_000710	BDKRB1	2.10279761
16708161	NM_020354	ENTPD7	2.101505647
16672373	NM_152501	PYHIN1	2.100258152
17117829	XR_919808	LOC105372488	2.097576363
16850998	NM_001142339	GNAL	2.097053014
17050381	NM_001007245	IFRD1	2.096524951
16991948	NM_001080428	TENM2	2.093654425
17063507	NM_030647	KDM7A	2.093567354
16998850	NM_022140	EPB41L4A	2.08924739
16867613	NM_000635	RFX2	2.086777331
16997615	NM_001174071	SERINC5	2.0863097
17112675	NM_212559	XKRX	2.083612023
17101392	NM_015691	WWC3	2.08286596
16861882	NM_001042507	LGALS7B	2.081980659
16771680	NM_001247997	CLIP1	2.08003336
16878583	NR_003074	SNORD92	2.077564595
16859117	NM_173483	CYP4F22	2.077386995
17100304	NM_001246	ENTPD2	2.075563878
16972167	NM_001873	CPE	2.074465983
17100420	NM_001004354	NRARP	2.071429424
17092579	NM_001168339	TTC39B	2.070563336
16725065	NM_001300727	DTX4	2.070252398
16977760	NM_197965	SLC10A6	2.068344741
16807071	NM_014106	ZNF770	2.063051735
16669087	NM_018420	SLC22A15	2.06247505
16832686	NM_138349	TP53I13	2.061898525
17054190	NR_024394	LINC00689	2.058818522

16833000	NM_001303542	RAB11FIP4	2.056265661
16925442	NR_038892	CBR3-AS1	2.055795367
16872022	NM_002307	LGALS7	2.051757177
17018993	ENST00000482853	OARD1	2.051235781
16866849	NM_017572	MKNK2	2.050311814
16947287	NM_001184717	TIPARP	2.048379936
17047138	NM_001199207	GTF2IRD1	2.046170928
16662755	NM_012090	MACF1	2.043827353
16712373	NM_001173484	NEBL	2.039577037
17024653	NM_032832	LRP11	2.039459229
17015207	NM_183373	PXDC1	2.037928355
16871085	NM_020856	TSHZ3	2.037481086
16742692	NM_001098816	TENM4	2.032652132
16768675	NM_018351	FGD6	2.03070405
16721835	NM_001143976	WEE1	2.030361569
16971966	NM_020840	FNIP2	2.030108263
16907546	NR_033248	GCSHP3	2.024651125
16766341	NM_003708	RDH16	2.023921498
17005564	NM_003512	HIST1H2AC	2.021388556
17044793	NR_030319	MIR550A1	2.015954857
16693357	ENST00000392653	SPRR2A	2.015838415
16995601	NM_001243093	FYB	2.015186459
16859795	NM_004864	GDF15	2.012599347
16923145	NM_004915	ABCG1	2.011734616
17070013	NM_172037	RDH10	2.006873943
16883498	NM_002518	NPAS2	2.006234158
17018186	NM_001243738	RGL2	2.001890875
16672390	NM_001206567	IFI16	2.000855064
16672654	NM_001282588	SLAMF7	1.999875237
16702007	NM_001253908	AKR1C3	1.997187801
16998682	NM_001962	EFNA5	1.992647703
16908618	NM_005689	ABCB6	1.988568143
16699904	NM_001008493	ENAH	1.987213207
16785938	NM_015351	TTC9	1.985579327
16991192	NM_002084	GPX3	1.983699276
16683300	NM_003196	TCEA3	1.978833187
16684389	NR_003066	SNORD103C	1.974996368
16743104	NM_022337	RAB38	1.97438043
16694322	NM_001256820	RIT1	1.972807241
17086746	NR_037472	MIR3910-1	1.971248968
17078745	NR_121630	CA3-AS1	1.970192595
16746057	NM_001142685	ARHGAP32	1.968554517
16774679	NM_021999	ITM2B	1.965913712
16730104	NR_002569	SCARNA9	1.963938836
16915091	NM_003222	TFAP2C	1.963049656

16939796	NM_001100594	SNRK	1.96108672
16810473	NM_014326	DAPK2	1.959410939
16968237	NR_038303	LINC01094	1.957958247
16703251	NM_001098500	KIAA1217	1.956863783
17006863	NM_005345	HSPA1A	1.955656964
16695490	NM_016946	F11R	1.954103208
16740728	NM_004292	RIN1	1.951838023
16716782	NM_020992	PDLIM1	1.950039481
17046433	NM_001170905	ZNF736	1.9440024
17083052	NM_001256876	KANK1	1.941744438
16726332	XR_913191	LOC105369340	1.939605612
16890207	NM_001039538	MAP2	1.939117197
16678536	NM_001271998	RAB4A	1.938763284
16873231	NM_198478	NKPD1	1.93690966
17055459	NM_001101417	ISPD	1.935513898
16861887	BC014786	ECH1	1.933784012
16821663	NM_005250	FOXL1	1.931752145
16916407	NM_000801	FKBP1A	1.928715031
16855973	NM_001190807	CYB5A	1.927872978
16816386	NM_001160364	TMC7	1.925655997
17002304	NM_001199380	RNF145	1.924659631
16734303	NM_001170820	IFITM10	1.920785831
16820483	XM_011522800	CDH3	1.918656792
17072653	NR_045262	PCAT1	1.916313148
17074449	NM_001080826	SGK223	1.914551763
16716213	NM_024756	MMRN2	1.914476565
16809403	NM_018728	MYO5C	1.910384893
17108827	NM_005044	PRKX	1.909242028
16786601	NR_038421	LINC01220	1.908240929
17070949	NM_001034915	ESRP1	1.907919101
16766334	NM_148897	SDR9C7	1.907852979
16910070	NR_039643	MIR4441	1.903757869
16867229	NR_002602	SNORD37	1.902953093
16702068	NM_005185	CALML3	1.902438742
16785540	NM_001024218	GPHN	1.901871798
17114707	NM_012317	LDOC1	1.901028288
16869653	NM_001300914	DNAJB1	1.898969411
17016406	NM_003543	HIST1H4H	1.898600893
16686557	NM_001114172	PIK3R3	1.897649218
17080444	XR_928577	LOC105375721	1.896798815
16889627	NR_003058	SNORD70	1.895550203
16819152	ENST00000421606	CES1P1	1.894271777
16910728	NM_001040022	SIRPA	1.893930425
16779444	NM_001282168	VPS36	1.893611011
16744991	NM_001286152	MPZL3	1.892491298

16937035	NM_018184	ARL8B	1.892189614
17008655	NM_015349	GLTSCR1L	1.890188349
16977309	NM_001300735	RASGEF1B	1.888730246
16691668	NM_001200001	NOTCH2	1.88641444
16985440	NM_001164664	MAST4	1.885346899
16775883	NM_001306070	MBNL2	1.885229288
16710453	NM_145235	FANK1	1.8850028
16813270	NM_001289910	IDH2	1.882060928
16980026	XR_939252	LOC101927490	1.880891549
16739746	NM_017878	HRASLS2	1.880756835
17022139	NM_001199563	BVES	1.880248484
16848489	NM_012121	CDC42EP4	1.874011752
16846568	NM_032595	PPP1R9B	1.873505223
17000520	NR_002913	SNORD63	1.872328183
16924320	NR_038870	C21orf91-OT1	1.870594261
16826864	NM_020807	ZNF319	1.870568329
17024335	NM_006734	HIVEP2	1.870369531
16841852	NM_178836	PLD6	1.865027194
16743926	NM_021571	CARD18	1.864398168
17100211	NM_001606	ABCA2	1.863601418
16697981	XR_426886	LOC102723465	1.863050353
16826539	NM_001012398	AKTIP	1.862968568
16676693	NM_001018053	PFKFB2	1.862086381
16820367	NM_004555	NFATC3	1.859906375
17015919	NM_001105566	KIF13A	1.856694773
16669121	NM_000701	ATP1A1	1.856506029
16830837	NR_003000	SCARNA21	1.852773683
16745343	NM_001243759	USP2	1.849587219
16777794	NM_007106	UBL3	1.848245841
16833567	NM_007026	DUSP14	1.845131109
17093373	NR_003685	SNORD121A	1.843579972
17095918	NM_022755	IPPK	1.841328034
17023414	NM_001135648	PTPRK	1.841098312
16844509	NM_001282433	KRT23	1.839996897
17079181	NR_039602	MIR378D2	1.839219074
16960355	NM_014220	TM4SF1	1.837307794
16931339	NM_001001928	PPARA	1.835534206
16915530	NR_039653	MIR548AG2	1.831725518
16697865	NM_000364	TNNT2	1.831238881
16883938	NR_003506	PLGLA	1.830515513
16742742	NR_030598	MIR708	1.830333658
16906571	NM_001243835	STAT4	1.828824539
17085685	NM_001170414	TJP2	1.82692406
16922078	NM_181615	KRTAP20-1	1.823533453
16855127	NM_001190821	SMAD7	1.822535185

16911550	NM_001033087	MACROD2	1.821112439
16721228	NM_001004750	OR51B6	1.820611796
17012182	NM_001270393	PKIB	1.819888421
16934621	NM_001177701	IFT27	1.818450935
16872452	NM_000713	BLVRB	1.817988827
16901894	NM_032494	ZC3H8	1.816259067
16964549	NM_001040101	NSG1	1.815214452
16826966	NR_002978	SNORA46	1.813189862
16838750	NM_001144888	BAIAP2	1.80827822
16794962	NR_110552	LOC102724153	1.807041952
16972305	NM_001243372	CLCN3	1.806941751
17006881	NM_005346	HSPA1B	1.806378225
16685596	NM_001271851	RRAGC	1.806211287
16875014	NM_001136499	ZNF841	1.804955581
17087900	NM_021224	ZNF462	1.80451358
16659746	NM_001024215	FBLIM1	1.804421857
16789953	NR_003693	SNORD126	1.798615325
16751900	NM_017410	HOXC13	1.796762845
16856159	NM_032510	PARD6G	1.795165265
16956591	NM_001001850	STX19	1.794427124
16873525	XR_919673	LOC105372424	1.793792897
16975084	NM_015230	ARAP2	1.792649369
17113725	NM_001122606	LAMP2	1.79180876
16696013	NM_003851	CREG1	1.790757519
16840318	ENST00000544378	NLRP1	1.790616849
16850682	NR_015389	LINC00667	1.789231422
16691619	NR_036540	LINC00622	1.788793272
16892523	NR_003006	SCARNA6	1.787021097
16877463	NM_003385	VSNL1	1.786583488
16674604	NM_001194999	MR1	1.786529826
16726936	NM_001098784	FAM89B	1.786046944
16877007	NM_001177716	KLF11	1.784887733
16758242	NM_001024808	BCL7A	1.783547947
17010639	NM_001199942	DOPEY1	1.783354276
16875034	NM_001102657	ZNF836	1.782093872
16953121	X58062	SNORD13P3	1.781999172
16798144	NR_003318	SNORD116-3	1.7797361
16798154	NR_003318	SNORD116-3	1.7797361
17005276	NM_003107	SOX4	1.779530508
17088100	NM_003358	UGCG	1.779205722
16980096	NM_015130	TBC1D9	1.776745034
16958161	NM_001031702	SEMA5B	1.773451652
17110237	NM_001126054	CASK	1.772808454
16779006	NM_001162497	LPAR6	1.772308806
16798152	NR_003323	SNORD116-8	1.772079506

16678996	XR_921395	LOC105373215	1.770720696
16986138	NM_001080479	ARHGEF28	1.770393427
17104363	NM_004429	EFNB1	1.767434397
16915635	NM_001278649	OSBPL2	1.76702608
17061433	NM_001278273	SRPK2	1.766556632
17024669	NM_001243325	RAET1E	1.765544682
17096274	NR_002894	MFSD14C	1.7651939
17080342	NM_001282902	TRPS1	1.764109362
16896579	NM_001135673	ATL2	1.763143624
16882414	NR_004378	SNORD94	1.761453838
16970906	NM_001204366	MGST2	1.760123508
17095150	NM_001303103	TLE1	1.7577217
16868397	NM_001300883	ZNF426	1.757701394
16828577	NM_001031804	MAF	1.757003015
16826367	NM_153029	N4BP1	1.756597108
16847878	NR_024386	PLEKHM1P	1.753045369
16892826	NM_020311	ACKR3	1.750422677
16878947	NM_000627	LTBP1	1.750285175
16859763	NM_006332	IFI30	1.749609954
16745380	NM_002855	PVRL1	1.749573572
17011728	XR_942870	LOC105377943	1.746552488
16813296	NR_028287	GABARAPL3	1.745249543
16905108	NM_004405	DLX2	1.744890697
17004198	NM_001452	FOXF2	1.744656883
16863011	NM_001127893	CEACAM19	1.743705823
16798148	NR_003321	SNORD116-6	1.742143343
16837061	NR_004380	SNORD104	1.741439074
16771368	NM_139015	SPPL3	1.741398839
16789400	NR_029620	MIR203A	1.738894011
16723546	NM_001752	CAT	1.73855254
16734524	NR_002982	SNORA54	1.738363755
16783675	NM_001247988	CTAGE5	1.738355722
16986734	NM_205548	FAM151B	1.737183313
17057460	NR_002919	SNORA5A	1.736493086
17055657	NM_182762	MACC1	1.735983618
17011949	NM_153711	FAM26E	1.73396727
16725112	NM_001178040	STX3	1.733466553
17075314	NM_025232	REEP4	1.73329434
16855781	NM_032160	DSEL	1.732709743
16885189	NR_023343	RNU4ATAC	1.731473131
16775655	NR_125772	LINC01068	1.729581903
16754808	NM_152588	TMTC2	1.727796532
16841463	NM_001303281	ZNF18	1.726407854
17104173	XM_006724575	KLF8	1.72582558
17014364	NM_000876	IGF2R	1.724653652

16924268	NM_003489	NRIP1	1.724589897
16757225	NM_001143906	TRAFD1	1.723821031
16911463	NM_001282550	BTBD3	1.723574111
16744370	NM_001289807	CRYAB	1.722702207
16726439	NM_012094	PRDX5	1.720741043
17098171	NR_003071	SNORD90	1.718873452
16671579	NM_001261464	ADAM15	1.718575619
16765254	NM_000966	RARG	1.717325287
17105939	NM_024539	RNF128	1.71606397
16947613	NM_139245	PPM1L	1.71605604
17047777	NM_001256414	GNAI1	1.715168125
16968529	NM_006264	PTPN13	1.715152274
17014166	XM_011535946	TULP4	1.714371771
17103951	NM_022117	TSPYL2	1.7140866
16693945	NR_040772	LOC100505666	1.713983633
17087028	NR_033937	LOC100132077	1.71367477
16846734	NM_001243877	TOB1	1.712867237
16783215	NM_001030055	ARHGAP5	1.712606057
16898655	NR_003705	SNORA36C	1.712491309
16788023	NM_000623	BDKRB2	1.711265173
17019778	NM_025048	ADGRF1	1.71120982
17088701	NM_032552	DAB2IP	1.710032013
16881242	NR_002185	OR7E91P	1.708815534
16715765	NM_032772	ZNF503	1.708381287
16979133	NM_001221	CAMK2D	1.707931365
16848219	NM_080284	ABCA6	1.707797201
16879232	NR_027252	CYP1B1-AS1	1.706933279
16816675	NR_026675	CRYM-AS1	1.70689384
16736821	NM_018490	LGR4	1.706444311
16742963	NM_001162951	SYTL2	1.704521346
16887179	NM_001256126	CERS6	1.701452253
16741023	NM_001078650	TMEM134	1.701302875
17012342	NM_138571	HINT3	1.699393557
16926253	NM_000100	CSTB	1.699095174
16750272	XR_914045	LOC105369740	1.698435777
16955197	NM_001256105	WNT5A	1.697451083
16867278	NM_001013841	STAP2	1.695828175
17097240	NM_001080551	C9orf84	1.693960225
17076634	NM_000037	ANK1	1.693302821
16913263	NM_001258329	EPB41L1	1.693068096
17097869	NM_001080497	MEGF9	1.692297643
16861890	BC014786	ECH1	1.691621342
16804716	NM_020210	SEMA4B	1.691128945
17054439	NM_001097620	TMEM184A	1.690273455
16880087	NM_138448	ACYP2	1.686812947

16821280	NM_022041	GAN	1.683896345
16857449	NM_139161	CRB3	1.683775739
16691414	NM_001007237	IGSF3	1.68292008
16937440	NM_001203263	IL17RC	1.681404299
16816018	NR_029854	MIR365A	1.680173246
17047514	NM_001110354	ZP3	1.678276008
16772074	NM_001077261	NCOR2	1.677884412
16734240	NM_019009	TOLLIP	1.677570426
16748965	XR_242921	LOC101928387	1.677151869
16666799	NM_012128	CLCA4	1.67706662
16977711	NM_002753	MAPK10	1.677043371
17012262	NM_001286398	RNF217	1.676733416
16903969	NM_001105	ACVR1	1.673629284
16866002	NM_213598	ZNF543	1.6723421
16983157	NR_003689	SNORD123	1.670905335
16886818	NM_001145909	TANC1	1.669616388
16707107	NM_001128215	LIPM	1.665917165
16931435	NM_015124	GRAMD4	1.66582094
16979698	NM_006320	PGRMC2	1.66505904
16894998	NM_001282719	LDAH	1.664755146
16892601	NM_000463	UGT1A1	1.663640064
17077135	NM_001083617	RB1CC1	1.663424823
16665302	NM_015888	HOOK1	1.663336429
16691766	AK090412	ANKRD20A12P	1.663017481
16848095	NR_030365	MIR635	1.662986742
17055131	XR_927008	LOC105375137	1.66180373
16750300	NM_001286211	TMEM117	1.660391364
17016125	NM_001503	GPLD1	1.660329984
17107371	NR_028597	SRD5A1P1	1.659739318
17113335	ENST00000386148	SNORD96B	1.659739318
16732807	NM_001130142	VWA5A	1.65970097
16694869	NM_001145312	ETV3	1.659635781
17042997	NM_001040167	LFNG	1.659524582
16900910	NM_207362	KIAA1211L	1.658045196
17055501	NM_006408	AGR2	1.657194955
17005169	NM_153042	KDM1B	1.656854215
16858451	NM_001170635	PLPPR2	1.65650589
17043588	NM_138426	GLCCI1	1.655300716
16964799	NM_020777	SORCS2	1.655300716
16813737	NM_001284417	LYSMD4	1.654570387
16940260	NM_015175	NBEAL2	1.653416283
16666965	NM_001134476	LRRC8B	1.653305501
16660360	NM_001785	CDA	1.650984604
16840235	NM_032530	ZNF594	1.650980789
16705934	NM_004273	CHST3	1.650900685

16830577	NM_001040059	CD68	1.650820585
16821562	NM_001286565	KIAA0513	1.650038858
16681142	NM_001039664	TNFRSF25	1.649821566
16722779	NR_039706	MIR4486	1.648975541
16731105	NM_022761	C11orf1	1.648030946
16842909	NM_004740	TIAF1	1.647646407
16765116	NM_001300814	KRT78	1.647440849
16682259	NR_026752	CROCCP2	1.646626482
16875514	NM_001301782	LENG9	1.645276433
16691403	NM_001144822	CD58	1.643836334
16712513	NM_153714	C10orf67	1.643616061
16809219	NM_001311175	TNFAIP8L3	1.641995303
17083996	NR_003529	CDKN2B-AS1	1.641805623
16710245	NM_001609	ACADSB	1.641612172
16684036	NM_001029882	AHDC1	1.639864563
16747336	NR_004387	SCARNA10	1.639050153
16669180	NM_020440	PTGFRN	1.638754793
16791810	NR_027263	ARHGAP5-AS1	1.638009056
16904775	NR_045786	CERS6-AS1	1.63679843
16987645	NM_198507	FAM174A	1.6365791
16693308	NM_002016	FLG	1.635743646
16990985	NM_001172698	PPARGC1B	1.635112613
16881138	NM_001202513	MXD1	1.63430434
16814336	NM_001172663	RAB40C	1.631448372
17073833	NM_145754	KIFC2	1.631316447
17062491	NM_005302	GPR37	1.631165688
16755103	NM_001301022	NUDT4	1.630585397
16978251	NM_000669	ADH1C	1.630058039
16821092	NM_033401	CNTNAP4	1.629278615
16821585	NM_001134473	GSE1	1.629154394
16776856	NM_005561	LAMP1	1.626258578
16847644	NM_001098426	SMARCD2	1.624542327
16812245	NM_004390	CTSH	1.624542327
16695268	NM_001206665	IGSF8	1.623289145
16809880	NM_001110	ADAM10	1.621662203
16958096	NM_019069	WDR5B	1.621632229
17088213	NM_001278629	C9orf43	1.620553519
16700885	XR_921388	LOC105373212	1.61977864
16692452	XM_011542528	NBPF26	1.619359536
16866650	NM_000156	GAMT	1.618884433
16765041	NM_175078	KRT77	1.618865731
16766822	NM_001136051	LRIG3	1.618263643
16956661	NM_001040181	CLDND1	1.618095397
16949911	AK128346	LINC00969	1.61604421
16864819	NM_001297624	ZNF480	1.615069968

16922543	NM_015358	MORC3	1.614823701
16830127	NM_001033002	RPAIN	1.614790122
16716590	NM_013451	MYOF	1.613507183
16825012	NM_020718	USP31	1.613317066
16924151	OTTHUMT0000015741	BAGE2	1.613279791
16674742	NM_001200050	NPL	1.61156979
17099769	NM_015447	CAMSAP1	1.611353841
16808330	uc001zsm.2	PPIP5K1	1.610427076
16791634	NR_038356	LOC100506071	1.609370693
16662159	NM_001198972	KIAA1522	1.609065811
16818204	XM_011545969	KAT8	1.607980599
16830412	NM_001251902	TNK1	1.607906296
16997503	NM_005779	LHFPL2	1.607579405
16924147	OTTHUMT0000015741	BAGE2	1.60545623
17083549	XM_011517816	KDM4C	1.605452521
17059444	NR_015381	TP53TG1	1.604295611
16715028	XM_011540211	SLC25A16	1.603947218
16855510	NM_005603	ATP8B1	1.603146942
16883675	NM_003854	IL1RL2	1.601939874
16858118	NR_004381	SNORD105	1.601928771
16981606	NM_000860	HPGD	1.60108141
16829486	NM_024792	FAM57A	1.600596878
16868862	NM_020812	DOCK6	1.600578387
17107643	NR_027456	LINC00894	1.597371634
16732716	NM_024806	C11orf63	1.597212942
16811391	NM_020214	PARP6	1.596095158
16797555	AK097859	IGHM	1.593596812
16819325	NM_001010989	HERPUD1	1.593372227
17007851	XR_926742	LOC105375032	1.593239699
16696373	NM_001185127	VAMP4	1.593132949
17066302	NM_001693	ATP6V1B2	1.592385898
16865647	NM_032701	KMT5C	1.591881928
16873104	NM_001031749	LYPD5	1.591683327
16981760	NM_000027	AGA	1.590473866
17096965	NM_018424	EPB41L4B	1.590190934
16777896	NM_001286503	HSPH1	1.590117453
17099799	NM_016172	UBAC1	1.589298373
16943107	NM_153605	CRYBG3	1.588688928
16805385	XR_916699	LOC105370986	1.587973312
17046982	NM_001305	CLDN4	1.587694493
16868418	NM_152289	ZNF561	1.587445065
16966514	NM_020453	ATP10D	1.587096664
16680420	NM_001110781	SLC35E2B	1.586590703
16919567	NM_002999	SDC4	1.586154532
16719262	NM_014661	FAM53B	1.586099561

16983451	NM_001271606	BASP1	1.585011528
16705247	NM_001282405	NRBF2	1.58484674
17113320	NM_001025580	AMMECR1	1.583635156
16950989	NM_004625	WNT7A	1.58358759
16953862	NM_001272073	QARS	1.583466852
16918201	XR_942229	LOC105372587	1.58221611
16683644	NM_015484	SYF2	1.580907912
16885853	XR_923344	LOC105373623	1.579943901
16842999	NM_001085454	GIT1	1.579867243
17071497	NM_024915	GRHL2	1.57949131
17108354	NM_001183	ATP6AP1	1.57943657
16827502	NM_001145961	SLC12A4	1.579119115
16922477	NM_001286789	CBR1	1.578943995
16924145	OTTHUMT0000015741	BAGE2	1.578207244
16929943	NM_001242923	EIF3L	1.577922848
16920121	NM_021035	ZNFX1	1.577572892
16907234	NM_001127391	ALS2CR12	1.575911662
16944724	NM_032839	DIRC2	1.57444497
16958251	NM_053025	MYLK	1.573244973
16823433	NM_001079846	CREBBP	1.57200957
16756136	NM_013320	HCFC2	1.570760619
16963981	NM_175918	CRIPAK	1.570731585
17071218	NM_002380	MATN2	1.569860828
16730268	NM_001301007	AMOTL1	1.568432382
16912167	NM_002862	PYGB	1.567646204
16932158	NM_001173533	DGCR2	1.567454248
16999421	NM_178450	03-Mar	1.567403547
16876147	NM_003433	ZNF132	1.567222484
17059249	NM_014510	PCLO	1.565980955
16874683	NM_001077491	KLK5	1.565854323
17000256	NM_001257194	KLHL3	1.565742173
17109644	NM_198279	CXorf23	1.565423853
16939592	NM_001042646	TRAK1	1.565282801
16774112	NM_001286703	UFM1	1.564624723
16949927	AK094115	TNK2-AS1	1.564010285
16821324	ENST00000566462	CMIP	1.56384768
16811192	NM_001105192	TLE3	1.563807935
16864764	NM_001031721	ZNF613	1.563555034
16886308	NM_001278579	ACVR2A	1.563334682
16985688	NM_001038603	MARVELD2	1.562904905
17117805	AK130422	LOC100127909	1.562904905
16717869	NM_001113407	LDB1	1.561930218
17068202	NM_001313994	ADAM32	1.561890521
16826243	NM_001305002	ITFG1	1.561031879
17075395	NM_001199881	EGR3	1.560725335

16769514	NM_001251904	APPL2	1.560595523
16761212	NM_005127	CLEC2B	1.560530621
16693350	NM_006945	SPRR2D	1.559579035
16902500	NR_039944	MIR4783	1.55922234
16830094	NR_034082	LOC100130950	1.558440779
17024079	NM_005923	MAP3K5	1.557911557
16728652	NM_001567	INPPL1	1.557720793
16830754	NM_001080424	KDM6B	1.556731355
17061097	NM_001277335	RASA4B	1.556123612
16922011	NM_001186	BACH1	1.556015754
16777651	NM_153371	LNX2	1.555605959
16930768	NM_004599	SREBF2	1.554502927
16861647	NM_004823	KCNK6	1.553651936
16949882	NR_003265	SDHAP2	1.553013101
16705199	NM_001244638	ARID5B	1.552962867
16781219	NM_001286732	GRTP1	1.552098374
16920299	NM_002237	KCNG1	1.551714707
16672635	NM_020335	VANGL2	1.551463762
17091750	NM_152285	ARRDC1	1.55052487
16838330	NM_004710	SYNGR2	1.55044964
16894235	NM_020738	KIDINS220	1.5504174
16725908	NR_004390	SNORA57	1.549736926
16795987	NM_004239	TRIP11	1.549296569
17014169	NM_001007466	TULP4	1.549174866
16816200	NM_001143979	NDE1	1.54892791
16851914	NM_001135178	ZNF397	1.548781187
16698500	NR_029893	MIR135B	1.547053762
16749303	NM_001001660	LYRM5	1.54687505
16721861	NM_001297714	SWAP70	1.546846458
16845817	NM_001128631	DCAKD	1.545742494
16683817	NM_001039775	AIM1L	1.545635355
16861500	NM_144689	ZNF420	1.544154027
16740914	NM_000920	PC	1.543982785
16720268	NM_022772	EPS8L2	1.543793726
16870733	NM_021030	ZNF14	1.542524424
16808306	ENST00000437065	PPIP5K1	1.541380808
16706641	NM_001128309	TSPAN14	1.540394631
16687847	NM_002353	TACSTD2	1.540394631
16797241	NM_002226	JAG2	1.540298539
16930860	NM_033318	SMDT1	1.540255834
16663621	NM_002840	PTPRF	1.53977192
16939942	NM_001134440	ZNF502	1.539512235
17044568	NM_001079864	TAX1BP1	1.538896992
17018110	NM_001270401	RXRB	1.537901744
17000000	NM_006930	SKP1	1.537088252

16661398	NM_001193308	SYTL1	1.536736701
16759622	NM_001256279	ZNF26	1.536473978
16773526	ENST00000516690	RNU6-63P	1.535959313
16747184	NM_001769	CD9	1.534973058
16855697	NM_004869	VPS4B	1.534767372
16874953	NM_001135590	ZNF577	1.534689361
16991125	NM_001301063	NDST1	1.534554623
17094814	NM_001135820	TMEM2	1.533664942
16798294	AF400494	SNRPN	1.533402744
17084280	NM_002504	NFX1	1.532396887
16873296	NM_001142502	PPP1R13L	1.532209247
17059702	NM_000466	PEX1	1.532177386
16698466	NM_030952	NUAK2	1.531904824
17072144	NM_001283012	DEPTOR	1.531763253
16849933	NM_001007533	PPP1R27	1.530330578
16846653	NR_125805	TMEM92-AS1	1.53029522
16816010	NR_132983	MIR193BHG	1.530111373
16933774	NM_001037666	GATSL3	1.529086477
17110058	NM_006520	DYNLT3	1.528538969
16852132	NM_001130110	SETBP1	1.528058737
17019218	NM_001297573	TRERF1	1.527373961
16730197	NM_017704	ANKRD49	1.524870437
16727770	NM_017857	SSH3	1.524264567
17064603	NM_170606	KMT2C	1.524194132
16850428	NR_033770	ROCK1P1	1.524063837
16894335	NM_003183	ADAM17	1.523595572
16838774	NR_036151	MIR3065	1.523215432
16933437	NM_002430	MN1	1.522863535
16924143	OTTHUMT0000015741	BAGE2	1.522304187
16675698	NM_001142569	C1orf106	1.521920852
17109272	NM_001018109	PIR	1.521411062
16895954	NM_001142683	CCDC121	1.521038496
16829302	NM_001256182	ANKRD11	1.52101741
16935178	NM_005740	DNAL4	1.520609806
17101193	XM_005274530	CD99	1.520469279
16814065	NR_034090	DDX11L9	1.520247974
16676461	NM_002596	CDK18	1.52013909
16888157	NM_001042702	DFNB59	1.519471905
16764882	NM_004693	KRT75	1.519370098
17003593	NM_005649	ZNF354A	1.519268297
17052289	NR_024451	JHDM1D-AS1	1.519233195
16998112	NM_001163417	FAM172A	1.518976974
16980632	XR_939340	LOC105377486	1.518973464
16982828	NM_024337	IRX1	1.51713556
17015064	NM_001012418	MYLK4	1.516991848

17019805	NM_014452	TNFRSF21	1.516375095
16917139	NM_020341	PAK7	1.516070315
16713725	XR_945926	LOC105378289	1.515355898
16759694	NM_001165881	ZNF268	1.515054824
16888847	NM_005966	NAB1	1.514204436
16782042	M87866	OR6C4	1.513462924
16837172	NM_014405	CACNG4	1.513151737
16808318	ENST00000465123	PPIP5K1	1.512903533
17053051	NM_020781	ZNF398	1.51279867
17011261	NR_037157	BVES-AS1	1.512658864
16792268	NM_001079537	TRAPPC6B	1.511411669
16795047	NM_024496	IRF2BPL	1.511254533
16911923	NM_001163022	KIZ	1.510880963
16824166	NR_123721	PKD1P6	1.51077275
16756202	NM_001008394	EID3	1.510235289
17049667	NM_030961	TRIM56	1.510001518
16984945	NM_005921	MAP3K1	1.509865459
17093300	NR_003667	SUGT1P1	1.5095585
17100075	XR_930442	LINC01451	1.509122585
16662710	NR_037432	MIR3659	1.506067746
16824481	NM_015092	SMG1	1.505852016
16952202	NR_110531	ITGA9-AS1	1.505058955
16684024	NM_001201404	WASF2	1.504238506
16793299	NR_026796	LINC00520	1.50372769
16815425	NM_001302109	ZNF75A	1.503474084
17008815	NM_001270398	PTK7	1.503071182
16878920	NR_039922	MIR4765	1.502869771
16664748	NM_015696	GPX7	1.502835048
16947113	NM_002886	RAP2B	1.502737827
16760079	NR_126055	THCAT155	1.502057457
16825834	NM_001024683	ZNF688	1.501432899
16990334	NM_002588	PCDHGC3	1.500721912
16872089	NM_001193286	SIRT2	1.50046188
16981588	NM_012180	FBXO8	1.500406412
17062625	NM_022143	LRRC4	1.500399478
17063100	NM_001628	AKR1B1	1.500271218
16851565	NM_001135993	TTC39C	-1.500184561
17005569	NM_005321	HIST1H1E	-1.500281617
16856257	NM_002819	PTBP1	-1.500489614
16795755	NM_001010854	TTC7B	-1.500697641
16968431	NM_001297767	MRPS18C	-1.500819003
16843417	NM_001289009	SLFN12	-1.502220579
16946866	NR_036538	LOC646903	-1.503012145
17057035	NM_002787	PSMA2	-1.503349034
17059551	NM_001256891	SRI	-1.50395354

16677153	NR_026761	LINC00467	-1.504422721
16950094	NM_032773	LRCH3	-1.504812078
17014064	NM_024630	ZDHHC14	-1.505678064
17098364	NM_001144877	SCAI	-1.505872892
16757863	NR_038924	PXN-AS1	-1.506899637
17098655	NM_175039	ST6GALNAC4	-1.508087357
16984562	NM_001178055	PARP8	-1.508240679
17016624	NM_006510	TRIM27	-1.508265073
16853330	NM_001171967	RBFA	-1.508470692
17079362	NM_001199975	UQCRB	-1.509548036
16946393	NM_001303245	RASA2	-1.509942209
16958303	NM_001308317	CCDC14	-1.510067808
16664048	NM_000374	UROD	-1.510640112
17023758	NM_052831	SLC18B1	-1.510664544
16910953	NM_030811	MRPS26	-1.511464052
16891616	NM_022915	MRPL44	-1.511729484
16793096	NM_001160147	DDHD1	-1.511841259
16711383	NM_001171864	TUBAL3	-1.512159164
17056098	NM_005522	HOXA1	-1.512246512
17062832	NM_001282190	ZC3HC1	-1.512354831
16746696	NM_024551	ADIPOR2	-1.512498103
16844248	NM_001079518	MED24	-1.513134257
17091648	NM_001144026	NDOR1	-1.513291589
16783623	NM_001009182	GEMIN2	-1.513333546
16664947	NM_016491	MRPL37	-1.513361519
16795791	XR_915902	LOC105370623	-1.514113476
17101815	NM_001168683	TXLNG	-1.514288404
16951990	NM_015551	SUSD5	-1.51451234
16923491	NM_005049	PWP2	-1.514837807
17048842	NR_002147	MYH16	-1.514974314
16959187	NM_007208	MRPL3	-1.515086329
16863344	NM_001163377	QPCTL	-1.515467941
17006087	NM_001098478	HLA-F	-1.516434657
17096436	NM_014788	TRIM14	-1.516525756
16753710	ENST00000247815	HELB	-1.516651902
16917468	NM_001042576	RRBP1	-1.516662415
16708757	NM_001136200	BORCS7	-1.516799086
17095264	NM_001135953	GKAP1	-1.516949789
16749826	NM_001278463	DNM1L	-1.517170614
16763373	ENST00000344862	PUS7L	-1.518268203
17005348	NM_001286264	MRS2	-1.518285743
16792715	NM_001282236	CDKL1	-1.518369937
16688665	NM_005482	PIGK	-1.518857653
16802162	NM_001143688	DIS3L	-1.519096304
16955048	NM_052859	RFT1	-1.519285848

17104122	NM_014061	MAGEH1	-1.519598297
16840145	NM_001165417	SLC25A11	-1.519633407
16708668	NR_038937	RPARP-AS1	-1.519808972
16902749	NM_001171083	SMPD4	-1.52010748
16910117	NM_173351	OR6B3	-1.520163676
16912905	NM_001257137	ITCH	-1.520205824
16822438	NM_021259	TMEM8A	-1.520342815
16677324	NM_014053	FLVCR1	-1.520430636
16745870	NM_001243597	CDON	-1.52064494
16800406	NM_016396	CTDSPL2	-1.521716916
16741201	NM_001277	CHKA	-1.52257504
16705381	NM_030625	TET1	-1.522803721
16670364	XM_005276144	LOC100996721	-1.522817794
16685144	NM_001199779	PSMB2	-1.523229509
16909319	NM_139072	DNER	-1.523750471
16683875	NM_018066	GPN2	-1.524549859
17066650	NM_001018003	SORBS3	-1.524574517
16758135	NM_001080825	TMEM120B	-1.525064225
16847760	NM_000873	ICAM2	-1.525229845
16972764	NM_001291959	ING2	-1.525945391
16977378	NM_001080506	TMEM150C	-1.526590727
16958487	NM_003794	SNX4	-1.526700074
16876310	ENST00000341154	SRSF10	-1.527042272
17112137	NM_021963	NAP1L2	-1.527049329
17084838	NM_001316345	RECK	-1.527112838
16711797	NM_014142	NUDT5	-1.527116367
16705287	NR_120647	LINC01515	-1.527709251
16919466	NM_000022	ADA	-1.52828471
16874313	NM_017916	PIH1D1	-1.528546033
16879408	NM_001145076	EML4	-1.528623732
16687132	NM_015913	TXNDC12	-1.52909001
16707196	NM_001270927	IFIT1	-1.529796763
16832610	NM_005702	ERAL1	-1.529842713
16940012	NM_003241	TGM4	-1.529906339
16973693	NM_001303143	HAUS3	-1.530581641
16814872	NM_006453	TBL3	-1.530634688
16984244	NM_001168355	OSMR	-1.53083982
17058121	NM_001130022	ZNF680	-1.531809262
17086314	NM_001001551	IDNK	-1.532442915
16943603	NM_001142568	BBX	-1.532800567
16889845	NM_001302769	PARD3B	-1.533526751
17099549	NM_001145099	SLC2A6	-1.533636594
17105809	NM_194324	TMSB15B	-1.534122124
16962481	XR_924799	LOC105374258	-1.534320633
17004342	NM_001128591	PSMG4	-1.53576414

16774071	NM_000538	RFXAP	-1.535820915
16688910	XR_947543	LOC105378818	-1.535930923
16961374	NM_032487	ACTRT3	-1.536051585
17008534	NM_001134493	TOMM6	-1.536413629
16939558	NM_001098209	CTNNB1	-1.536811266
16850896	NM_020648	TWSG1	-1.536878732
16674767	NM_001357	DHX9	-1.536910691
16944738	NM_006810	PDIA5	-1.537390153
16709072	NM_001121	ADD3	-1.537454093
16907222	XR_923785	LOC105373836	-1.53751093
16895647	NM_013388	PREB	-1.537677902
16743403	NR_031630	MIR548L	-1.537866212
16792859	NM_001163940	PYGL	-1.537926618
16904514	NM_001081676	SCN3A	-1.53861968
16863922	NM_001291428	BAX	-1.540102815
16899429	NM_001201334	GCFC2	-1.541063881
16756649	NM_001093	ACACB	-1.541202751
17070998	NM_017864	INTS8	-1.541533954
16729712	NM_001308007	EED	-1.541957854
16729475	NR_102280	KCTD21-AS1	-1.542446019
16975918	NM_000232	SGCB	-1.542642041
16730061	NM_033395	CEP295	-1.543825829
16761148	NR_033399	DDX12P	-1.544018459
17053316	NM_001142928	LRRC61	-1.544100512
16889929	XR_923805	LOC105373849	-1.545296131
16814528	NM_022092	CHTF18	-1.545342546
16894452	NM_001282704	PDIA6	-1.546185414
16836476	NM_014906	PPM1E	-1.547851071
16941648	NM_001124767	SMIM4	-1.548627321
16977789	NM_001292003	KLHL8	-1.548684572
16910698	NM_014723	SNPH	-1.549500621
16802854	NM_001024736	CD276	-1.549554324
16735443	NM_005418	ST5	-1.550266953
16670332	ENST00000430442	LINC00869	-1.550381578
16873949	NM_006801	KDELR1	-1.550478299
16677259	NM_013349	NENF	-1.551621494
16902411	NM_004305	BIN1	-1.553074102
17064459	NM_005692	ABCF2	-1.553174579
16698543	NM_001135662	RAB29	-1.553472461
17068636	NM_001277971	POMK	-1.553540659
16876648	NR_038429	RNASEH1-AS1	-1.553695013
16803540	NM_001102667	PSMA4	-1.553723731
17055295	NM_001135924	VWDE	-1.553738091
16871305	NM_175872	ZNF792	-1.554215621
16856476	NM_001001975	ATP5D	-1.554445462

16660828	NM_001303448	SRRM1	-1.554574762
17050645	NM_018412	ST7	-1.555178307
17068014	NM_001164232	DDHD2	-1.555548453
17019689	NM_001290072	ENPP5	-1.5555808
17015084	NM_030666	SERPINB1	-1.556141589
16938647	NM_178868	CMTM8	-1.556587489
16714840	NR_031566	MIR1296	-1.556627051
16830631	NM_001143990	WRAP53	-1.55665942
16810564	NM_001301302	OAZ2	-1.556792502
16972425	NM_003864	SAP30	-1.5569184
16996645	NM_014473	DIMT1	-1.557055102
16685571	NM_001243878	FHL3	-1.557386113
16693515	NM_001267809	ILF2	-1.557465278
16836985	NM_002401	MAP3K3	-1.558044745
16784947	NM_001177963	MNAT1	-1.558667644
16728994	NM_006591	POLD3	-1.558793694
16869939	ENST00000587970	LOC105372290	-1.559218738
16820414	NM_001184824	PRMT7	-1.559308804
17022929	NM_000493	COL10A1	-1.559402479
16891042	NM_014640	TTLL4	-1.559946625
17103067	NM_001170460	CDK16	-1.560462117
17068541	NM_001135694	VDAC3	-1.560995812
16900293	NM_001310154	ANKRD36C	-1.561374558
16778703	NM_198404	KCTD4	-1.561695662
17103804	NM_001005332	MAGED1	-1.563247995
16798755	NR_026858	ULK4P1	-1.563363579
17044423	NM_007276	CBX3	-1.564317474
16896442	NM_001135651	EIF2AK2	-1.564733178
17056227	NM_175061	JAZF1	-1.564928417
17117874	XR_245037	LOC101928152	-1.565405769
16714880	NM_021800	DNAJC12	-1.565919447
16840442	ENST00000570562	ALOX12-AS1	-1.56640072
16987766	NM_033211	C5orf30	-1.567160927
16763104	NM_153634	CPNE8	-1.567345604
17112471	NM_001177478	HDX	-1.567769358
16769807	ENST00000326495	SSH1	-1.56778747
16848062	NM_181655	C17orf58	-1.56819685
16930199	NM_021822	APOBEC3G	-1.568225836
16857429	NM_006012	CLPP	-1.568638955
17057026	NM_001099858	C7orf25	-1.568671574
16723422	NM_012194	KIAA1549L	-1.568689696
16935623	NM_015703	RRP7A	-1.569131941
17060237	NM_001003713	ATP5J2	-1.56973751
16954354	NM_006764	IFRD2	-1.569752018
17009615	NR_024403	LOC730101	-1.570042196

17085196	NM_001012421	ANKRD20A2	-1.570873129
16928098	NM_002415	MIF	-1.571257902
17101465	NM_001193270	MSL3	-1.571410385
17098900	NM_032799	ZDHHC12	-1.571842501
17084184	NM_001278352	ACO1	-1.571878819
16685779	NM_000310	PPT1	-1.572042259
16868619	NM_004230	S1PR2	-1.572267471
16726164	NM_001300800	NAA40	-1.572332861
16793475	NM_022571	GPR135	-1.573783041
16683788	NM_001077262	UBXN11	-1.574470434
16906169	XR_251229	LOC101929976	-1.575048951
16954048	NM_000581	GPX1	-1.57531463
16726183	NM_004074	COX8A	-1.575416546
16962277	NM_001166415	EHHADH	-1.575715053
17060857	NM_001084	PLOD3	-1.576982518
16729051	NM_006656	NEU3	-1.577791605
16946762	NM_001184720	GYG1	-1.577798896
16972850	NM_001300767	PRIMPOL	-1.578418751
16932204	NM_001256534	SLC25A1	-1.578674056
16822084	NR_036480	VPS9D1-AS1	-1.578765247
16771341	NM_015918	POP5	-1.579790589
16836277	NM_001102402	PCTP	-1.580214057
16962264	NM_022149	MAGEF1	-1.580261521
17102948	NR_015378	ZNF674-AS1	-1.580637638
16995809	NM_000436	OXCT1	-1.580933481
16802653	NM_001286429	THSD4	-1.581360909
16699066	NM_001198862	TMEM206	-1.582201487
16760516	NM_001142961	LPAR5	-1.582749932
16826779	NM_018110	DOK4	-1.583825434
16732419	NM_001198670	TMEM136	-1.584103574
16999147	NM_001308081	DTWD2	-1.585828399
16995333	NR_046262	NIPBL-AS1	-1.58619118
16851486	NM_000227	LAMA3	-1.587500082
16833488	NM_024864	MRM1	-1.58798065
17009620	NM_014051	TMEM14A	-1.588391633
16834395	NM_016437	TUBG2	-1.589173528
16706200	NM_003373	VCL	-1.589606856
16752834	NM_001166356	SHMT2	-1.589874991
16844099	NM_000723	CACNB1	-1.589919072
16900286	AK057596	LINC00342	-1.589930093
16959871	NM_001178138	TFDP2	-1.590058671
16748126	NR_024374	LOC642846	-1.590396698
16718395	NM_001195304	BBIP1	-1.590525314
16792226	NM_006364	SEC23A	-1.590823008
16705260	NM_001001330	REEP3	-1.590999446

16670141	NM_016334	GPR89B	-1.591293552
16776658	NM_015205	ATP11A	-1.59184147
16858805	NM_001271043	NFIX	-1.59206584
17093417	NM_020702	KIAA1161	-1.59254779
16737200	NM_000611	CD59	-1.59268394
17110086	NM_000328	RPGR	-1.59269498
16940065	NM_015340	LARS2	-1.593350138
16949292	NM_016306	DNAJB11	-1.593508447
17045001	NR_036501	LINC00997	-1.593699911
16977892	NM_152542	PPM1K	-1.593718323
17064230	XM_011516502	ACTR3C	-1.593961371
16667183	NM_001164391	MTF2	-1.594060811
16803533	NM_001013619	HYKK	-1.59413079
16886503	NM_001177663	RIF1	-1.594233924
17072104	NR_038210	SAMD12-AS1	-1.594381269
17074815	NM_152271	LONRF1	-1.595048177
17002373	NM_001308173	CCNJL	-1.595472048
17070634	NM_003821	RIPK2	-1.595766981
16755299	NM_003095	SNRPF	-1.596814434
16867505	NM_001308240	C19orf70	-1.597279369
16818165	NM_001122957	BCKDK	-1.597294131
16728189	NM_139075	TPCN2	-1.598782109
17050055	NM_021930	RINT1	-1.598988984
16892075	NM_001271466	ARMC9	-1.599314129
17099083	NM_000113	TOR1A	-1.599521074
17015114	NM_001195291	SERPINB6	-1.599735437
16950173	NM_001145248	FAM157A	-1.599750222
16964100	NM_001141936	C4orf48	-1.600145765
16954685	NM_000992	RPL29	-1.600500728
16874156	NM_001161587	GYS1	-1.600693033
16936585	NM_033200	LMF2	-1.601925069
16795698	NM_001284266	EFCAB11	-1.602684002
16878699	NM_001002257	LCLAT1	-1.60358408
16959628	NM_016216	DBR1	-1.603710057
16677407	NM_020197	SMYD2	-1.603991689
16763853	NM_001267594	SENP1	-1.604391988
16969344	NM_001008388	CISD2	-1.604818342
16898518	NM_000945	PPP3R1	-1.606376423
16699864	XR_921202	LOC102723817	-1.609445064
17062000	NR_015442	LINC00998	-1.611543726
16928533	NM_020437	ASPHD2	-1.611849078
16941344	NM_000688	ALAS1	-1.612120965
16767270	NM_001205028	MDM1	-1.612240163
16696785	NR_037167	LOC730102	-1.613771892
16753533	NM_178169	RASSF3	-1.614506594

16866565	NM_001316323	POLR2E	-1.615353595
16975310	NM_001184700	UGDH	-1.615390918
17110481	NM_001114123	ELK1	-1.615771662
16777224	NM_022459	XPO4	-1.615850062
16975467	XR_925242	LOC105374421	-1.616055412
16810514	NM_000942	PPIB	-1.616529684
17025595	NM_145169	SFT2D1	-1.61688081
16861553	NM_001300993	ZNF570	-1.617960814
16888047	NM_003659	AGPS	-1.618499216
16696237	NM_000655	SELL	-1.618716123
17018530	NM_003137	SRPK1	-1.618734823
17115692	NM_001166460	MPP1	-1.619430626
17098866	NR_029837	MIR219A2	-1.619692565
16662525	NM_017825	ADPRHL2	-1.61992086
16922613	NM_005069	SIM2	-1.620770701
16863460	NM_001204284	PPP5C	-1.621261341
17021978	NM_001013399	CCNC	-1.621437409
16881434	NM_003124	SPR	-1.622621677
16707343	NM_001284274	HECTD2	-1.622925378
17093507	NM_001282205	SIGMAR1	-1.623154129
16857047	NM_172251	MRPL54	-1.623229137
16843374	ENST00000345365	RAD51D	-1.623517948
16752624	NM_001099337	COQ10A	-1.623630485
16779059	NM_001079670	CAB39L	-1.625988064
16961003	NM_173084	TRIM59	-1.626115801
16935195	NM_001303494	CBX6	-1.626378821
16743342	ENST00000525928	TAF1D	-1.627359885
16734163	NM_001142674	CHID1	-1.628379164
16756212	NM_001173982	CHST11	-1.628800602
17087329	NM_001166116	TMOD1	-1.629459318
17005945	NM_001184743	PGBD1	-1.629956354
16771998	NM_145058	RILPL2	-1.630404569
16678851	NM_032435	KIAA1804	-1.630770013
17046601	NM_000048	ASL	-1.632017658
16947532	NM_000882	IL12A	-1.632213749
16699009	NM_014873	LPGAT1	-1.632870072
16882142	NM_021103	TMSB10	-1.632896482
16985794	NR_033417	GTF2H2B	-1.633002123
16665475	NM_032852	ATG4C	-1.633677636
16702443	NM_153256	PROSER2	-1.634425177
16820883	NM_001017967	MARVELD3	-1.634651773
16819856	NM_144601	CMTM3	-1.634678211
16692892	NM_022075	CERS2	-1.634889731
16973180	NR_033869	LINC01060	-1.635010613
16840885	NM_032354	TMEM107	-1.635513121

16763375	NM_001098614	PUS7L	-1.635981764
16730180	NM_015368	PANX1	-1.636046024
16978959	NM_001226	CASP6	-1.636439198
16805541	NM_015286	SYNM	-1.637452814
17000928	NM_022481	ARAP3	-1.638444344
16674998	NM_007212	RNF2	-1.639826674
16850322	NM_001103154	ARL17B	-1.640592191
16851768	NM_001943	DSG2	-1.640789313
16984725	NR_034107	LOC257396	-1.642621402
16681192	NM_024654	NOL9	-1.643202179
16933088	XM_011546907	GSTT2B	-1.64407563
16748196	NR_002814	LOC374443	-1.644611323
16866337	NM_005762	TRIM28	-1.645580572
16894848	NM_001006657	WDR35	-1.646139576
17055630	NM_001002926	TWISTNB	-1.648069024
17080468	NM_001101676	SAMD12	-1.648514603
16902623	NM_001145928	SAP130	-1.648945062
16815652	NM_019109	ALG1	-1.649253691
17083742	NM_001114395	CNTLN	-1.650893056
16839220	NM_182705	FAM101B	-1.652308796
16967875	NM_015393	PARM1	-1.652583689
16688753	NM_001303433	FUBP1	-1.653294041
16929509	NM_001003681	HMGXB4	-1.653645511
17101422	NM_001256944	CLCN4	-1.653756316
17065958	NM_006765	TUSC3	-1.656502063
16946207	NM_001033030	FAIM	-1.657623851
16819827	NM_001040138	CKLF	-1.657876645
16828199	NR_126330	LINC01572	-1.658443657
17114643	NM_001010986	ATP11C	-1.658700409
16885978	NM_001282798	R3HDM1	-1.658857545
16907656	NR_110283	LOC101927865	-1.658918871
16981516	XR_939462	LOC105377540	-1.658999364
16999817	NM_001300791	KIF3A	-1.659083695
16840293	NM_001199699	DHX33	-1.659455566
16951306	NM_015150	RFTN1	-1.659631947
16900628	NM_016466	ANKRD39	-1.661400622
16749759	NM_001003398	BICD1	-1.662571823
16934749	NM_002872	RAC2	-1.663559345
17088408	NM_153045	C9orf91	-1.66368619
16913065	NM_006404	PROCR	-1.664651297
16935537	NM_000262	NAGA	-1.665947958
16685036	NM_001195156	ZMYM6NB	-1.666698713
16862078	NM_016941	DLL3	-1.667072291
16860834	NM_001136199	GRAMD1A	-1.667811992
16713419	NR_026777	ZNF37BP	-1.668151131

17060448	NM_001190415	TAF6	-1.669531523
16854744	NM_001271949	TPGS2	-1.670584935
16820508	NM_024562	TANGO6	-1.671156294
16820041	NM_001950	E2F4	-1.671499975
17021188	NM_001199917	PGM3	-1.671666049
17117547	AY358681	LOC100130428	-1.67204074
16702479	NM_001142627	SEC61A2	-1.672747862
16985162	NM_001134779	IPO11	-1.673625417
16887313	NM_001008489	PHOSPHO2	-1.674472481
17097369	NM_001101338	ZNF883	-1.674871021
16686010	NM_001146289	P3H1	-1.675517398
16946553	NM_001080415	U2SURP	-1.6768109
16707983	NM_001287803	ZDHHC16	-1.678539709
16835517	NM_001160423	IGF2BP1	-1.678570735
17014997	NM_148959	HUS1B	-1.68005679
16953811	NR_029690	MIR191	-1.680685752
17078916	NM_001024688	NBN	-1.680844971
16921472	OTTHUMT0000047683	IGHV1OR21-1	-1.681210067
16661508	NM_001009568	SMPDL3B	-1.681299411
17056248	NM_019029	CPVL	-1.681738431
16823750	NM_001042476	CARHSP1	-1.681761745
16753689	NM_033647	HELB	-1.683254514
16899601	NM_001080824	TRABD2A	-1.683732946
16758671	NM_001143850	TCTN2	-1.684429445
16734154	NM_021128	POLR2L	-1.684857604
16939203	NM_005108	XYLB	-1.685753197
17088006	NM_001004065	AKAP2	-1.686505083
17084710	NM_001195200	CCDC107	-1.686910384
16946173	NM_031913	ESYT3	-1.687132561
17045806	NM_001281768	ADCY1	-1.687974762
16826212	NM_001001436	C16orf87	-1.688696425
16960371	NM_001168278	WWTR1	-1.689055421
16948236	NM_001303425	ZNF639	-1.68937546
16898601	NM_001002755	NFU1	-1.689886868
16887468	NM_001290030	ERICH2	-1.690367186
17080408	NM_006265	RAD21	-1.693103302
16707202	NM_012420	IFIT5	-1.693251961
16845657	NM_001143780	SLC25A39	-1.694108959
16796854	NM_001272011	MOK	-1.694339914
16933470	NM_001145418	TTC28	-1.694555239
16966049	NM_001007075	KLHL5	-1.694692279
17074848	NM_001164271	DLC1	-1.694786255
16943241	NM_001850	COL8A1	-1.694954642
16895941	NM_022823	FNDC4	-1.696020177
16688904	BC042527	TTLL7-IT1	-1.696153416

16846714	NM_052855	ANKRD40	-1.696361133
16930758	NM_001304797	CCDC134	-1.696757041
17058758	NM_001145064	GATSL2	-1.697482459
16728141	NM_015973	GAL	-1.697580513
16940738	NM_001640	APEH	-1.697996321
17086418	NR_029410	LOC389765	-1.69847502
17084321	NM_001244752	ANKRD18B	-1.700685844
16868661	NM_133452	RAVER1	-1.705108251
16686376	NM_020883	ZSWIM5	-1.705600776
16700135	NM_001161465	JMJD4	-1.705837239
16732296	NM_005188	CBL	-1.706657231
16895452	NM_002254	KIF3C	-1.708045808
16690535	NM_001005290	PSRC1	-1.708329974
16985720	NM_001042490	GTF2H2C_2	-1.708724727
16660403	NM_001256416	NBPF3	-1.708855016
17058826	NM_001243198	HIP1	-1.709356523
16678496	NM_175055	HIST3H2BB	-1.709522408
16996771	NM_019072	SGTB	-1.709688309
16881653	NR_045634	BOLA3-AS1	-1.710032013
16811975	NM_001168412	TSPAN3	-1.71124145
16962969	NM_001011655	TMEM44	-1.71131262
16756960	NM_013300	FAM216A	-1.714954143
17045555	NM_000712	BLVRA	-1.715176051
16698556	NM_173854	SLC41A1	-1.716075865
16998007	NM_001297765	CETN3	-1.716817476
16873268	NM_000400	ERCC2	-1.717115003
17068093	NM_001122824	TACC1	-1.717305448
17015277	NM_001286132	RPP40	-1.717325287
17009289	NM_001015051	RUNX2	-1.718214319
16661192	NM_005517	HMGN2	-1.719679843
17015240	NM_001166010	ECI2	-1.722025691
17096141	XR_930142	LOC105376158	-1.722753952
16660624	NM_001009999	KDM1A	-1.723231667
16954251	NM_005879	TRAIP	-1.725363092
16666509	NM_006417	IFI44	-1.727361452
16884280	NR_046110	LINC01123	-1.727940252
17014091	NM_016224	SNX9	-1.728714949
17010749	NM_138409	MRAP2	-1.733142165
16693491	NM_001024210	S100A13	-1.734612407
16685281	NM_031280	MRPS15	-1.735117464
17100597	NR_122035	ARRDC1-AS1	-1.73616011
16776961	NR_044993	GAS6-AS2	-1.736705743
16839352	NM_001080779	MYO1C	-1.738146879
16957396	NM_199511	CCDC80	-1.738697155
16997393	NM_032367	ZBED3	-1.739681658

17117537	XR_171880	LOC100131262	-1.740240461
16943681	NM_014648	DZIP3	-1.741978317
16666738	NM_001554	CYR61	-1.743746112
16768941	NM_153687	IKBIP	-1.744531926
17112426	NM_030763	HMGN5	-1.745781898
16736422	NM_138421	SAAL1	-1.747807947
16677473	NM_016121	KCTD3	-1.748296648
17007910	NM_002754	MAPK13	-1.749735275
16849496	NR_110848	LOC101928710	-1.749848475
16700058	NM_002221	ITPKB	-1.753584154
16881514	NM_015120	ALMS1	-1.75391642
16667760	NM_001400	S1PR1	-1.755339392
16853716	NM_005559	LAMA1	-1.757059849
16866275	NR_015380	A1BG-AS1	-1.757518652
16672097	NM_144772	APOA1BP	-1.757538955
16769362	NM_001031701	NT5DC3	-1.757632356
16730819	NM_000019	ACAT1	-1.757782619
16824470	NM_001313858	ARL6IP1	-1.758810438
16746906	NM_003213	TEAD4	-1.762272062
16799538	NM_001159508	IVD	-1.763180288
16882162	NM_031283	TCF7L1	-1.768778426
17089147	NM_001011703	MVB12B	-1.769207586
16728150	XR_913251	LOC105369364	-1.769698182
16732584	NM_003105	SORL1	-1.770630691
17087308	NM_001302884	TDRD7	-1.770712513
17024414	NR_002768	HYMAI	-1.77156369
16904588	NM_024753	TTC21B	-1.772140923
17044193	XR_252181	LOC101927841	-1.773275466
16777812	NM_001014380	KATNAL1	-1.774058192
16701606	NM_001004692	OR2T12	-1.774734647
16736726	NM_148893	SVIP	-1.774812558
17013728	NM_001242767	MTHFD1L	-1.775378544
16820992	XR_917451	LOC105371338	-1.777430726
16668333	NM_000850	GSTM4	-1.779016634
16833476	NM_024308	DHRS11	-1.780480539
16942525	NR_109992	SUCLG2-AS1	-1.781760385
16917504	NM_001282454	SNX5	-1.784071374
16939247	NM_001145464	EXOG	-1.784145573
17082982	NM_001190458	DOCK8	-1.784772265
16806561	XM_011508479	LOC102725021	-1.78555594
16895179	NM_001206802	TP53I3	-1.78672797
16918522	NM_080476	PIGU	-1.787314273
16753607	NM_001031679	MSRB3	-1.787384477
16669245	ENST00000369466	TTF2	-1.787917292
16829835	NM_001257328	ARRB2	-1.789243824

16722189	NM_021961	TEAD1	-1.79156038
16673359	NM_001198783	POU2F1	-1.791564519
16946016	NM_000532	PCCB	-1.796451517
16716093	NR_120661	LOC101929574	-1.797331677
17057983	NM_016139	CHCHD2	-1.79819565
16824132	NM_001270766	NTAN1	-1.799188901
16960911	NM_020169	LXN	-1.800236773
17112956	NM_001006684	TCEAL8	-1.800502996
16860140	NM_001256171	ZNF85	-1.801226989
16719233	NM_000274	OAT	-1.802230243
17110401	NM_001257291	SLC9A7	-1.802292704
16706832	XR_946158	LOC105378405	-1.803446551
16745281	NM_006500	MCAM	-1.804046677
16888143	NR_110204	LOC101927027	-1.805101548
16966304	NM_001112717	LIMCH1	-1.805527007
17008121	NM_003958	RNF8	-1.805556209
17087397	NM_018946	NANS	-1.806061057
16836457	NM_002876	RAD51C	-1.806257194
16750996	NM_001095	ASIC1	-1.809076396
16807139	NM_001220482	MEIS2	-1.809143275
16690388	NM_013386	SLC25A24	-1.809151635
16861563	NM_001013659	ZNF793	-1.809473526
16888367	NM_001271581	DNAJC10	-1.810970861
16659036	NM_018438	FBXO6	-1.811117315
16998952	NM_001017397	TRIM36	-1.811192639
16743091	NM_012193	FZD4	-1.813072564
16906285	NM_001271751	CALCRL	-1.813365824
16886105	NM_001024074	HNMT	-1.814476451
16979400	NM_001300823	PRDM5	-1.814769938
16818359	NM_001042454	TGFB1I1	-1.814857994
17087813	NM_001145313	FSD1L	-1.818186259
16793067	NM_001134999	FERMT2	-1.818530766
16841561	NM_000304	PMP22	-1.81900562
16681749	NM_001127325	MAD2L2	-1.819825349
16757636	NM_001109903	RNFT2	-1.820679101
16897313	NM_001305624	CALM2	-1.821150309
16743262	NM_001286139	SLC36A4	-1.822901574
16661589	NM_001048194	RCC1	-1.824051758
16968051	NM_001306147	11-Sep	-1.82641338
17067190	NM_001197293	DPYSL2	-1.826485119
16885516	NM_025029	MZT2B	-1.828507655
17052295	NM_001008749	RAB19	-1.828955534
16835934	NM_001258372	SPATA20	-1.830951192
16928115	NM_000854	GSTT2	-1.831056955
16874082	NM_001164773	BCAT2	-1.833745383

16753158	NR_027032	AGAP2-AS1	-1.835182238
17099248	ENST00000292035	MED27	-1.835572375
17061208	NR_026660	RPL19P12	-1.835669923
17055480	NM_020319	ANKMY2	-1.835852307
16688024	NM_001271999	DOCK7	-1.837626203
16802479	NM_015554	GLCE	-1.838033846
16809263	NM_001174116	DMXL2	-1.838713454
16793188	NR_036194	MIR4308	-1.842494101
16820989	XR_429768	LOC101927978	-1.843017794
16868130	NM_001165895	CD320	-1.843375524
17104995	NM_020932	MAGEE1	-1.850630235
16922222	NM_000874	IFNAR2	-1.85242269
16840788	NM_001166621	TRAPPC1	-1.852833616
16664156	NM_015112	MAST2	-1.855708364
17081829	NM_001080431	SLC45A4	-1.855755528
16665656	NM_018211	RAVER2	-1.864660954
16777460	NM_006437	PARP4	-1.866583438
16771570	NM_019034	RHOF	-1.868283425
17017126	NM_001243042	HLA-C	-1.868477684
16833171	NM_001033504	TMEM98	-1.876676525
16831306	NM_006041	HS3ST3B1	-1.879549182
17064235	NM_001164458	ACTR3C	-1.880026937
16849574	ENST00000586713	CEP295NL	-1.883409441
16997245	NM_015566	FAM169A	-1.900268571
16849556	NM_003255	TIMP2	-1.900958012
16707616	NM_001134658	SLC35G1	-1.902618969
16699932	NM_014698	TMEM63A	-1.902847574
17079436	NM_005836	HRSP12	-1.904888649
16771787	NM_032554	HCAR1	-1.907839755
16966026	NM_138389	FAM114A1	-1.911157487
16929920	NM_005318	H1F0	-1.913198634
17089844	NR_046240	LOC100506100	-1.913459457
17069905	NM_001011720	XKR9	-1.916468121
16969911	NM_001127493	ANK2	-1.918470614
17085829	NM_001242505	GDA	-1.918705557
16768543	NR_038263	SOCS2-AS1	-1.920031527
16852322	NM_001292039	MAPK4	-1.924112739
16936947	NM_001099952	ITPR1	-1.926167724
16820168	NM_001193522	FAM65A	-1.926519337
16959896	NM_001039547	GK5	-1.927436503
16689042	NM_032184	SYDE2	-1.928398661
16806920	NM_153613	LPCAT4	-1.931060458
16718414	NM_001244949	GPAM	-1.931265707
16948555	NR_038301	LINC00888	-1.931975323
16961331	NM_001105077	MECOM	-1.932828099

16732315	NM_032015	RNF26	-1.933775076
16850371	uc010wyn.1	ARL17A	-1.934382816
16783047	NM_001308097	G2E3	-1.935182999
16904551	NM_004482	GALNT3	-1.939914856
17001927	NM_001309443	SPARC	-1.942013639
16863493	NM_005184	CALM3	-1.942368145
16699081	XR_920756	LOC105372906	-1.94594374
16860175	NR_027130	ZNF738	-1.947779007
16994340	NM_138809	CMBL	-1.948485686
17006233	NM_001199119	TRIM39-RPP21	-1.950584728
16951247	NM_001195098	ANKRD28	-1.951716265
16763467	NR_037144	PLEKHA8P1	-1.952803339
16908154	NM_018441	PECR	-1.952925165
16755542	NM_001160	APAF1	-1.954717336
16800506	NM_003104	SORD	-1.95836091
17110404	NM_001257291	SLC9A7	-1.960325647
16989265	NM_001289984	JADE2	-1.964633221
17012047	NM_173674	DCBLD1	-1.967172311
17018292	NM_032340	UQCC2	-1.969018501
17005248	XM_011514327	E2F3	-1.970128867
16739435	NM_001286086	C11orf98	-1.977745332
17012963	NM_020340	ARFGEF3	-1.978024095
16923200	NM_018964	SLC37A1	-1.980013134
16768149	NM_005447	RASSF9	-1.982219417
16991345	NM_198321	GALNT10	-1.984153077
17078434	NM_000880	IL7	-1.986579689
16704107	NR_026827	LINC00839	-1.986694442
16755131	NM_001270467	SOCS2	-1.988407339
16930418	NM_000026	ADSL	-1.990816156
17010246	NM_001160130	KCNQ5	-1.991533848
16781315	NM_007368	RASA3	-1.993600958
16660976	NM_020451	SEPN1	-1.997963185
16715361	NM_000917	P4HA1	-1.998531068
17050224	NM_001008405	BCAP29	-2.000323495
17110670	NM_006875	PIM2	-2.001016874
16968735	NM_001165136	HERC6	-2.003339133
17077856	NR_002599	SNHG6	-2.003501144
16909303	NM_001100818	PID1	-2.010428925
16969578	NM_183075	CYP2U1	-2.024379823
16922920	NM_012105	BACE2	-2.024922463
16887097	NM_001172173	CSRNP3	-2.029198501
16833965	NM_001190918	THRA	-2.030084811
16947904	NM_001145097	SKIL	-2.030933967
16977520	NM_015697	COQ2	-2.034987595
17047985	NM_004194	ADAM22	-2.036464502

16997010	OTTHUMT0000037263	GTF2H2	-2.036723306
17059532	NM_018843	SLC25A40	-2.037900104
16842834	NM_004475	FLOT2	-2.037989568
16856350	NM_001303499	CNN2	-2.039845662
16799577	NM_130468	CHST14	-2.040779059
16946055	NM_001097599	SLC35G2	-2.047741113
16958356	NM_002213	ITGB5	-2.05056764
17088991	NR_038975	MIR181A2HG	-2.051046216
16887014	NM_012198	GCA	-2.051060432
16907303	NM_001044385	TMEM237	-2.055581633
16831381	NR_002211	MEIS3P1	-2.064658725
16915712	NM_016354	SLCO4A1	-2.065775295
16707184	NM_001031683	IFIT3	-2.070281098
16734762	NM_003141	TRIM21	-2.071209279
17067566	NM_001008710	RBPMS	-2.076038694
16981506	NM_001130688	HMGB2	-2.082240437
16718666	XM_011539805	ABLIM1	-2.08334726
16979875	NM_001300828	PCDH18	-2.083689051
17067314	NM_016240	SCARA3	-2.088190503
16988984	NM_001131027	PDLIM4	-2.088788857
16803185	NM_001284377	COMMD4	-2.090517326
16751554	NM_000224	KRT18	-2.091000393
16840982	NM_001256012	MYH10	-2.091894363
16844408	NM_152349	KRT222	-2.092416425
16842786	NM_001142624	RAB34	-2.098798019
16979213	NM_152402	TRAM1L1	-2.105043432
17000650	NM_001301738	TMEM173	-2.107130987
17109396	NM_001198719	RBBP7	-2.109279092
16906872	NM_001195144	ANKRD44	-2.11145378
16692614	NM_001123375	HIST2H3D	-2.113557458
16690638	NM_000849	GSTM3	-2.118524775
16756431	NM_152261	TMEM263	-2.120983406
17081039	NR_108049	CCAT1	-2.125609627
17092947	NM_001004125	TUSC1	-2.127240771
16863124	NM_001645	APOC1	-2.130044149
17052767	NM_001242773	TMEM139	-2.136081462
17069063	NM_001111097	LYN	-2.144835
17055216	NM_001136020	ICA1	-2.149269904
16711881	NM_001100912	BEND7	-2.149870858
16840599	NM_001185022	CLDN7	-2.150829752
17002396	NM_001303251	ZBED8	-2.162859574
16862333	NM_004596	SNRPA	-2.163209411
16970396	XM_011531680	BBS12	-2.181171687
16992761	NM_001308195	SIMC1	-2.195921782
16992265	NM_022897	RANBP17	-2.200843663

17048083	NM_001040665	STEAP2	-2.205358708
16690566	NM_001205228	SORT1	-2.210822704
17109367	NM_001144002	CTPS2	-2.214498469
17070426	NM_198584	CA13	-2.226591141
17090045	NR_120685	LINC01503	-2.227501907
16794177	NM_001102	ACTN1	-2.23853778
16914171	NM_002638	PI3	-2.245079708
16670359	NR_125392	RNVU1-20	-2.251224602
17096242	NM_153698	AAED1	-2.252436862
17106438	NM_144658	DOCK11	-2.266526978
16960271	NM_003071	HLTF	-2.267349303
16802106	NM_016395	HACD3	-2.268659354
16842128	AK124785	USP32P2	-2.271140041
16705089	NM_001080512	BICC1	-2.276893271
16674355	NM_001252511	SOAT1	-2.280246829
17112623	NM_001129896	SYTL4	-2.283088314
16754536	NM_001135805	SYT1	-2.287439099
16691923	NR_104073	RNVU1-4	-2.287750941
16691961	NR_104073	RNVU1-4	-2.287750941
17092045	NM_001282116	RFX3	-2.28894585
16932008	NM_001196	BID	-2.294839623
17056105	NM_006735	HOXA2	-2.306582136
16979050	NM_052864	TIFA	-2.308560167
16830173	NM_001195228	FAM64A	-2.317822358
16686832	NR_026878	FOXD2-AS1	-2.328202696
17073259	NM_001127213	LY6E	-2.337349272
16943763	NM_001243286	PVRL3	-2.377480212
16747287	NM_014865	NCAPD2	-2.377595571
17093743	NM_001301226	TPM2	-2.379508047
17010941	NM_001010853	PM20D2	-2.380310866
16705283	NR_120647	LINC01515	-2.381873289
17087413	NM_024642	GALNT12	-2.383282549
16979468	NM_198179	QRFPR	-2.383926903
16755878	NM_020244	CHPT1	-2.388569188
17012516	NM_001007102	L3MBTL3	-2.412061458
17051626	NM_001253900	MEST	-2.414910966
17005774	NR_026775	LINC00240	-2.416741778
16718592	NM_001001936	AFAP1L2	-2.423143761
17005542	NM_000410	HFE	-2.429063155
16811886	NM_138573	NRG4	-2.434063265
17012559	NM_004842	AKAP7	-2.434788854
16707503	NM_001013848	EXOC6	-2.434833859
16834711	NM_001171251	C17orf53	-2.440979197
16942732	ENST00000492922	LINC00960	-2.441407864
16971546	NM_001130067	TRIM2	-2.455119646

16906031	NM_001113397	ZNF385B	-2.459008406
16878731	NM_014600	EHD3	-2.460201814
17055978	NM_138811	C7orf31	-2.461719983
16984646	NM_015946	PELO	-2.494302891
16938656	NM_138410	CMTM7	-2.52213704
16906352	NM_000393	COL5A2	-2.524019985
17086708	NM_001135052	SYK	-2.525735094
17013507	NM_001030060	SAMD5	-2.527194437
16679020	NM_080738	EDARADD	-2.543026801
17113606	NM_015129	06-Sep	-2.54338524
16895278	NM_004036	ADCY3	-2.549474662
16819244	OTTHUMT0000043432	MT1CP	-2.549651385
16775434	NM_001306080	LMO7	-2.556346382
16690139	NM_001033025	EXTL2	-2.589469681
16870984	NM_138286	ZNF681	-2.601427198
16944156	NM_173570	ZDHHC23	-2.601523369
16946513	NM_001251845	TRPC1	-2.625787197
17103799	NM_018094	GSPT2	-2.627814309
16700911	NM_019891	ERO1B	-2.635329459
16904425	NM_001303422	GRB14	-2.642756189
17021323	NM_001080508	TBX18	-2.649940561
16664218	NM_005727	TSPAN1	-2.654242158
16986244	ENST00000610426	NSA2	-2.656358749
17015972	NM_198586	NHLRC1	-2.65821906
17114177	NR_026975	FIRRE	-2.666658966
16725742	NM_001281501	FADS2	-2.682807115
17010273	NR_120503	KCNQ5-IT1	-2.78062013
17102210	NM_001142386	PDK3	-2.791627922
17063394	NM_001164665	KIAA1549	-2.814255539
16753550	NM_015279	TBC1D30	-2.827878233
16905436	NR_040001	LINC01116	-2.831997516
16847249	NR_030411	MIR454	-2.83289409
16751973	NR_026656	LOC400043	-2.847975364
16879500	NM_172069	PLEKHH2	-2.879503349
16739132	NM_013402	FADS1	-2.886610927
16963428	NM_005929	MFI2	-2.886704302
16777756	NM_001135919	SLC46A3	-2.936891565
16769463	NM_032148	SLC41A2	-2.940571697
16978417	NM_001135146	SLC39A8	-2.968608137
16747257	XR_913809	LOC105369628	-2.993811388
16870828	NM_001256648	ZNF43	-3.007059331
17113980	NM_001282874	SMARCA1	-3.010799562
16980882	NM_001039580	MAP9	-3.07370065
16748496	ENST00000534843	APOLD1	-3.157946361
17055697	NM_001127370	CDCA7L	-3.166721513

16832104	NM_018242	SLC47A1	-3.16697029
16920762	NM_001336	CTSZ	-3.183175426
16842147	NR_036647	CCDC144B	-3.265076211
16999475	NM_001999	FBN2	-3.28411201
17005001	NM_001040280	CD83	-3.335153894
17088300	NM_133374	ZNF618	-3.338044837
16779766	NM_007249	KLF12	-3.394564896
16780322	NM_014934	DZIP1	-3.408459876
17056291	NM_001145513	SCRN1	-3.44974566
17010950	NM_001242809	ANKRD6	-3.475884154
16913341	NM_006097	MYL9	-3.633668143
16887062	NM_001040142	SCN2A	-3.636086868
16712076	NM_001010924	FAM171A1	-3.681277909
17024394	NM_001080951	PLAGL1	-3.688838598
16995500	NM_001127671	LIFR	-3.723819987
16798812	OTTHUMT0000043073	ARHGAP11B	-3.73673952
17010544	NM_031469	SH3BGRL2	-3.75142834
16858137	NM_000201	ICAM1	-3.969993559
16780271	NM_001105515	ABCC4	-3.972718766
17103185	NM_003254	TIMP1	-3.974903952
16701238	NM_001206729	AKT3	-4.021266638
16957884	NM_007085	FSTL1	-4.060272813
16977820	NM_016245	HSD17B11	-4.087305274
16684080	NM_002038	IFI6	-4.100424669
16836492	NM_018304	PRR11	-4.112455307
17062321	NM_001009571	CADPS2	-4.141337173
17066018	NM_016353	ZDHHC2	-4.179769351
17081027	NR_120364	CASC19	-4.192158701
16972553	NM_170710	WDR17	-4.364037
17000439	NM_001287582	CDC25C	-4.402226371
16979339	NM_001083	PDE5A	-4.427206498
16843098	NM_000386	BLMH	-4.517151602
16809659	NM_173814	PRTG	-4.587325671
16810543	NM_001029989	KIAA0101	-4.606486122
17062280	NM_005763	AASS	-4.620919638
16990257	NM_018936	PCDHB2	-4.654178899
16769419	NM_031302	GLT8D2	-4.94710093
16809650	NM_015617	PYGO1	-4.971392462
16677683	NM_017898	02-Mar	-5.035622032
16677425	NM_016343	CENPF	-5.302686044
16986913	NM_001126336	VCAN	-5.379094496
16692636	NM_175065	HIST2H2AB	-5.551547576
17114774	NR_030581	MIR891A	-5.597826089
17016503	NM_003533	HIST1H3I	-5.619103601
16801557	NM_004701	CCNB2	-5.656566714

16914315	NM_001281741	UBE2C	-5.671642769
16919022	NM_015474	SAMHD1	-5.681164458
16677133	NM_001033910	TRAF5	-5.830164809
16991859	NM_001142556	HMMR	-6.088611806
16880168	NR_002229	RPL23AP32	-6.621585332
16981444	NM_001286682	AADAT	-7.071452925
16844312	NM_001067	TOP2A	-7.118122033
16985599	NM_031966	CCNB1	-7.178803127
16799793	NM_001243142	NUSAP1	-7.610001123
16989636	NM_005733	KIF20A	-7.679416345
16912379	NM_012112	TPX2	-7.742306026
17066065	NM_001008539	SLC7A2	-8.241022405
16817017	NM_005030	PLK1	-9.043805718
16697544	NM_001206846	ASPM	-9.132458757
16775968	NM_001001715	FARP1	-9.247520969
16889966	NM_003812	ADAM23	-9.564105207
16978568	NM_001286734	CENPE	-10.08006707
16793225	NM_001146015	DLGAP5	-10.10067638
16719515	NM_001145966	MKI67	-10.13180984
16804631	NM_001308025	TICRR	-11.5241891
16965346	NM_022346	NCAPG	-15.65617628
17005865	NM_003521	HIST1H2BM	-20.04707711
16904324	NM_001291807	FAP	-26.52712953
