# Supplementary material for: Isolation and characterization of a new basal-like luminal progenitor in human breast tissue
Source: Stem Cell Res Ther. 2019 Aug 23;10:269. doi: 10.1186/s13287-019-1361-3 (PMC6708178; doi:10.1186/s13287-019-1361-3)
Supplement: Supplementary file 7 — Table S2. List of genes uniquely regulated by NR1. (PDF 139 kb) [file 13287_2019_1361_MOESM7_ESM.pdf]

List of genes uniquely regulated by NR1			
ID	RefSeq	Gene.Symbol	FoldChange
16681313	XR_931876	LOC105376694	17.92129932
16736453	NM_001127380	SAA2	11.57745839
16747678	NM_001734	C1S	9.437796081
17002052	NM_032385	FAXDC2	8.276265918
16984365	NM_000163	GHR	6.427911206
16760691	NM_001733	C1R	6.303384493
16688210	NR_037443	MIR3671	5.455683682
17006949	NM_001710	CFB	5.097418015
16949572	NM_006580	CLDN16	4.90101309
17060287	NM_000777	CYP3A5	4.568519353
16918023	NM_001252675	ACSS1	4.504218039
16735317	NM_153444	OR5P2	4.496128702
16976029	NM_002253	KDR	4.342131245
17023716	NM_001291702	VNN3	4.193204913
16735303	NR_036678	LOC283299	4.161056852
17025267	NM_000636	SOD2	4.103903099
16731461	NM_006169	NNMT	3.948605763
16681304	NM_018948	ERRFI1	3.807559724
16777527	NR_026730	TPTE2P1	3.403934635
17094372	XR_929616	LOC105376050	3.342243087
17070242	XR_929077	LOC105375913	3.255110834
16777944	NM_001079691	N4BP2L1	3.156429073
16869713	NM_017506	OR7A5	3.056887234
16715434	NM_145170	CFAP70	3.021028931
16689352	NM_004120	GBP2	2.935392317
16897946	NM_018014	BCL11A	2.866340498
16735288	NM_198185	OVCH2	2.843372933
16735321	NM_153445	OR5P3	2.835434835
16856803	NM_015675	GADD45B	2.820856611
16816424	NM_001308157	SYT17	2.802103024
17109326	NM_020665	TMEM27	2.736414285
17081401	NM_001135242	NDRG1	2.725655697
17075731	NM_001831	CLU	2.693701521
16752397	NM_001005915	ERBB3	2.675719097
16997399	NR_003014	SNORA47	2.612962947
16806968	NM_001023567	GOLGA8B	2.606462915
17016760	NR_001590	IFITM4P	2.605800556
16760714	NM_001297640	C1RL	2.582520856
16938133	NM_054110	GALNT15	2.563112258
16806936	NM_181077	GOLGA8A	2.544784224
16990331	NM_018935	PCDHB15	2.538113643
16963420	NM_025163	PIGZ	2.522568303
17077331	NM_001114634	PLAG1	2.511234347

16821396	NR_036147	MIR3182	2.438267932
17118260	XR_927614	LOC84214	2.4141411
17074673	NM_001908	CTSB	2.401862117
16815411	NR_033904	LINC00921	2.38262736
16781573	NM_001097577	ANG	2.36992817
16990327	NR_001281	PCDHB18P	2.367558379
16875297	NM_001145303	TMC4	2.336604132
17113710	NM_001104544	TMEM255A	2.330000073
16918011	NM_020531	APMAP	2.322319818
16771797	NM_177551	HCAR2	2.319783229
16807454	NR_040058	RAD51-AS1	2.2997069
16974968	NM_001297592	SEL1L3	2.29404443
17018299	NM_001142883	IP6K3	2.253415471
16972993	NM_003265	TLR3	2.245432468
16979330	NR_037596	LINC01061	2.242207818
16681449	NR_029610	MIR34A	2.221083042
16870401	NM_001170938	ISYNA1	2.207770178
16916289	NM_080571	C20orf96	2.207005156
17112170	NR_028379	FTX	2.192433879
16820439	NM_001305203	ZFP90	2.184949598
17053850	NR_024476	PAXIP1-AS2	2.183884665
16845222	NM_001991	EZH1	2.179886973
16916623	NM_001271168	PCED1A	2.178970503
17046750	NR_108104	LINC01372	2.175258215
17110525	ENST00000428463	ZNF630	2.172811971
16990294	NM_018930	PCDHB10	2.162060159
16774303	NM_014059	RGCC	2.13197423
16685769	NM_001033081	MYCL	2.129566822
16986409	NM_001311313	F2R	2.125285512
16766093	NM_005419	STAT2	2.118084285
16766874	NM_178539	FAM19A2	2.090188903
16691969	XR_917207	LOC105371220	2.082153841
17016434	NR_003504	GUSBP2	2.079999719
17070273	NM_001105539	ZBTB10	2.071443782
16713760	NR_003277	HNRNPA1P33	2.066176262
16966074	NM_025132	WDR19	2.05935612
16733851	NM_021034	IFITM3	2.056142139
16936732	NM_001130921	RABL2B	2.055102
16698784	NR_029518	MIR29B2	2.048058134
17076861	NM_005195	CEBPD	2.040081328
16787236	NM_001040428	SPATA7	2.039360277
16745658	NM_001199922	SIAE	2.025320181
16662728	XR_947215	LOC105378662	2.022752773
16976502	ENST00000506175	YTHDC1	2.019138677
16985704	NM_001205254	OCLN	2.016518535

16662430	NM_017629	AGO4	2.015819784
17118262	XR_242295	LOC644794	2.009031243
16916221	XM_011529022	TCEA2	2.008594957
16840766	AK128353	LOC100128281	1.998706544
16917939	NM_000099	CST3	1.992574041
16663282	ENST00000492422	CCDC30	1.990507996
17086805	NR_026868	ANKRD19P	1.988117926
16673763	NR_002719	TOP1P1	1.983580113
16663358	NM_001017922	ERMAP	1.982480488
17060531	NM_178831	GATS	1.977183356
17061048	XM_011516338	POLR2J3	1.967749627
17094475	XR_958154	LOC105379818	1.94953042
17012699	ENST00000230050	RPS12	1.94226044
16964632	NM_001306090	EVC	1.936225074
16883194	uc021vll.1	LOC100506123	1.928768507
16978548	NM_020139	BDH2	1.926724103
16752233	NR_036476	TMEM198B	1.920262224
16830653	NM_001406	EFNB3	1.918687824
16805894	NR_027053	LOC646214	1.909017066
16812790	NR_003661	UBE2Q2P1	1.907205102
16804003	NR_111962	GOLGA6L17P	1.904017405
16888718	NM_144708	ANKAR	1.903234506
16667258	NM_001024948	FNBP1L	1.901955291
16673748	NM_002022	FMO4	1.899030838
16948177	NR_110826	KCCAT211	1.896233552
16875056	NM_001105549	ZNF83	1.893711642
17072601	NM_001282985	TRIB1	1.891241153
16778413	NR_003365	SUGT1P3	1.889415502
16785313	ENST00000556725	SYNE2	1.886863423
16870850	XR_919502	LOC105372327	1.880574334
16800028	NM_001301409	GANC	1.88018332
17110523	ENST00000428463	ZNF630	1.876451064
16880488	NM_152392	AHSA2	1.873570155
16812505	NM_001164465	GOLGA6L10	1.871169174
16954091	NM_032316	NICN1	1.870650448
16806870	NM_001042494	SLC12A6	1.864648029
16688235	NM_024763	WDR78	1.86180675
16686438	NM_007170	TESK2	1.860495198
16907784	NM_001307976	KANSL1L	1.858853835
16811359	NM_001012642	GRAMD2	1.855026761
16751209	NM_001243689	LETMD1	1.850053084
16996941	NR_027439	LOC100272216	1.849582945
16800980	NR_024490	GABPB1-AS1	1.845497777
17098504	NR_002449	SNORA65	1.839865112
16698782	NR_029832	MIR29C	1.838806919

17051191	XR_927951	LOC101928451	1.830697386
16860183	NM_001076678	ZNF493	1.82784449
16787482	NM_001102366	C14orf159	1.827595337
16869186	NM_000528	MAN2B1	1.827468662
16900605	XR_109916	LOC100506036	1.825443058
16957095	NM_001777	CD47	1.824414237
16818083	NM_152288	ORAI3	1.82273311
16802408	NM_015322	FEM1B	1.822408861
16951079	XR_431566	LOC102725086	1.821327043
16692846	NM_000396	CTSK	1.82081372
17070878	NM_001142301	TMEM67	1.820174372
16857608	NM_018083	ZNF358	1.819106489
16812560	NM_001164465	GOLGA6L10	1.818253475
17066102	XR_428319	LOC101930275	1.816225496
16777624	NR_120429	LINC00412	1.815336083
16687273	NM_001198961	ECHDC2	1.81524381
16699775	XR_921154	GTF2IP20	1.814845414
17004491	NR_126016	LYRM4-AS1	1.812419184
16875760	NM_001190764	TMEM238	1.811958609
16662234	NM_152493	ZNF362	1.811728365
16913811	NM_001301860	LPIN3	1.810309873
16989325	NM_001745	CAMLG	1.810293143
16670599	NM_001288607	ADAMTSL4	1.80852474
16665621	NM_001293274	CACHD1	1.808470419
16686771	XR_947301	LOC105378701	1.80625302
16805495	NM_000875	IGF1R	1.802313525
16849744	NR_031652	MIR1250	1.801272769
16798744	OTTHUMT00000430715	GOLGA8Q	1.800594519
16798609	NR_037599	PDCD6IPP2	1.800078721
17102129	NM_002970	SAT1	1.795144527
16737669	NM_001101802	PHF21A	1.793245901
16996956	XM_011546977	NAIP	1.792989037
16965710	XM_011513851	TBC1D19	1.785836497
16798290	AF400494	SNRPN	1.778597422
16903537	NM_004688	NMI	1.776445383
17074601	NR_030328	MIR598	1.774804357
16663286	ENST00000462063	CCDC30	1.772083601
17114934	NM_001184808	CD99L2	1.771530945
16997046	NR_033968	GUSBP9	1.77085162
17090660	NM_018956	C9orf9	1.768194117
17067284	NM_001256482	EPHX2	1.767054659
16704454	XR_252745	LOC101927699	1.766715822
16676314	NM_001204171	MDM4	1.766695413
16922183	NR_024622	C21orf62-AS1	1.766650512
16838581	NM_001256071	RNF213	1.760782445

16986632	NM_153610	CMYA5	1.760766172
16879067	NM_016441	CRIM1	1.759460753
17107753	NM_001301228	HMGB3	1.759310346
16846915	NM_005082	TRIM25	1.753730019
16720049	NM_025092	ATHL1	1.749917208
16738544	NM_004223	UBE2L6	1.747311307
17005655	NM_001145008	BTN3A1	1.746661447
16912224	XR_920017	LOC105372582	1.745777865
16689969	NM_000110	DPYD	1.745660894
16790636	NM_001130706	C14orf93	1.745064063
16670377	NM_001034077	HIST2H4B	1.743907276
16692626	NM_001034077	HIST2H4B	1.743907276
16977925	NM_001015045	FAM13A	1.740694871
17058152	NM_001007253	ERV3-1	1.736440929
16914264	NM_006103	WFDC2	1.733939226
16786167	NM_000021	PSEN1	1.732813835
16733883	NM_001012302	ANO9	1.732025295
16982775	NM_001294337	C5orf38	1.729426059
16771801	NM_006018	HCAR3	1.72745724
16717783	NM_001142434	MGEA5	1.7238051
16779283	NM_001031719	DHRS12	1.718599444
16913869	NM_015478	L3MBTL1	1.715084907
16953454	NM_033199	UCN2	1.713409509
16833060	NM_052888	LRRC37B	1.712522963
16812544	NM_001164465	GOLGA6L10	1.708440496
17082453	NM_017570	OPLAH	1.705289484
17046135	NM_005228	EGFR	1.704718271
16962034	NM_015078	MCF2L2	1.704182687
16900724	uc002sxv.4	LOC100506123	1.703568548
16967794	NM_001511	CXCL1	1.702258338
16936648	NM_001145134	CPT1B	1.70081945
16862491	NM_001042595	TMEM91	1.700316519
16868462	NM_001077624	ZNF846	1.700296876
16905216	NM_001033045	GPR155	1.699582036
16897667	NM_001135598	CLHC1	1.699448528
17094378	NR_126048	LOC102724238	1.698125792
16977409	NR_030301	MIR575	1.693733235
17098911	NM_006336	ZER1	1.692430589
17118292	XM_005271793	LOC101060341	1.692106062
16675009	NM_001105518	SWT1	1.690535134
16723998	NM_001127219	ACCS	1.687752474
17012711	NR_002436	SNORA33	1.685095084
16848453	NM_001159770	SLC39A11	1.68469022
16774162	NM_001012754	NHLRC3	1.683884673
16898728	NM_022037	TIA1	1.682500189

16701156	NR_029401	LINC01347	1.681241142
16695284	NM_015726	DCAF8	1.679998564
16938724	NM_001171713	FBXL2	1.679928696
16766767	NM_005730	CTDSP2	1.679009042
17008515	NM_013397	PRICKLE4	1.67647
17118160	XR_108650	LOC401261	1.675366425
16866389	XM_006722953	LOC101928344	1.67362155
17013554	NM_005715	UST	1.670812683
16836538	NM_001166301	DHX40	1.66946209
17009659	AK095315	FBXO9	1.668320727
16664359	NM_000779	CYP4B1	1.668251345
16782687	NM_006084	IRF9	1.668208946
16914741	NR_002433	SNORD12C	1.668039362
16945137	NM_032548	ABTB1	1.667688686
16853028	NR_038340	LOC100505817	1.665474579
16953458	NM_000094	COL7A1	1.664897469
16739165	NM_021727	FADS3	1.664424389
16695508	NM_001113205	TSTD1	1.663839954
16924068	NM_001242864	PRMT2	1.663363331
17020480	NR_132999	GUSBP4	1.662475792
17118033	NR_103825	LOC729218	1.662414335
16824865	OTTHUMT00000403182	NPIPB5	1.662153168
16996971	AK289965	GUSBP3	1.661700064
17010813	NM_001010868	C6orf163	1.661201025
16785869	NM_001039465	SRSF5	1.660709809
16825602	NM_001282062	DOC2A	1.658443657
16802251	NM_001145102	SMAD3	1.656341323
16931321	NR_027033	MIRLET7BHG	1.655308365
17013722	XM_011536286	LOC105378056	1.655052139
17044758	NM_152793	MTURN	1.654398367
16996969	AK124130	GUSBP3	1.654130816
16877133	NM_001282710	PQLC3	1.652511143
16679952	NR_033908	LOC100288069	1.650294309
16844137	NM_001184906	FBXL20	1.650179923
16816897	OTTHUMT00000402426	NPIPB4	1.649825377
16957089	AK057598	LOC151657	1.649341336
16679899	XR_426576	LOC100134822	1.647128754
16955645	NM_001166243	FHIT	1.645979841
16976201	NM_032313	NOA1	1.644694922
16858263	NM_031209	QTRT1	1.64442514
16812997	NM_001144074	DET1	1.644223783
16985751	XM_011546970	LOC100653061	1.643346456
16942648	NM_001080393	GXYLT2	1.64294403
16886331	NM_018328	MBD5	1.642689718
16997316	NM_001130105	COL4A3BP	1.641623551

16683423	NM_000191	HMGCL	1.641153292
17004313	NM_001302777	BPHL	1.640429205
16687208	NM_001009881	ZCCHC11	1.639743322
16837065	NM_138363	CEP95	1.639413746
16928182	NM_019601	SUSD2	1.638868387
17022362	NM_001199933	SESN1	1.638769938
17060503	NM_024637	GAL3ST4	1.636866504
16712292	NM_014241	HACD1	1.636764394
17011450	NM_001455	FOXO3	1.636201014
16918683	NR_102705	MMP24-AS1	1.63375313
16772942	NM_001190964	ZMYM2	1.633530434
16738137	NM_024783	AGBL2	1.633164372
16785155	NM_015180	SYNE2	1.632477755
17000756	NM_006051	APBB3	1.631572768
17058978	NM_017439	GSAP	1.630924503
16900737	NM_025190	ANKRD36B	1.630212462
16717102	NM_012083	FRAT2	1.629150629
16869684	NM_001271052	ADGRE2	1.628913506
16956194	NR_031697	MIR1284	1.628051872
16920338	NM_006045	ATP9A	1.627893893
16996946	NR_027386	GUSBP3	1.627453888
16706023	NM_173348	FAM149B1	1.626055688
17068642	NM_152419	HGSNAT	1.625338262
17118220	ENST00000622469	ZNRD1-AS1	1.625101693
16854673	NM_001112734	ZSCAN30	1.624091971
16825333	NM_000086	CLN3	1.622452978
16835711	NM_001199898	PDK2	1.621276325
16871765	NM_152279	ZNF585B	1.620961696
16840769	NR_024349	LOC284023	1.620736999
16668622	NM_001025197	CHI3L2	1.620021919
16888963	NM_001031716	NABP1	1.619591526
17004125	NM_032765	TRIM52	1.618948021
16798067	NR_022008	PWAR5	1.618899395
16856604	NM_138393	REEP6	1.618170171
17016579	NM_001012455	ZSCAN23	1.617695417
16888183	NR_038271	TTN-AS1	1.616234648
16717394	XM_011540242	SLC25A28	1.614648352
17118258	XR_928282	LOC101060128	1.614629699
17020174	NM_001197115	GCLC	1.614458101
17096091	NM_000264	PTCH1	1.614293981
17045425	NM_001193311	SUGCT	1.613592929
17051194	NR_024368	LINC01000	1.613346887
16803973	NM_001291420	GOLGA6L9	1.612135864
16735729	XM_011520536	LOC105376544	1.612031572
16677104	NM_001136223	RCOR3	1.611041137

16946341	NM_001037172	PXYLP1	1.609872761
17118384	XM_011519130	C9orf3	1.609177346
16815194	NR_024492	FLJ42627	1.607954593
17117460	XR_921456	LOC105373249	1.606992653
16866838	NM_017797	BTBD2	1.606792167
16918859	NM_001242599	RBM39	1.606105505
16661351	NM_001276252	WDTC1	1.606005314
16723961	NR_026952	SEC14L1P1	1.605879157
16979336	XR_939133	LOC101926918	1.60566397
16803926	NR_004847	UBE2Q2P2	1.605612032
16894979	NM_022460	HS1BP3	1.603917571
16815461	NM_015041	CLUAP1	1.60309879
16990401	NM_001142603	KIAA0141	1.602983972
16731605	NM_001040455	SIDT2	1.601255286
16830573	NR_002918	SNORA48	1.601066613
16991604	NM_017872	THG1L	1.600903855
16693872	NM_020524	PBXIP1	1.600522916
16824366	NM_001282507	NPIPA7	1.598545712
16800933	NM_001144955	DTWD1	1.597530343
17010539	XM_011536282	LOC105377866	1.595700616
17043177	X58060	SNORD13P2	1.593331731
16849268	NM_134268	CYGB	1.592621383
17117631	AK130927	LOC100128075	1.591837792
17045071	XM_011515675	FLJ20712	1.591543585
16688164	NM_002227	JAK1	1.590073366
16696895	NR_037642	LHX4-AS1	1.589790505
16957041	NR_028303	LINC00882	1.589566456
17057931	DQ599768	LINC01061	1.588905511
16963845	NM_000203	IDUA	1.58825952
16863968	NR_004383	SNAR-G1	1.588237502
16950269	NM_001164674	SUMF1	1.586686016
16845126	NM_003150	STAT3	1.586631027
16669504	XM_011544006	LOC105379272	1.586436746
16677595	XR_920789	LOC101929713	1.585374123
17070665	NM_001359	DECR1	1.584476944
16824463	NM_001019	RPS15A	1.584301229
16866282	NM_001009	RPS5	1.584103574
16822249	XR_253606	LOC101930131	1.584019395
16993655	NR_028325	LOC100132062	1.579932949
16965680	NM_001292054	TBC1D19	1.579848992
16949611	ENST00000476929	IL1RAP	1.576691056
16703289	AK022045	PRINS	1.576680128
16805230	NM_001042572	CHD2	1.576669199
16879540	NM_001193464	DYNC2LI1	1.576257607
16688937	NM_004388	CTBS	1.573506713

16827248	NM_003789	TRADD	1.572797936
16828367	NM_030581	WDR59	1.572681654
16719587	NR_036179	MIR4297	1.570934832
16960801	NR_003284	PA2G4P4	1.570923943
17052749	NM_001143679	GSTK1	1.570285262
16735436	XM_006718397	TRIM66	1.56987171
16694506	NM_001256604	GLMP	1.569730256
16735152	NM_000391	TPP1	1.569327728
16904039	NM_001289975	BAZ2B	1.569284218
16822256	XR_917627	LOC101059936	1.568425134
16699780	NR_132119	GTF2IP20	1.567019718
16660863	NM_013943	CLIC4	1.566813358
16877543	NR_110235	LOC101928222	1.56617273
16663242	NM_001080850	CCDC30	1.566093122
16729126	NR_000025	SNORD15B	1.56522132
17014846	NM_001278531	ERMARD	1.564086173
17101616	NM_001195328	RAB9A	1.564028353
16970258	NM_015312	KIAA1109	1.563760964
17088790	XM_011518526	OR1J2	1.562421095
16698786	XR_920672	C1orf132	1.562016832
16793438	NR_029434	PSMA3-AS1	1.561782263
16966911	NM_018475	TMEM165	1.561457533
16858696	NM_017682	BEST2	1.561403418
16835356	NM_001278197	CDK5RAP3	1.561320446
16875145	NM_001102603	ZNF160	1.559719574
16911106	NR_029519	MIR103A2	1.559218738
16852069	XR_935236	KIAA1328	1.558037546
16850923	NM_006868	RAB31	1.557044309
16906835	NM_024989	PGAP1	1.556778114
16657450	NR_028322	LOC100132287	1.556123612
16881350	BC044944	ZNF638-IT1	1.555627525
16850656	NR_024101	DLGAP1-AS1	1.555566423
16798895	NM_001282494	GOLGA8N	1.555393915
16679892	XR_426570	LOC100996442	1.555354385
16679945	uc002khh.3	LOC100133331	1.554754364
16814693	NM_032520	GNPTG	1.554700481
16834733	NM_001076674	TMUB2	1.554502927
17067231	NM_004103	PTK2B	1.554298216
17017979	NM_000593	TAP1	1.553734501
16971867	NM_018342	TMEM144	1.553558606
17118152	AY358807	QIQN5815	1.553361197
16921664	NM_001207063	CXADR	1.553145871
16778294	NR_047500	LINC00571	1.551374149
17105687	XR_921548	LOC102724252	1.550875992
17023180	NM_152730	TBC1D32	1.550431729

16713428	NM_001305033	ZNF33B	1.550356503
16957479	NM_001164496	CFAP44	1.549597287
16970068	NR_003584	SNHG8	1.549325206
16787902	NM_001085	SERPINA3	1.549318047
16827700	NR_003610	PDXDC2P	1.548910016
16847897	NM_001303255	LRRC37A3	1.548455583
16874375	NM_001171937	FUZ	1.548283863
17086074	NM_001018037	VPS13A	1.545917504
17071495	NR_002182	NACAP1	1.545746065
17045032	NM_001033604	BBS9	1.545699638
16720803	NM_001042780	TNNT3	1.545221154
16877667	NM_004116	FKBP1B	1.545046223
16817283	NM_015202	KIAA0556	1.544378812
16881031	NM_018153	ANTXR1	1.544057701
16806702	NM_001277308	GOLGA8O	1.543850798
16868158	NR_038237	RAB11B-AS1	1.543394283
16774515	NR_024458	TPT1-AS1	1.543084072
16816287	NR_036447	PKD1P1	1.542916513
16704542	ENST00000476514	FAM21A	1.541473406
16879205	NM_001170791	RMDN2	1.540999792
16829722	NR_033795	OR1D4	1.54026651
16679517	NM_001130957	C1orf101	1.53967231
16863884	NM_006184	NUCB1	1.539362847
16808308	ENST00000437065	PPIP5K1	1.538481043
16830302	NM_000018	ACADVL	1.538228683
17047384	NR_033322	NSUN5P1	1.537123767
17059776	NM_001303496	SAMD9L	1.536623086
16933667	NM_001202502	NIPSNAP1	1.536530779
16819666	NM_001142302	CCDC113	1.536119018
16759676	NM_015394	ZNF10	1.535639952
16882819	NR_027714	ACTR3BP2	1.535629308
17105665	NR_038988	LINC00630	1.535004977
17083900	NM_001010887	ACER2	1.534841841
17097880	NM_012164	FBXW2	1.534026423
16781732	NM_001146683	TMEM253	1.533664942
16918137	NM_001290261	ZNF337	1.533551554
17003781	NM_001017987	C5orf45	1.533030783
17018215	NM_003190	TAPBP	1.532998905
16917892	NM_001283018	NAPB	1.532616419
16824400	XM_011546693	NPIPA7	1.532577468
17009054	NM_001291969	POLH	1.532209247
16873160	NM_001083335	ZNF112	1.532180926
17021922	NM_015491	PNISR	1.532011011
16891176	NM_001042410	ANKZF1	1.531572152
16706068	NR_132103	PPP3CB-AS1	1.531236014

16766185	NM_001280796	GLS2	1.530712493
17110763	NM_007213	PRAF2	1.528863918
16933030	NR_024448	GUSBP11	1.528482463
16895871	NM_015662	IFT172	1.52825293
16667346	NM_001114106	SLC44A3	1.528189373
16764238	NM_001300750	LMBR1L	1.527338671
16808326	uc001zsm.2	PPIP5K1	1.525095938
16939920	NM_001024855	ZNF197	1.524634401
16704920	NM_001005751	FAM21A	1.524504068
16919295	NM_032221	CHD6	1.522838905
17093031	NM_024761	MOB3B	1.522726317
16875508	NR_126418	LENG8-AS1	1.522191638
17082336	NM_178564	NRBP2	1.521091212
17023677	NM_003569	STX7	1.520813594
16687487	NM_001305043	TMEM59	1.52034984
17108943	NM_001142389	PNPLA4	1.520167188
16961989	NM_001293273	MCCC1	1.51960883
16864129	NM_001204502	FLT3LG	1.519391161
16908604	NM_015680	CNPPD1	1.519222664
16847296	NR_002924	TBC1D3P1-DHX40P1	1.517878872
16944695	NM_017554	PARP14	1.51776665
16727462	NM_024649	BBS1	1.516487213
16917567	NM_001099407	DZANK1	1.515369903
16849681	NM_000199	SGSH	1.515324388
16704397	NM_001169106	FAM21C	1.515068826
16959925	NM_001282857	XRN1	1.514047009
16989719	NM_001271803	REEP2	1.513312567
16751570	NM_001300821	EIF4B	1.512753232
16977781	OTTHUMT00000253045	C4orf36	1.51238628
17064150	NM_152411	ZNF786	1.511624703
16681735	NM_012168	FBXO2	1.511027587
16840723	NM_133491	SAT2	1.509579427
16953597	NM_001040454	SLC26A6	1.509251603
16875026	NM_178523	ZNF616	1.50923068
16769941	ENST00000537236	MMAB	1.509094691
16670479	NM_001300838	CIART	1.508397502
16866574	NM_001100122	SBNO2	1.507063285
16824867	NR_027155	SMG1P3	1.50674297
16867837	NM_001080452	GPR108	1.505653712
16818980	NM_005611	RBL2	1.505246747
16667801	NM_017619	RNPC3	1.504867709
17012709	NR_002435	SNORD100	1.504377534
17055082	NM_006956	ZNF12	1.504235031
17010584	NM_000056	BCKDHB	1.503981339
17096188	NM_001286990	SLC35D2	1.503915317

16665373	NM_176877	INADL	1.503505348
16699862	NR_039896	MIR4742	1.503352507
16733985	NR_038262	MIR210HG	1.502758659
16803966	NR_004847	UBE2Q2P2	1.502671859
16867715	NM_024103	SLC25A23	1.502022752
16713655	NM_001276343	AGAP4	1.501162337
16876182	NM_014453	CHMP2A	1.500860615
16789955	NR_002312	RPPH1	1.500101375
17097340	NM_021218	INIP	-1.500434145
16934804	NM_014550	CARD10	-1.500628295
16892485	NM_001190266	ATG16L1	-1.501086034
16908711	NM_001278552	TUBA4A	-1.501269862
17093920	NM_001128227	GNE	-1.501363519
16879118	NM_001278505	GPATCH11	-1.501776373
16940827	NM_203370	FAM212A	-1.501811072
16767136	ENST00000266604	LLPH	-1.502008871
16657514	NR_047519	LINC01128	-1.502217108
17005582	BC056264	HIST1H2BF	-1.502935747
16829580	NM_002945	RPA1	-1.503234414
16872166	NM_178820	FBXO27	-1.504221129
16849992	NM_001184917	PCYT2	-1.504686916
17000746	NM_001035235	SRA1	-1.50510764
16710271	NM_001007793	BUB3	-1.505518045
16974873	NM_001358	DHX15	-1.505608488
16957738	NM_003778	B4GALT4	-1.506572395
16914844	NM_001278618	PTPN1	-1.506830005
16819430	NM_152727	CPNE2	-1.507122481
16918132	NM_152667	NANP	-1.507296601
16953735	NM_004157	PRKAR2A	-1.507780759
16663014	NM_005857	ZMPSTE24	-1.508376592
17107907	NM_001129765	NSDHL	-1.508805319
16825097	XM_011545856	NDUFAB1	-1.508885501
16898801	NR_046798	TGFA-IT1	-1.509596866
16811847	NM_018285	IMP3	-1.509666626
16980716	NM_152680	TMEM154	-1.510224821
16668550	NM_001201545	RBM15	-1.510315547
16994329	NM_001258388	FAM173B	-1.510371381
17081447	NM_003033	ST3GAL1	-1.510462116
17092918	NM_001171195	ELAVL2	-1.510751806
17115636	NM_000402	G6PD	-1.512284947
16949980	NM_001105573	FBXO45	-1.512602945
16943254	NM_001167924	CMSS1	-1.513469918
16865737	NM_001012478	U2AF2	-1.513574827
16948278	NM_004301	ACTL6A	-1.513585319
17005593	NM_003532	HIST1H3E	-1.514904309

16807108	XR_916206	LOC105370767	-1.515296379
17016379	ENST00000314332	HIST1H2BC	-1.515383908
16781136	NM_001286277	TUBGCP3	-1.516410131
16664912	NM_001256409	LRRC42	-1.517272274
16745525	NM_006597	HSPA8	-1.517538726
16702071	NM_017782	FAM208B	-1.51760184
16846476	NM_001278784	SLC35B1	-1.517643918
16838847	NM_199287	CCDC137	-1.519945927
16896481	NM_005760	CEBPZ	-1.520799539
16994045	XM_011514079	MRPL36	-1.520866303
16947235	NM_003875	GMPS	-1.521906787
16899874	NR_039939	MIR4779	-1.522367499
17016966	NM_014641	MDC1	-1.52282835
16706128	NM_203298	CHCHD1	-1.523035954
17058484	NM_001197244	BCL7B	-1.523595572
16785631	NM_004094	EIF2S1	-1.525254514
17050497	NM_001166345	MDFIC	-1.52591366
16990920	NM_152407	GRPEL2	-1.526178104
17092020	NM_014878	PUM3	-1.527328085
16995029	XR_925901	LOC105374715	-1.528274117
16828499	NM_012091	ADAT1	-1.528754417
17090748	NM_001278928	SURF2	-1.529072345
16973247	NM_004477	FRG1	-1.529273734
16991018	NM_014983	HMGXB3	-1.529870991
17023308	NM_016063	HDDC2	-1.530111373
16663773	NM_001039589	DPH2	-1.530171474
16871613	NM_006233	POLR2I	-1.530199758
16765963	NM_173595	ANKRD52	-1.530935321
16986895	NM_003401	XRCC4	-1.532690784
16738429	NM_003146	SSRP1	-1.532885566
17049573	NM_001128852	SRRT	-1.533147676
16675889	NM_006335	TIMM17A	-1.533211439
17056461	NM_001130710	LSM5	-1.533381487
16963358	NM_152617	RNF168	-1.533767707
17114470	XM_011531238	CT45A10	-1.534076045
17018274	NM_001188	BAK1	-1.534643265
16902865	NM_001009993	FAM168B	-1.534976604
16833698	NM_002795	PSMB3	-1.535703819
16963320	NM_001204897	TM4SF19	-1.535774785
16761393	NM_001300739	MAGOHB	-1.536708296
17016946	NM_001134870	PPP1R18	-1.538061652
17074571	NM_001284356	PINX1	-1.538111404
16669067	NM_001172411	VANGL1	-1.538409951
17045656	NM_006555	YKT6	-1.539167242
16723353	NM_001077242	DEPDC7	-1.539334394

16996194	NM_021147	CCNO	-1.539576263
17096457	NM_003389	CORO2A	-1.539697212
16836156	NM_016001	UTP18	-1.540572595
16848953	NM_033452	TRIM47	-1.5407221
16811638	NM_001146029	SEMA7A	-1.540754139
17010876	NM_001197259	ORC3	-1.542727585
16869372	NM_004461	FARSA	-1.543643923
16834351	NM_001042529	COASY	-1.543658189
16993027	NM_001271828	PRELID1	-1.544853465
16881153	NM_006196	PCBP1	-1.546110394
16976390	NM_018227	UBA6	-1.546281873
16790556	NM_001039619	PRMT5	-1.546556993
16705474	NM_001256910	DDX21	-1.546635608
16876191	NM_003969	UBE2M	-1.547836766
17078480	NM_014018	MRPS28	-1.547851071
16740969	NM_001166212	CLCF1	-1.548373298
16924602	NM_006988	ADAMTS1	-1.548970856
16749423	NM_001248002	ARNTL2	-1.549378902
17042936	NM_002452	NUDT1	-1.550499793
16803059	NM_001127190	CSK	-1.550868826
17000793	NM_000591	CD14	-1.551291709
16659605	NM_024329	EFHD2	-1.551714707
16976158	NM_002703	PPAT	-1.552331488
16993173	NR_003615	LOC728554	-1.553838611
16969093	NM_001100426	RAP1GDS1	-1.55471485
16877946	NM_017877	SLC35F6	-1.557807174
16958506	NM_022776	OSBPL11	-1.557936754
16724178	NM_001145265	SLC35C1	-1.560188128
16829690	NM_001100398	RAP1GAP2	-1.560790246
16767837	NM_001300965	CSRP2	-1.56165958
17047707	NM_001127357	PHTF2	-1.561825566
16924207	NM_006948	HSPA13	-1.562081796
16896836	NM_001112800	SLC8A1	-1.563139642
16989293	NM_001252231	SEC24A	-1.563327458
16958812	NM_003707	RUVBL1	-1.564078946
16837689	NM_001303265	ICT1	-1.564725948
16728518	NM_001099653	FAM86C1	-1.568265694
16950999	NM_001098502	CHCHD4	-1.569218954
16889346	NM_001207067	BZW1	-1.56982093
16717359	NM_002079	GOT1	-1.571806184
17064724	NM_007349	PAXIP1	-1.572162126
16885842	NM_001508	GPR39	-1.572183921
16872163	ENST00000598394	FBXO27	-1.573884858
16726925	NM_001303024	SSSCA1	-1.574499537
17007377	NM_001077516	SLC39A7	-1.575675007

16685246	NM_032881	LSM10	-1.576250324
17072552	NM_003129	SQLE	-1.576272175
16951797	NM_001278182	EOMES	-1.576304953
16814295	XM_011522619	CAPN15	-1.577179285
16969651	XR_939060	LOC105377359	-1.577962952
16821807	NM_001294340	ZC3H18	-1.578874682
17054460	NM_001134340	PSMG3	-1.578907514
17076063	NM_000637	GSR	-1.57895494
16904410	NM_018086	FIGN	-1.579046146
17085975	NM_012383	OSTF1	-1.579513207
16882416	NM_016622	MRPL35	-1.579557001
16823719	NM_001289029	EEF2KMT	-1.580133735
16759604	NM_001170543	PGAM5	-1.580590162
16862145	NM_006503	PSMC4	-1.580816598
17063311	NM_001253775	CREB3L2	-1.581715359
16746992	NM_001759	CCND2	-1.583543684
16701748	NM_012341	GTPBP4	-1.584879696
16819469	NM_012106	ARL2BP	-1.585403427
16996201	NM_019030	DHX29	-1.587111332
16794347	NM_001284230	MAP3K9	-1.589489332
17093570	NM_007126	VCP	-1.5897501
17011694	NM_001289111	RPF2	-1.592069519
16833263	NM_002311	LIG3	-1.59487129
16766283	NM_001282601	PTGES3	-1.595416754
16677913	NM_003676	DEGS1	-1.595545776
17057946	NM_004577	PSPH	-1.596629973
16829758	NM_001014764	EMC6	-1.597641079
16814504	NM_001271285	FAM173A	-1.597892109
16846954	NM_016070	MRPS23	-1.598124717
16872407	NM_001256440	C19orf47	-1.598527245
16994969	NM_001040446	MTMR12	-1.599192192
17104947	NM_006517	SLC16A2	-1.599321519
17100585	NM_138462	ZMYND19	-1.599484117
16809093	NM_002041	GABPB1	-1.600656049
16869017	NM_032377	ELOF1	-1.600744811
16722799	NM_001145166	PRMT3	-1.601281184
16668730	NM_007204	DDX20	-1.601329282
16708552	NM_001284388	NOLC1	-1.601625297
16945907	NM_021203	SRPRB	-1.6016438
16871546	NM_001173514	TYROBP	-1.604210358
16718553	NM_001271816	DCLRE1A	-1.605259645
16823938	NM_001303447	TXNDC11	-1.605582355
16830182	NM_032731	TXNDC17	-1.605893998
16721126	NM_001033	RRM1	-1.607130038
16979719	NM_001300897	SCLT1	-1.608333584

16948909	NM_001194946	EIF4G1	-1.609110424
17006392	XR_954446	LOC105379695	-1.611513938
16660685	NM_003198	TCEB3	-1.612176838
16702175	NM_001145443	PFKFB3	-1.612426427
16843179	NM_018405	COPRS	-1.612653698
16836404	NM_080677	DYNLL2	-1.612724494
16826755	ENST00000564376	PLLP	-1.615801528
16833204	NM_002982	CCL2	-1.618805886
17003291	NM_005451	PDLIM7	-1.622719155
16966548	NM_207330	NIPAL1	-1.622820389
16841885	NM_001199125	COPS3	-1.623720521
16773552	NR_002162	ATP5EP2	-1.627863803
16741075	NM_001271849	CDK2AP2	-1.629135573
17000208	NR_030583	VTRNA2-1	-1.629549677
16691436	NR_110786	LOC101929099	-1.62962498
16662313	NM_005268	GJB5	-1.629790658
16779119	NM_002267	KPNA3	-1.63070596
16969817	NM_152400	C4orf32	-1.632447581
16913737	NM_003286	TOP1	-1.633160598
16902603	NM_004805	POLR2D	-1.633790878
16994618	NM_012334	MYO10	-1.634081568
16670401	NM_003517	HIST2H2AC	-1.638641207
16822809	NM_001300900	MRPS34	-1.638743434
16806914	NM_018648	NOP10	-1.639300115
16891971	NM_001130849	CAB39	-1.642279863
17013784	NM_001286562	ARMT1	-1.642575859
16977476	NM_001115007	LIN54	-1.64690043
16873087	NM_002250	KCNN4	-1.64869363
16943433	NR_002941	PDCL3P4	-1.654708016
16957568	NM_001308445	NAA50	-1.655652613
16971139	NM_001040876	ABCE1	-1.657489809
16929817	NM_018957	SH3BP1	-1.657616191
16683925	NM_003047	SLC9A1	-1.658236752
16833934	NM_002809	PSMD3	-1.658240583
16707944	NM_015652	C10orf12	-1.659796841
17045542	NM_004760	STK17A	-1.66090551
17011893	NR_027338	TPI1P3	-1.661354559
16696614	NM_001162893	KIAA0040	-1.662226137
16970673	NM_001287437	JADE1	-1.662310632
17015889	NM_001278209	NUP153	-1.663151969
16754269	NM_013381	TRHDE	-1.663751538
16825468	NM_001031827	BOLA2	-1.664285951
16825683	NM_001031827	BOLA2	-1.664285951
16787669	NM_001098621	TMEM251	-1.666117329
16860017	NM_001300946	GATAD2A	-1.667087698

16894283	NM_138799	MBOAT2	-1.66849805
16916863	NM_001134337	RNF24	-1.668918303
16847260	NM_001015509	PTRH2	-1.67110996
16724997	NM_000614	CNTF	-1.677725474
16797213	NM_017955	CDCA4	-1.678004595
17049965	NM_001204453	PSMC2	-1.678737511
17003050	NM_001256539	NOP16	-1.67902068
17061881	NM_014705	DOCK4	-1.684020849
17020715	NR_026807	LINC00472	-1.684569558
16830146	NM_001258217	MIS12	-1.684771963
16778489	ENST00000411348	RNU6-57P	-1.685394902
17013251	NM_032860	LTV1	-1.686563534
16728778	NM_002564	P2RY2	-1.689266172
16907488	NM_203365	RAPH1	-1.690476546
16657730	NM_001029885	CPTP	-1.694324255
16872551	NM_000660	TGFB1	-1.695248381
16969229	NM_001306151	DAPP1	-1.697980629
17081002	NM_174911	FAM84B	-1.698216035
16661117	NM_001281517	CEP85	-1.700933416
16904447	NM_001278458	COBLL1	-1.701676346
17103327	NM_006579	EBP	-1.703418984
16911754	NM_001172745	SEC23B	-1.705387988
17068064	NM_001199659	LETM2	-1.709589556
16723662	NM_014344	FJX1	-1.70980682
16840703	NM_004860	FXR2	-1.710988425
16851810	NM_001191324	RNF138	-1.712942432
16824004	NM_001130006	GSPT1	-1.714288591
16935069	NM_001278208	DMC1	-1.715172088
17078342	NM_001204857	TCEB1	-1.715528785
16817685	NM_024516	PAGR1	-1.717349095
16955016	NM_016329	SFMBT1	-1.718095225
17093122	NM_001199987	NDUFB6	-1.720426987
16965527	NM_018323	PI4K2B	-1.724410597
17014114	NM_001178088	SYNJ2	-1.726280216
16852025	NM_001281739	FHOD3	-1.726890572
16954697	NM_001947	DUSP7	-1.726970373
16840262	NM_002532	NUP88	-1.727768588
16740645	NM_001242481	EIF1AD	-1.729901628
16695572	NM_012394	PFDN2	-1.730445294
16917030	NM_001281467	TRMT6	-1.730781174
16859583	NM_024656	COLGALT1	-1.731385121
16880924	NM_020143	PNO1	-1.733118139
17093260	NM_022917	NOL6	-1.734925044
16901974	NM_000575	IL1A	-1.735430192
16980946	NM_016205	PDGFC	-1.73566277

16968378	NM_001292017	ENOPH1	-1.739464617
17045917	NM_001287426	UPP1	-1.741785137
16853042	NM_014177	TIMM21	-1.746007795
16975890	NM_001014446	OCIAD2	-1.748757203
17081706	XM_011516964	AGO2	-1.749476558
16835672	NM_002204	ITGA3	-1.75879012
16839184	NR_029406	RPL23AP87	-1.759306282
17069545	NM_015169	RRS1	-1.760176377
16816479	NM_001199022	CCP110	-1.761816089
16661141	NM_031286	SH3BGRL3	-1.763775165
16678907	NM_001012985	COA6	-1.765532445
16853523	NR_026659	LOC727896	-1.76599754
16898788	NM_001099691	TGFA	-1.766540306
17015771	NM_001031713	MCUR1	-1.76795718
17066224	NM_001174159	SH2D4A	-1.767989859
16690511	NM_005645	TAF13	-1.768038879
16685482	NM_024640	YRDC	-1.769293431
16926087	NM_001260474	WDR4	-1.770340252
16989241	NR_037434	MIR3661	-1.772075412
16778745	NM_001010875	SLC25A30	-1.773935228
16854046	NM_001271989	CEP76	-1.773951623
17056358	NM_001199815	GGCT	-1.774406637
16912052	NM_013248	NXT1	-1.77584213
16672265	NR_001560	CYCSP52	-1.779477058
16899764	NM_001042437	ST3GAL5	-1.782505671
17049700	NR_039797	MIR4653	-1.784067252
16984809	NM_001008397	GPX8	-1.7888718
17084237	NM_001314039	DNAJA1	-1.800819187
17016956	NM_001270707	NRM	-1.801360169
16860103	NM_031218	ZNF93	-1.802867453
16826606	NM_001308963	CRNDE	-1.802967428
16676526	NM_001123168	FAM72A	-1.811297261
17009093	NM_001025366	VEGFA	-1.811912558
16746808	NM_002014	FKBP4	-1.812854744
16935396	NM_001278651	RANGAP1	-1.822072039
17113340	OTTHUMT00000057903	GNG5P2	-1.840247741
17004747	NM_017906	PAK1IP1	-1.847123079
16834370	NM_170607	MLX	-1.869617746
17015143	NM_001069	TUBB2A	-1.870408425
16904741	NM_013233	STK39	-1.871618853
16867378	NM_139159	DPP9	-1.872345487
16937943	NM_001134367	SLC6A6	-1.891879236
16706499	NM_005729	PPIF	-1.901340167
16775421	NM_001270952	UCHL3	-1.90939643
16977868	NM_001257386	ABCG2	-1.910764529

17101292	NM_000351	STS	-1.915215412
16840696	NM_006942	SOX15	-1.920160181
16951756	NM_001258379	SLC4A7	-1.922286447
16661832	NM_001199037	SERINC2	-1.927868524
17043529	NM_020156	C1GALT1	-1.928434306
17012879	XR_943040	LOC105378015	-1.933408738
17021217	NM_002395	ME1	-1.935554146
16789524	XR_429419	LOC102723342	-1.93739752
17079971	NM_030780	SLC25A32	-1.945179555
17008769	NM_033112	RRP36	-1.948602741
17077191	XR_928879	LOC100507516	-1.953128225
16854202	NM_001308256	ABHD3	-1.957808966
16697196	NM_052966	FAM129A	-1.96086471
17024746	NM_020861	ZBTB2	-1.966831455
16765513	NM_001127321	CBX5	-1.967408672
16855810	NM_001303618	CD226	-1.967422309
16908985	NM_001039569	AP1S3	-1.976342974
16672279	NM_001286349	KIRREL	-1.982622489
16918244	NM_001191	BCL2L1	-1.983355557
16745563	NM_024769	CLMP	-1.9858179
16728261	NM_053056	CCND1	-1.993476594
16716469	NM_000872	HTR7	-1.994043202
16706117	NM_001284194	FUT11	-2.007101162
16658664	NM_032315	SLC25A33	-2.010707649
16748449	NR_036052	MIR1244-1	-2.011451102
16761116	NR_036052	MIR1244-1	-2.011451102
16988297	NR_036052	MIR1244-1	-2.011451102
16763764	NM_000376	VDR	-2.033074854
17080045	NR_125796	ZFPM2-AS1	-2.048058134
16898403	NM_001128210	SPRED2	-2.049876036
16781684	NM_032572	RNASE7	-2.089821904
16693335	NM_032563	LCE3D	-2.09166721
17005888	NM_003447	ZNF165	-2.094685038
16870506	NM_001145721	HOMER3	-2.094864117
16708880	NR_038940	SH3PXD2A-AS1	-2.099908792
16763968	NR_002951	SNORA2B	-2.101209482
16983800	NM_001258437	TARS	-2.120542405
16881660	NM_006636	MTHFD2	-2.140137267
16997199	NM_001256574	ENC1	-2.145732155
16990203	NR_026705	VTRNA1-3	-2.157055555
16698816	NM_025179	PLXNA2	-2.376222614
16851397	NM_002894	RBBP8	-2.397880894
16673525	NM_001677	ATP1B1	-2.399377237
17022150	NM_022361	POPDC3	-2.400025942
16708910	NM_001002759	SFR1	-2.401334975

17067963	NM_004095	EIF4EBP1	-2.470506183
16734491	NM_003311	PHLDA2	-2.60751099
16869960	NM_001128932	CYP4F11	-2.61193682
16767751	NM_007350	PHLDA1	-2.704402714
17111594	NM_001012968	SPIN4	-2.708836529
16745501	NR_029671	MIR125B1	-2.762765921
16780859	NM_004093	EFNB2	-2.786549922
17043294	NM_003088	FSCN1	-2.886477541
16661862	NM_001204414	TINAGL1	-2.902058631
16995890	NM_001098272	HMGCS1	-3.04128988
17086193	NM_021154	PSAT1	-3.148569835
16732985	NM_001145290	SLC37A2	-3.200232377
17092875	NM_176891	IFNE	-3.267408117
17005862	NM_003536	HIST1H3H	-3.502544386
17016506	NM_003546	HIST1H4L	-3.644674562
17049676	NM_000602	SERPINE1	-3.758638068
17075973	NM_001394	DUSP4	-3.912714921
16852296	NM_001308006	LIPG	-4.275698129
16713187	NM_001024628	NRP1	-4.374060979
16979917	NM_014331	SLC7A11	-4.554481012
